# Diagnostic performance of deep learning for predicting glioma isocitrate dehydrogenase and 1p/19q co-deletion in MRI: a systematic review and meta-analysis

**DOI:** 10.1007/s00330-025-11898-2

**Published:** 2025-08-16

**Authors:** Somayeh Farahani, Marjaneh Hejazi, Mehnaz Tabassum, Antonio Di Ieva, Neda Mahdavifar, Sidong Liu

**Affiliations:** 1 Department of Medical Physics and Biomedical Engineering, School of Medicine, Tehran University of Medical Sciences, Tehran, Iran; 2 Centre for Health Informatics, Australian Institute of Health Innovation, Macquarie University, Sydney, NSW Australia; 3 Computational NeuroSurgery (CNS) Lab, Faculty of Medicine, Health and Human Sciences, Macquarie Medical School, Macquarie University, Sydney, NSW Australia; 4 Department of Epidemiology & Biostatistics, School of Public Health, Tehran University of Medical Sciences, Tehran, Iran

**Keywords:** Glioma, Deep learning, Magnetic resonance imaging, Isocitrate dehydrogenase, Radiomics

## Abstract

**Objectives:**

We aimed to evaluate the diagnostic performance of deep learning (DL)-based radiomics models for the noninvasive prediction of isocitrate dehydrogenase (IDH) mutation and 1p/19q co-deletion status in glioma patients using MRI sequences, and to identify methodological factors influencing accuracy and generalizability.

**Materials and methods:**

Following PRISMA guidelines, we systematically searched major databases (PubMed, Scopus, Embase, Web of Science, and Google Scholar) up to March 2025, screening studies that utilized DL to predict IDH and 1p/19q co-deletion status from MRI data. We assessed study quality and risk of bias using the Radiomics Quality Score and the QUADAS-2 tool. Our meta-analysis employed a bivariate model to compute pooled sensitivity and specificity, and meta-regression to assess interstudy heterogeneity.

**Results:**

Among the 1517 unique publications, 104 were included in the qualitative synthesis, and 72 underwent meta-analysis. Pooled estimates for IDH prediction in test cohorts yielded a sensitivity of 0.80 (95% CI: 0.77–0.83) and specificity of 0.85 (95% CI: 0.81–0.87). For 1p/19q co-deletion, sensitivity was 0.75 (95% CI: 0.65–0.82) and specificity was 0.82 (95% CI: 0.75–0.88). Meta-regression identified the tumor segmentation method and the extent of DL integration into the radiomics pipeline as significant contributors to interstudy variability.

**Conclusion:**

Although DL models demonstrate strong potential for noninvasive molecular classification of gliomas, clinical translation requires several critical steps: harmonization of multi-center MRI data using techniques such as histogram matching and DL-based style transfer; adoption of standardized and automated segmentation protocols; extensive multi-center external validation; and prospective clinical validation.

**Key Points:**

***Question*** *Can DL based radiomics using routine MRI noninvasively predict IDH mutation and 1p/19q co-deletion status in gliomas, and what factors affect diagnostic accuracy?*

***Findings*** *Meta-analysis showed 80% sensitivity and 85% specificity for predicting IDH mutation, and 75% sensitivity and 82% specificity for 1p/19q co-deletion status*.

***Clinical relevance*** *MRI-based DL models demonstrate clinically useful accuracy for noninvasive glioma molecular classification, but data harmonization, standardized automated segmentation, and rigorous multi-center external validation are essential for clinical adoption*.

**Graphical Abstract:**

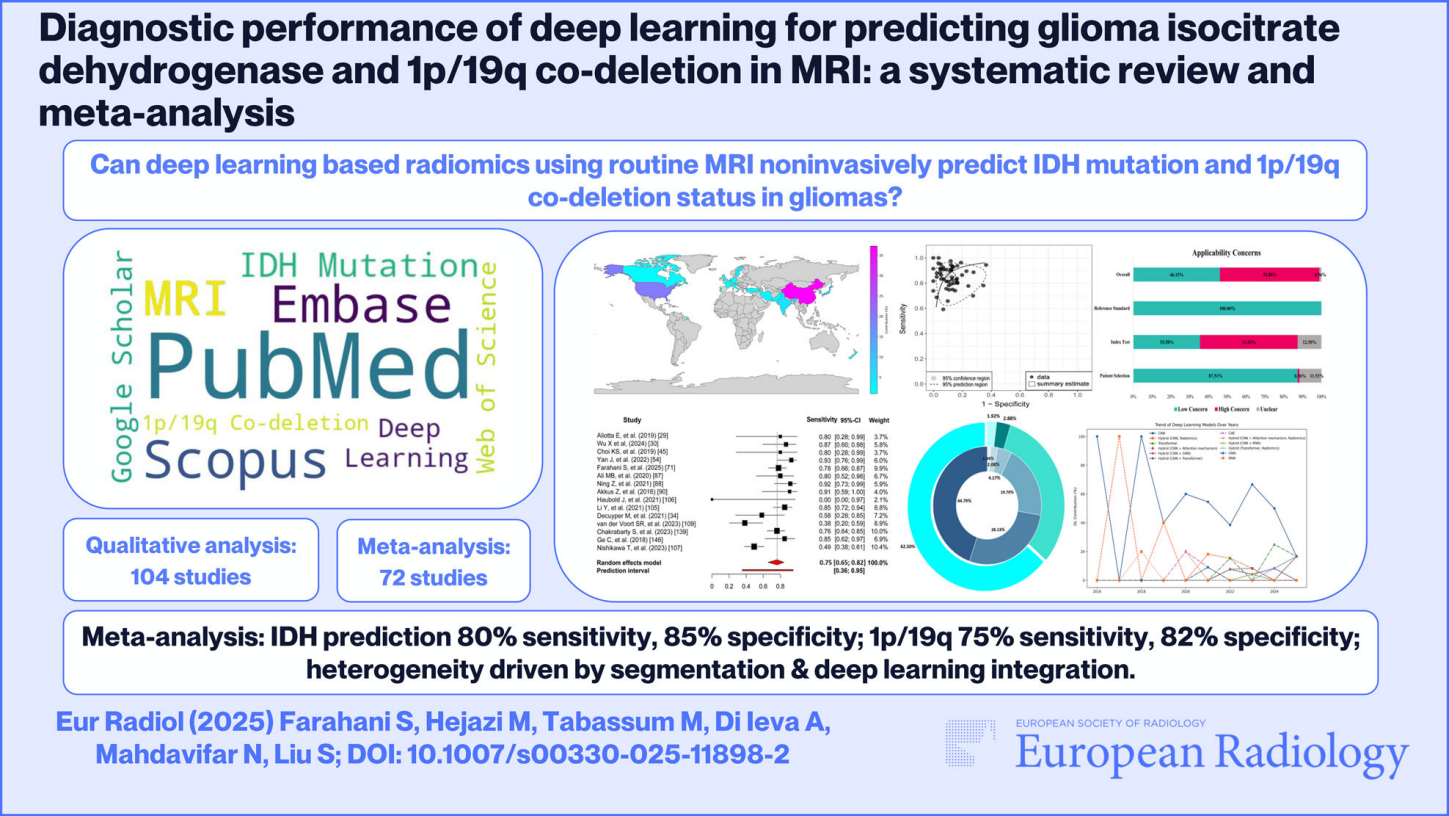

## Introduction

Gliomas, the most common and lethal primary central nervous system (CNS) tumors, show significant histological and molecular variability, making accurate diagnosis essential [1]. Key genetic markers, including isocitrate dehydrogenase (IDH) mutation and 1p/19q co-deletion, guide classification and treatment decisions [2]. Traditional biopsies are invasive and limited by tumor heterogeneity [3]. MRI is central to noninvasive glioma assessment, supported by the European Association of Neuro-Oncology (EANO) guidelines [4]. However, interpreting MRI data can be challenging due to human limitations and radiological “mimics,” which make distinguishing gliomas from conditions such as inflammatory diseases, stroke, and infections difficult [5].

Advancements in radiomics have begun to address these challenges by extracting intricate features from medical images [6]. Radiomics analysis involves two primary methodologies: feature-engineered and deep learning (DL)-based radiomics modeling [7]. The former involves processes such as image segmentation, feature extraction, and statistical analysis, each of which significantly influences subsequent outcomes, particularly in MRI models [8]. Subjective handcrafted features spurred DL integration into radiomics. DL can replace individual steps or operate end-to-end for direct classification [7, 9].

Since the introduction of DL into radiomics, numerous studies have predicted IDH and 1p/19q co-deletion [10–12]. Given the extensive research, there is a critical need for a systematic review to synthesize and thoroughly quantify existing data. Current reviews often focus on conventional radiomics, primarily analyzing radiomic features with machine learning methods. Additionally, some works concentrate solely on specific glioma grades or particular MRI modalities (e.g., dynamic susceptibility contrast (DSC) MR perfusion imaging and T2-FLAIR mismatch) for predicting either IDH mutation or 1p/19q co-deletion, often neglecting the simultaneous prediction of these biomarkers across various glioma grades and imaging techniques [13–17]. To address this gap, our study conducts a comprehensive systematic review and meta-regression to evaluate the accuracy and reliability of DL-based models in predicting IDH mutations and 1p/19q co-deletion using MRI, thereby consolidating evidence on their effectiveness.

## Methods

We performed a PRISMA-guided systematic review and meta-analysis (PROSPERO: CRD42024542505) [18].

### Search strategy and study selection

We systematically searched the PubMed, Scopus, Embase, Web of Science, and Google Scholar for DL-based radiomics studies in glioma up to March 28, 2025, with no time or language restrictions (Supplementary Section 1). We also screened relevant article bibliographies for further identification. The inclusion criteria were studies of gliomas (any World Health Organization grade) that predicted IDH and/or 1p/19q co-deletion status using MRI and incorporated DL algorithms in their radiomics workflow. For inclusion in the meta-analysis, studies had to report sufficient information to allow reconstruction of a 2 × 2 diagnostic table. Those without sufficient validation metrics were restricted to qualitative synthesis. Non-original and non-human studies were excluded. Records were managed via Zotero software (version 6.0.36). Two reviewers (S.F. and M.T.) independently screened the abstracts and full texts in two rounds, resolving disagreements through discussion.

### Data extraction

Two reviewers (S.F. and M.T.) independently collected data on study design, patient characteristics, datasets used, MRI sequences, data augmentation techniques, and computational methodologies using a standardized form (Supplementary Section 2). Performance metrics for constructing the diagnostic confusion matrix were obtained from both internal validation methods (e.g., k-fold and leave-one-out cross-validation) and test datasets, prioritizing external validation cohorts when available, or otherwise using held-out test sets.

When diagnostic table counts were not explicitly reported, we first contacted the corresponding authors by email. If no data were provided, we reconstructed these values from reported sensitivity, specificity, and total sample size using standard formulas. When only receiver-operating characteristic (ROC) curves were available, we extracted sensitivity and specificity at the point closest to the top-left corner (Youden index) using WebPlotDigitizer v4.7. All imputed counts were rounded to the nearest whole number. No imputation was performed for missing clinical or demographic covariates; these data were excluded from subgroup analyses. This process aligns with Cochrane Handbook guidance for transparency and reproducibility [19]. In publications reporting multiple DL models or MRI modalities, the best-performing model was selected for the meta-analysis. However, the full range of results was included and analyzed separately in the subgroup analyses.

### Quality assessment

The risk of bias and applicability concerns were evaluated via a modified QUADAS-2 tool [20], which incorporates relevant items from the Checklist for Artificial Intelligence in Medical Imaging (CLAIM) and the radiomics quality score (RQS). Key considerations included clarity in imaging protocols, appropriate data selection and missing data handling, use of reliable reference standards, and avoidance of severe genotype imbalances. Additionally, the index test evaluation assessed the use of multiple segmentations and the robustness of the model predictions. Concerns about applicability, particularly regarding validation on external datasets, were addressed to ensure generalizability across diverse clinical settings. If the data were insufficient, we contacted the authors for clarification via email. Moreover, the methodologies, strengths, limitations, quality, and translatability of studies were evaluated using RQS, which assesses each study on 16 components, with cumulative scores ranging from −8 to 36 [21]. Three reviewers (S.F. and N.M. for QUADAS-2 and S.F. and M.T. for RQS) independently conducted assessments, resolving discrepancies through discussion (Supplementary, Sections 3 and 4).

### Statistical analysis

A bivariate random effects model was used to pool the sensitivity, specificity, and 95% confidence intervals (CIs) across studies (≥ 5) and summary receiver operating characteristic (SROC) curves. Heterogeneity was evaluated using Cochran's *Q*-test, the *I*^2^ statistic, prediction intervals (*p* < 0.05), and the Spearman correlation coefficients (SCC) between sensitivity and the false positive rate (threshold effect indicated by an SCC > 0.6) [22, 23]. Subgroup analyses explored sources of heterogeneity by subgroup analyses in instances with enough studies [24]. A leave-one-out meta-analysis assessed each study's impact on effect size. Publication bias was evaluated using funnel plots and Egger’s test. The Trim and Fill method by Duval and Tweedie was applied to adjust pooled sensitivity and specificity estimates in the presence of asymmetry. Statistical power was also calculated across effect sizes [25]. Analyses were performed with R packages ‘mada’, ‘metameta’, ‘metafor’ (R v4.4.1), and MetaBayesDTA (v1.5.2) [26].

## Results

### Study characteristics

**Fig. 1:**
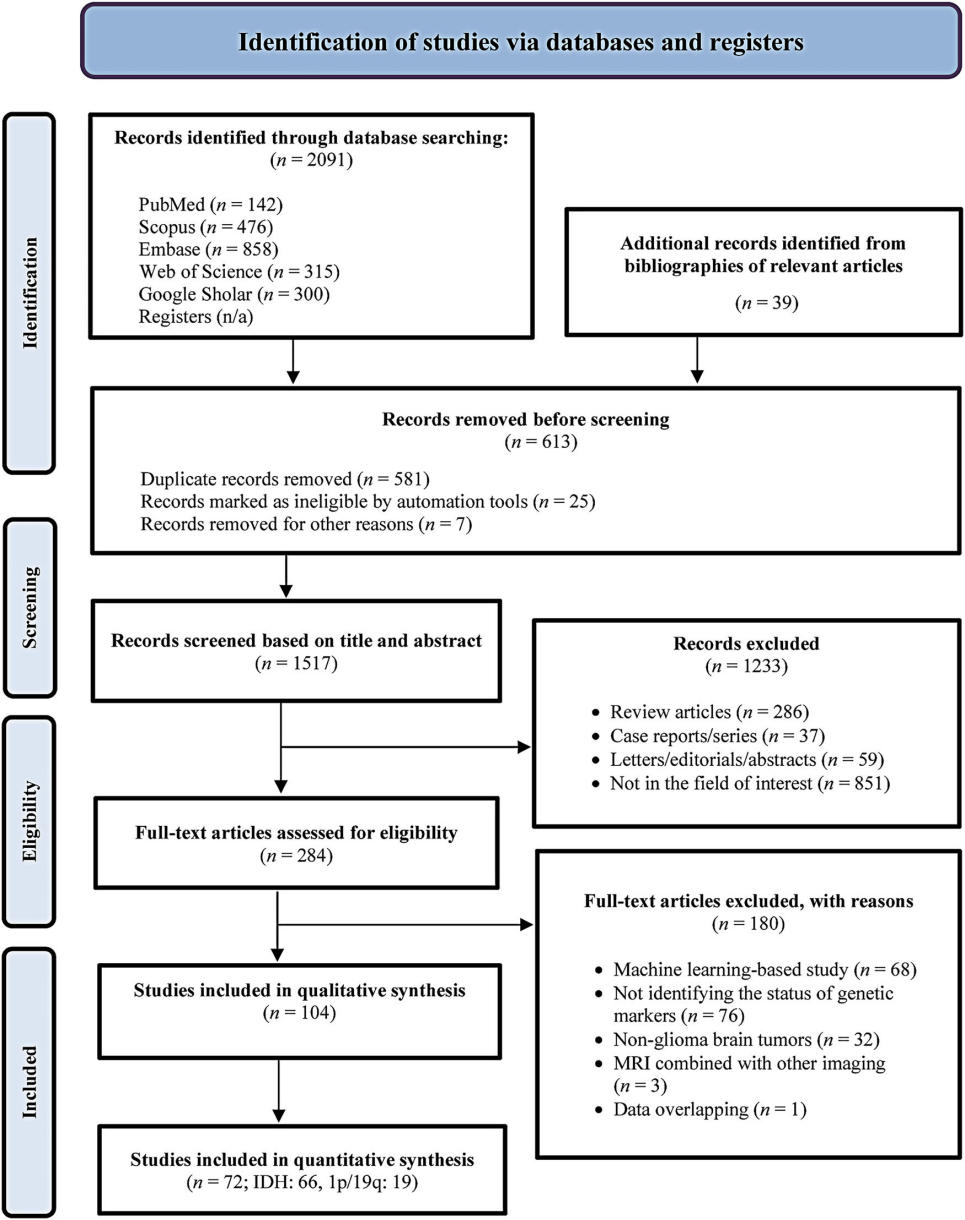
Flow diagram of the study selection process

A total of 1517 unique publications were initially identified through primary searches and relevant study bibliographies. Following screening and full-text reviews, 104 studies were eligible for qualitative analysis, of which 72 were included in the meta-analysis (Fig. 1). One study [27] was excluded because it served solely for the external validation of another study [28]. Details on the inclusion and exclusion status of studies in the meta-analysis are summarized in Supplementary Table 4.

**Fig. 2:**
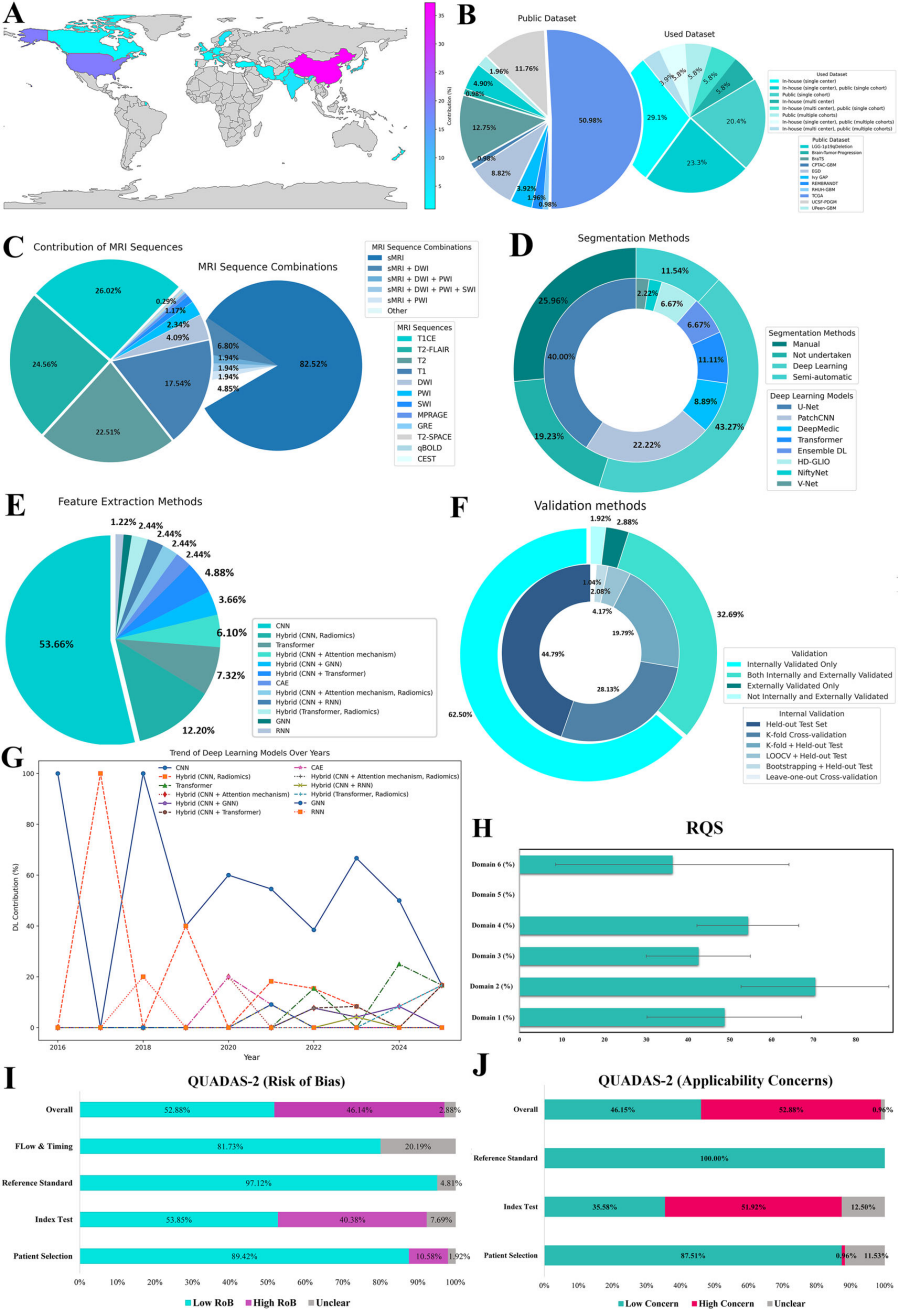
**A** Heatmap depicting global publication trends in MRI-based DL models for predicting IDH and 1p/19q codeletion status. **B** Distribution of dataset types in our included studies, highlighting the prevalence of public, in-house, and combined data sources. **C** Distribution of MRI sequences used in our included studies, highlighting the diversity and frequency of sequence combinations employed in research. **D** Breakdown of segmentation methods used in 104 studies. The outer ring displays the overall segmentation techniques, whereas the inner donut highlights the distribution of DL methods. **E** Distribution of DL architectures used for feature extraction, highlighting the predominance of CNN-based models. **F** Validation methods employed in the studies. The outer ring shows the overall approach to validation, while the inter ring details specific internal validation methods. **G** Temporal trends (2016–2025) in DL model choice in feature extraction, illustrating the shift from CNN-only workflows to autoencoders, transformers, and other hybrids. **H** Mean RQS ± SE across six quality domains (D1 (protocol quality), D2 (feature selection and validation), D3 (biologic/clinical validation and utility), D4 (model performance index), D5 (level of evidence), and D6 (open science and data)). **I** QUADAS-2 risk-of-bias profile for the included studies. **J** QUADAS-2 applicability concerns, with high concern in 55 studies. IDH, isocitrate dehydrogenase; CEST, chemical-exchange saturation transfer; DWI, diffusion-weighted imaging; DSC-PWI, dynamic susceptibility-contrast perfusion-weighted imaging; MRAGE, magnetization-prepared rapid acquisition gradient-echo; qBOLD, quantitative blood-oxygen-level-dependent; sMRI, structural MRI; SWI, susceptibility-weighted imaging; TR-SPACE, T2 sampling perfection with application-optimized contrasts using different flip-angle evolutions; DL, deep learning; AE, autoencoder; CNN, convolutional neural network; GNN, graph neural network; nnUNet, no-new-net U-Net framework; RNN, recurrent neural network; VNet, volumetric neural network; LOOCV, leave-one-out cross-validation; ROI, region of interest; QUADAS-2, quality assessment of diagnostic accuracy studies, version 2; LoB, risk of bias; RQS, radiomics quality score; D, domain; TCGA, the Cancer Genome Atlas; Ivy GAP, Ivy Glioblastoma Atlas Project; RHUH-GBM, Río Hortega University Hospital Glioblastoma Dataset; UPenn-GBM, University of Pennsylvania Glioblastoma Dataset; UCSF-PDGM, University of California, San Francisco Preoperative Diffuse Glioma MRI; EGD, Erasmus Glioma Database; LGG-1p19qDeletion, LGG-1p19q deletion dataset

Our analysis revealed that China and the USA dominate global research in this field, significantly outpacing other countries' publication volume (Fig. 2A). Additionally, the surveyed studies spanned various sample sizes, ranging from 41 [29] to 2776 patients [30]. Over 23% of the included studies employed genotyping methods, such as immunohistochemistry, DNA sequencing, or fluorescence in situ hybridization, to directly assess genetic variants. In contrast, 44% used molecular typing, which classifies tumors into clinically relevant subgroups based on their genotyping results [2]. Approximately 20% of studies combined both approaches by integrating multiple cohorts, whereas 12% did not report the reference standard used to determine biomarker status (Table 1).

**Table 1:** Characteristics of the 104 included studies, all of which employed a retrospective design

Study	Total no. pts	Genes	Grade	Reference standard	Dataset(s)	MRI	Segmentation	Feature extraction	Validation	AUC
Choi et al [45]	463	IDH, 1p/19q	2, 3, 4	IDH: IHC, 1p/19q: FISH	In-house (single center)	T1, T1CE, T2, T2-FLAIR, DSC	CNNs	RNNs	Internally validated only	IDH: 95%, 1p/19q: 89%
Fukuma et al [31]	164	IDH	2, 3	IDH1/2: Sanger sequencing, pyrosequencing	In-house (multi-center)	T1, T1CE, T2, T2-FLAIR	Manual	Hybrid (CNNs, Radiomics)	Internally validated only	IDH: 69.9%
Ge et al [36]	167	IDH	2, 3, 4	TCGA	Public (TCGA)	T1, T1CE, T2, T2-FLAIR	Manual	CNN with attention-weighted feature fusion	Internally validated only	IDH: 88.82%*
Li et al [10]	151	IDH	2	IDH1: Sanger sequencing	In-house (single center)	T1CE, T2-FLAIR	CNNs	Hybrid (CNNs, Radiomics)	Internally validated only	IDH: 96.15%
Liang et al [32]	167	IDH	2, 3, 4	TCGA	Public (TCGA)	T1, T1CE, T2, T2-FLAIR	Manual	3D DenseNet	Internally validated only	IDH: 85.7%
Wu et al [129]	105	IDH	NR	NR	In-house (single center)	T1CE, T2-FLAIR	CNNs	Radiomics	Internally validated only	IDH: 88%
Kim et al [12]	143	1p/19q	2, 3, 4	BraTS 2017	Public (BraTS 2017)	T1, T1CE, T2-FLAIR	Manual	Hybrid (CNNs, Radiomics)	Internally validated only	1p/19q: 69.1%
Chang et al [11]	259	IDH, 1p/19q	2, 3, 4	TCGA	Public (TCGA)	T1, T1CE, T2, T2-FLAIR	Manual	CNNs	Internally validated only	IDH: 91%, 1p/19q: 88%
Ali et al [87]	161	IDH, 1p/19q	2	NR	In-house (multi-center)	T1CE, T2-FLAIR	Not undertaken (whole MRI slices)	Multi-stream convolutional autoencoder (CAE)	Internally validated only	IDH: 81.19%, 1p/19q: 74.81%*
Tang et al [33]	93	IDH, 1p/19q	4	Genomic sequencing	In-house (single center)	T1CE, DTI	Radiomics (manual); DL (not undertaken, bounding box)	CNNs	Internally validated only	IDH: 94.6%, 1p/19q: 88.1%*
Decuyper et al [34]	466	IDH, 1p/19q	2, 3, 4	TCGA, In-house dataset: IDH: IHC; 1p19q: FISH	In-house (single center), public (TCGA, LGG-1p19qDeletion, BraTS 2019)	T1, T1CE, T2, T2-FLAIR	U-Net	CNNs	Both internally and externally	IDH: 86.23%, 1p/19q: 86.61%
Ning et al [88]	645	IDH, 1p/19q	2, 3, 4	TCGA, In-house dataset: NR	In-house (single center), public (TCGA)	T1CE, T2-FLAIR	Manual	CAE	Externally validated only	IDH: 92.4%, 1p/19q: 90.2%
van der Voort et al [130]	1748	IDH, 1p/19q	2, 3, 4	REMBRANDT, CPTAC-GBM, Ivy GAP, BraTS, Brain-Tumor-Progression, In-house dataset: NR	In-house (multi-center), public (REMBRANDT, CPTAC-GBM, Ivy GAP, BraTS, Brain-Tumor-Progression)	T1, T1CE, T2, T2-FLAIR	CNNs	Multi-task CNNs	Both internally and externally	IDH: 90%, 1p/19q: 85%
Cluceru et al [42]	531	IDH, 1p/19q	2, 3, 4	TCGA, UCSF	Public (TCGA, UCSF)	T1CE, T2, T2-FLAIR, ADC	Manual	CNNs	Both internally and externally	Overall: 85.70%*
Haubold et al [106]	145	IDH, 1p/19q	2, 3, 4	NR	In-house (single center)	T1, T1CE, T2-FLAIR	DeepMedic	Radiomics	Internally validated only	IDH: 86.1%, 1p/19q: 71.1%
Tupe-Waghmare et al [35]	269	IDH, 1p/19q	4	TCGA, In-house dataset: NR	In-house (single center), public (TCGA)	T1CE, T2, T2-FLAIR	DeepMedic	ResNet50	Internally validated only	IDH: 80.04%, 1p/19q: 88.73%*
Matsui et al [52]	217	IDH, 1p/19q	2, 3	IDH: IHC, 1p19q: FISH	In-house (single center)	T1, T2, T2-FLAIR	Not undertaken (bounding box)	ResNet	Internally validated only	NR
Chang et al [51]	496	IDH	2, 3, 4	TCGA, In-house dataset: IHC, next-generation sequencing, mass spectrometry–based mutation genotyping (OncoMap), capture-based sequencing (OncoPanel)	In-house (multi-center), public (TCGA)	T1, T1CE, T2, T2-FLAIR	Not undertaken (bounding box)	ResNet	Internally validated only	IDH: 91%
Calabrese et al [49]	256	IDH	4	TCGA, Genetic sequencing	In-house (single center), public (TCGA‐GBM)	T1, T1CE, T2, T2-FLAIR, SWI, DWI, ASL, DTI	CNNs	Radiomics	Both internally and externally validated	IDH: 63%
Ai et al [77]	235	IDH	2, 3, 4	BraTS 2019, TCGA	Public (BraTS-2019, TCGA)	T2-FLAIR	TDABNet	TDABNet	Internally validated only	IDH: 96.44%
Chaddad et al [101]	83	IDH, 1p/19q	2, 3	TCGA	Public (TCGA)	T1, T1CE, T2, T2-FLAIR	Semi-automatic	CNNs	Internally validated only	IDH: 70.0%
Chakrabarty et al [131]	1047	IDH, 1p/19q	2, 3, 4	TCGA, BraTS, Ivy GAP, EGD, LGG-1p19qDeletion, In-house dataset: IDH: IHC, 1p19q: FISH	In-house (single center), public (TCGA, BraTS, Ivy GAP, EGD, LGG-1p19qDeletion)	T1CE, T2, T2-FLAIR	CNNs	CNNs	Both internally and externally validated	IDH: 93.3%, 1p/19q: 84.2%
Chakrabarty et al [91]	546	IDH	2, 3, 4	TCGA, BraTS, Ivy GAP, In-house dataset: NR	In-house (single center), public (TCGA, BraTS, Ivy GAP)	T1CE, T2, T2-FLAIR	3D Mask R-CNN	3D Mask R-CNN	Both internally and externally validated	IDH: 87.1%
Chen et al [82]	271	IDH, 1p/19q	NR	BraTS, In-house dataset: NR	In-house (single center), public (BraTS)	T1, T1CE, T2, T2-FLAIR	Not undertaken (bounding box)	WSOFNet	Both internally and externally validated	IDH: 96.55%
Chu et al [92]	190	IDH	2, 3, 4	BraTS 2019	Public (BraTS 2019)	T1, T1CE, T2, T2-FLAIR	UNet++	UNet++	Internally validated only	IDH: 95.22%^*^
Buz-Yalug et al [46]	162	IDH	4	Minisequencing	In-house (single center)	T1, T1CE, T2, DSC	Manual	ResNet50, VGG16	Internally validated only	IDH: 89%
Calabrese et al [50]	400	IDH	4	UCSF	Public (UCSF)	T1, T1CE, T2, T2-FLAIR, SWI, ASL, DWI, HARDI	CNNs	Hybrid (CNNs, Radiomics)	Internally validated only	IDH: 96%
Cheng et al [79]	218	IDH	2, 3, 4	BraTS 2020	Public (BraTS 2020)	T1, T1CE, T2, T2-FLAIR	Manual	Hybrid CNN-Transformer encoder	Internally validated only	IDH: 90.37%
Choi et al [95]	136	IDH	4	TCGA, In-house dataset: Sanger sequencing	In-house (single center), public (TCGA)	T2	V-Net	Radiomics	Externally validated only	IDH: 85.7%
Choi et al [28]	1166	IDH, 1p/19q	2, 3, 4	TCGA, In-house dataset: NR	In-house (multi-center), public (TCGA)	T1CE, T2, T2-FLAIR	U-Net	Hybrid (CNNs, Radiomics)	Both internally and externally validated	IDH: 94%
Gore et al [132]	217	IDH	2, 3, 4	TCGA	Public (TCGA)	T1, T1CE, T2, T2-FLAIR	Not undertaken (cropped tumor-bearing regions)	CNNs	Internally validated only	IDH: 93.67%*
Karami et al [43]	146	IDH, 1p/19q	2, 3, 4	NR	In-house (single center)	T1CE, T2, T2-FLAIR, multi-shell diffusion	Semi-automatic (HD-GLIO)	ResNet10	Internally validated only	IDH: 75%, 1p/19q: 72%*
Liu et al [98]	78	IDH	4	NR	In-house (single center)	T1, T1CE, T2, T2-FLAIR, DSC, DWI	nnU-Net	Radiomics (quantitative radiological parameters)	Not internally and externally validated	IDH: 81.5%
McHugh et al [59]	1158	IDH, 1p/19q	2, 3, 4	TCGA, EGD, In-house dataset: IDH: IHC, genetic sequencing; 1p19q: FISH	In-house (single center), public (TCGA, EGD)	T1CE, T2, T2-FLAIR	Dense U-Net	2D Dense U-Nets	Both internally and externally validated	IDH: 95.8%, 1p/19q: 85.4%
Moon et al [53]	878	IDH	2, 3, 4	TCGA, In-house dataset: NR	In-house (single center), public (TCGA)	T1CE, T2-FLAIR	U-Net	ResNet50	Both internally and externally validated	IDH: 83.3%
Nalawade et al [62]	260	IDH	2, 3, 4	TCGA	Public (TCGA)	T2	Not undertaken (whole MRI slices)	DenseNet-161, ResNet-50, Inception-v4	Internally validated only	IDH: 84%
Nalawade et al [60]	368	IDH, 1p/19q	2, 3, 4	TCGA	Public (TCGA)	T2	Dense U-Net	3D Dense-Unet	Internally validated only	IDH: 68%, 1p/19q: 82%*
Pasquini et al [133]	100	IDH	4	IHC, Sanger sequencing	In-house (single center)	T1, T2, T2-FLAIR, MPRAGE, rCBV, ADC	Not undertaken (Bounding box)	CNNs	Internally validated only	IDH: 86%*
Rui et al [103]	42	IDH	2, 3, 4	IHC	In-house (single center)	T1CE, T2-FLAIR, QSM	Semi-automatic (ITK-SNAP)	CNNs	Internally validated only	IDH: 89%*
Safari et al [134]	105	IDH	2, 3	TCGA	Public (TCGA)	T1, T1CE, T2, T2-FLAIR	Semi-automatic	Shuffle-ResNet	Internally validated only	IDH: 94.3%
Zhang et al [84]	759	IDH	2, 3, 4	TCGA, In-house dataset: IHC, Genomic sequencing	In-house (multi-center), public (TCGA)	T1, T1CE, T2, T2-FLAIR	nnU-Net	MFEFnet	Both internally and externally validated	IDH: 85.64%
Zhang et al [66]	486	IDH	2, 3, 4	TCGA, In-house dataset: IHC, Sanger sequencing	In-house (single center), public (TCGA)	T1, T1CE, T2, T2-FLAIR	nnU-Net	Radiomics	Internally validated only	IDH: 92%
Yogananda et al [135]	1849	IDH	NR	TCIA, EGD, UCSF, In-house dataset: Sanger sequencing, next-generation sequencing, IHC	In-house (multi-center), public (TCGA, EGD, UCSF)	T1, T1CE, T2, T2-FLAIR	FeTS tool, nnU-Net	nnU-Net	Both internally and externally validated	IDH: 96.46%
Zeng et al [67]	110	IDH	2, 3, 4	BraTS 2019, TCGA, In-house dataset: NR	In-house (single center), public (BraTS 2019)	T1, T1CE, T2, T2-FLAIR	MDAS framework	Hybrid (CNNs, Radiomics)	Internally validated only	IDH: 86%
Xu et al [72]	188	IDH	2, 3, 4	IHC	In-house (single center)	T1CE, T2	Not undertaken (whole MRI slices)	Vision Transformer (ViT)	Internally validated only	IDH: 98.2%
Wu et al [70]	493	IDH	2, 3, 4	TCGA, In-house dataset: IHC	In-house (single center), public (TCGA)	T2	Manual + Bounding box	Swin Transformer, ResNet-101	Both internally and externally validated	IDH: 87.8%
Wei et al [89]	372	IDH	NR	TCGA, Ivy-GAP, In-house dataset: NR	In-house (single center), public (TCGA, Ivy-GAP)	T1, T1CE, T2, T2-FLAIR	Manual	Graph Neural Network (GNN)	Internally validated only	IDH: 86.6%*
Wang et al [76]	121	IDH	2, 3, 4	TCGA, BRATS 2020	Public (TCGA, BraTS 2020)	T1, T1CE, T2, T2-FLAIR	3D U-Net	SGPNet	Internally validated only	IDH: 94.9%
Wei et al [78]	372	IDH	NR	TCGA, Ivy-GAP, In-house dataset: NR	In-house (single center), public (TCGA, Ivy-GAP)	T1, T1CE, T2, T2-FLAIR	Semi-automatic	Hybrid (CNNs, GNN)	Internally validated only	IDH: 89.2%
Wei et al [81]	387	IDH	NR	TCGA, In-house dataset: NR	In-house (single center), public (TCGA)	T1, T1CE, T2, T2-FLAIR	nnU-Net	Hybrid (CNNs, GNN)	Internally validated only	IDH: 90.5%
Tripathi et al [136]	377	IDH, 1p/19q	2, 3, 4	TCGA-LGG, TCGA-GBM, LGG-1p19qDeletion	Public (TCGA-LGG, TCGA-GBM, TCGA-1p19qdeletion)	T1, T1CE, T2, T2-FLAIR	CNNs	CNNs	Internally validated only	IDH: 91.96%, 1p/19q: 87.88%*
Shi et al [65]	489	IDH	NR	Sanger sequencing	In-house (single center)	T1, T1CE, T2, T2-FLAIR	Manual	Hybrid (SA-Net (self-attention network), Radiomics)	Internally validated only	IDH: 81%
Shi et al [83]	218	IDH	NR	BraTS 2020	Public (BraTS 2020)	T1, T1CE, T2, T2-FLAIR	TransBTS	3D U-Net encoder with transformer (TransBTS)	Internally validated only	IDH: 90.3%
Yan et al [54]	555	IDH, 1p/19q	2, 3	TCGA, In-house dataset: IDH: IHC, 1p19q: FISH	In-house (single center), public (TCGA)	T1, T1CE, T2, T2-FLAIR	Manual	Deep CNN (ResNet-34 based)	Both internally and externally validated	1p/19q: 98.3%
Kihira et al [104]	239	IDH	2, 3, 4	IHC	In-house (multi-center)	T2-FLAIR	U-Net	Radiomics	Both internally and externally validated	IDH: 93%
Sohn et al [137]	418	IDH	4	Next-generation sequencing	In-house (single center)	T1, T1CE, T2, T2-FLAIR	Semi-automatic (HD-GLIO)	Radiomics	Internally validated only	IDH: 96.4%
Buda et al [138]	110	IDH, 1p/19q	2, 3	TCGA	Public (TCGA)	T1, T1CE, T2, T2-FLAIR	U-Net	Radiomics	Internally validated only	NR
Ali et al [80]	167	IDH	2, 3, 4	TCGA, In-house dataset: NR	In-house (single center), public (TCGA)	T1CE, T2-FLAIR	Manual	EtFedDyn with an attention-weighted fusion layer	Internally validated only	IDH: 85.46%*
Chen et al [73]	1153	IDH, 1p/19q	NR	TCGA, In-house dataset: NR	In-house (single center), public (TCGA)	T1, T1CE, T2, T2-FLAIR	Not undertaken (whole MRI slices)	Vision Transformer	Both internally and externally validated	NR
Elyassirad et al [55]	495	IDH	2, 3, 4	UCSF	Public (UCSF)	T1, T1CE, T2-FLAIR	Manual	ResNet	Internally validated only	IDH: 90.96%
Fayyaz et al [93]	89	IDH	2, 3, 4	TCGA	Public (TCGA)	NR	Not undertaken (whole MRI slices)	CNNs (Xception, ResNet152V2, InceptionV3, InceptionResNetV2, NASNetLarge)	Internally validated only	IDH: 99%
Ge et al [139]	159	1p/19q	2	NR	In-house (single center)	T1CE, T2	Not undertaken (mask-enhanced whole MRI slices)	CNNs	Internally validated only	1p/19q: 89.39%
Gómez Vecchio et al [56]	469	IDH	2, 3	EGD, In-house dataset: NR	In-house (multi-center), public (EGD)	T1CE, T2, T2-FLAIR	Semi-automatic	ResNet152	Both internally and externally validated	NR
Hosseini et al [37]	57	IDH	4	IHC, RealTime PCR	In-house (single center)	T1CE, T2-FLAIR	Semi-automatic	Radiomics	Internally validated only	IDH: 92%
Jeon et al [96]	218	IDH, 1p/19q	2, 3	NR	In-house (single center)	T1, T1CE, T2, T2-FLAIR	Semi-automatic (HD-GLIO)	Radiomics (quantitative radiological parameters)	Not internally and externally validated	IDH: 69%
Jian et al [140]	418	IDH	2, 3, 4	UCSF	Public (UCSF-PDGM)	T1, T1CE, T2, T2-FLAIR	BraTS-based ensemble model	Radiomics	Internally validated only	IDH: 80.18%
Li et al [63]	402	IDH	2, 3, 4	NR	In-house (multi-center)	T2	Manual	Hybrid (ResNet101, Radiomics)	Both internally and externally validated	IDH: 98%
Li et al [57]	263	IDH	2, 3, 4	IHC, Genome sequencing	In-house (single center)	T1, T1CE, T2, T2-FLAIR	Manual	ResNet50	Internally validated only	IDH: 87.1%
Li et al [105]	1016	IDH, 1p/19q	2, 3, 4	IDH: IHC, Pyrosequencing; 1p/19q: FISH	In-house (single center)	T1, T1CE, T2	Manual	Hybrid (ResNet18, Radiomics)	Internally validated only	IDH: 89%, 1p/19q: 85%
Lost et al [141]	584	IDH	2, 3, 4	IHC ± Sanger sequencing	In-house (multi-center)	T1CE, T2-FLAIR, PGSE, GRE	U-Net Transformer (UNETR)	Radiomics	Both internally and externally validated	IDH: 83.5%
Nishikawa et al [107]	460	IDH, 1p/19q	2, 3, 4	TCGA, In-house dataset: exome sequencing; Sanger sequencing; 1p/19q: multiplex ligation-dependent probe amplification (MLPA)	In-house (single center), public (TCGA)	T2	Manual	CNNs	Both internally and externally validated	IDH: 70.3%, 1p/19q: 65.1%*
Park et al [38]	162	IDH	2, 3	IHC, pyrosequencing	In-house (single center)	T1CE, T2-FLAIR	Not undertaken (whole MRI slices)	Radiomics (radiological parameters)	Internally validated only	IDH: 82.1%
Sacli-Bilmez et al [47]	225	IDH	2, 3, 4	Minisequencing, Sanger sequencing	In-house (single center)	T1, T1CE, T2, 1H‐MRS, DSC	Manual	CNNs	Internally validated only	IDH: 87.8%*
Sairam et al [142]	58	IDH	3, 4	TCGA	Public (TCGA)	T1, T2, T2-FLAIR	Not undertaken (cropped tumor-bearing regions)	CNNs	Internally validated only	IDH: 99.1%
Santinha et al [143]	231	IDH	2, 3, 4	TCGA, In-house dataset: NR	In-house (multi-center), public (TCGA)	T1, T1CE, T2, T2-FLAIR	Automatic (HD-GLIO)	Radiomics	Both internally and externally validated	IDH: 83.6%
Shi et al [64]	488	IDH	NR	Sanger sequencing	In-house (single center)	T1, T2, T1CE, T2-FLAIR	Manual	Hybrid (SA-Net, Radiomics)	Internally validated only	IDH: 82%
Stadlbauer et al [48]	215	IDH	2, 3, 4	NR	In-house (multi-center)	T1CE, T2-FLAIR, DWI, GE-DSC, multi-echo GE & SE (qBOLD), SE-DSC, GESE-DSC	Semi-automatic	Radiomics	Both internally and externally validated	IDH: 87.9%
Taha et al [99]	326	IDH	4	TCGA, In-house dataset: next-generation sequencing, IHC	In-house (single center), public (TCGA)	T1CE	Semi-automatic	Radiomics	Both internally and externally validated	NR
Usuzaki et al [69]	597	IDH	2, 3, 4	UCSF-PDGM, UPeen-GBM	Public (UCSF-PDGM, UPeen-GBM)	T1CE	BraTS challenge model	Hybrid (vViT, Radiomics)	Both internally and externally validated	IDH: 88.7%
Wang et al [97]	627	IDH	2, 3, 4	TCGA, In-house dataset: IHC	In-house (single center), public (TCGA)	T1, T1CE, T2, T2-FLAIR	nnU-Net	Radiomics	Both internally and externally validated	IDH: 86%
Wankhede et al [144]	253	IDH, 1p/19q	2	NR	NR	T1CE, T2-FLAIR	Not undertaken (whole MRI slices)	CNNs	Internally validated only	NR
Yang et al [86]	811	IDH, 1p/19q	2, 3, 4	TCGA-LGG, TCGA-GBM, UCSF-PDGM, EGD, In-house dataset: NR	In-house (single center), public (TCGA-LGG, TCGA-GBM, UCSF-PDGM, EGD)	T1, T1CE, T2, T2-FLAIR	Manual	Hybrid (CNNs, Swin Transformer)	Both internally and externally validated	IDH: 73.6%
Yu et al [74]	664	IDH	2, 3, 4	TCGA, In-house dataset: CRISPR/Cas12a-based assay, droplet digital PCR (ddPCR), and Sanger sequencing	In-house (multi-center), public (TCGA)	T1, T1CE, T2, T2-FLAIR	Manual	Vision Transformer (ViT)	Both internally and externally validated	IDH: 89%
Yuan et al [40]	226	IDH	2, 3, 4	IDH: Sanger sequencing; 1p/19q: qPCR assay	In-house (single center)	T1, T2, T2-FLAIR, DTI	Semi-automatic	VGG16	Internally validated only	IDH: 83.4%
Yuan et al [145]	84	IDH	1, 2, 3, 4	Next-generation sequencing, IHC	In-house (single center)	CEST, MPRAGE, T2-SPACE	Manual	Hybrid (CNNs, Radiomics)	Internally validated only	IDH: 88.68%
Zhang et al [100]	162	IDH	2, 3, 4	NR	In-house (single center)	T1, T1CE, T2, T2-FLAIR	Automatic (NiftyNet)	Radiomics	Internally validated only	IDH + MGMT: 95%
Zhang et al [146]	502	IDH	NR	NR	In-house (single center)	T1, T1CE, T2, T2-FLAIR	Manual	SE-Net	Internally validated only	IDH: 75.25%*
Zhao et al [147]	150	1p/19q	2, 3, 4	TCGA, In-house dataset: NR	In-house (single center), public (TCGA)	T1, T1CE, T2, T2-FLAIR	Manual	Custom U-net, ResNet152	Internally validated only	1p/19q: 92.15%*
Zhu et al [44]	539	IDH	2, 3, 4	UCSF-PDGM, In-house dataset: Sanger sequencing	In-house (single center), public (UCSF-PDGM)	T1, T1CE, T2, T2-FLAIR, ADC, SWI	Ensemble deep learning model	Radiomics	Both internally and externally validated	IDH: 88.7%
Aliotta et al [29]	41	IDH, 1p/19q	2, 3	IDH: IHC ± DNA pyrosequencing; 1p/19q: FISH and chromosomal microarray analysis (OncoScan)	In-house (single center)	T1, T1CE, T2, T2-FLAIR, DTI	DeepMedic	Radiomics	Internally validated only	IDH: 90%, 1p/19q: 94%
Alom et al [148]	78	IDH, 1p/19q	2, 3, 4	TCGA	Public (TCGA)	T1, T1CE, T2, T2-FLAIR	Radiomics (manual); DL (not undertaken, whole MRI slices)	Hybrid (VGG-Net, ResNet50, and DenseNet, Radiomics)	Internally validated only	IDH: 80.73%, 1p/19q: 87.21%*
González et al [61]	99	IDH1, 1p19q	2	TCGA	Public (TCGA)	T1, T1CE, T2, T2-FLAIR	Not undertaken (whole MRI slices)	Inception v3	Internally validated only	NR
Riahi Samani et al [39]	275	IDH	4	NR	In-house (single center)	T1, T1CE, T2, T2-FLAIR, DTI	DeepMedic	CNNs	Internally validated only	NR
Sun et al [149]	424	IDH, 1p/19q	2, 3, 4	UCSF, In-house dataset: NR	In-house (single center), public (UCSF)	T1CE, T2-FLAIR	U-Net	Radiomics	Internally validated only	96%
Zhao et al [41]	202	IDH	2, 3, 4	TCGA, In-house dataset: NR	In-house (single center), public (TCGA)	T1CE, T2, T2-FLAIR, DWI	Not undertaken (whole MRI slices)	UCNet/F-UCNet	Both internally and externally validated	IDH: 95.63%*
Zhang et al [75]	466	IDH, 1p/19q	2, 3, 4	TCGA, In-house dataset: NR	In-house (single center), public (TCGA)	T1CE, T2-FLAIR	Not undertaken (cropped tumor-bearing regions)	Vision Transformer with cross-attention, DenseNet-121	Both internally and externally validated	IDH: 92%, 1p/19q: 99%
Yogananda et al [58]	368	1p/19q	2, 3, 4	TCGA	Public (TCGA)	T2	IDH network	3D Dense-UNet	Internally validated only	1p/19q: 95%
Akkus et al [90]	159	1p/19q	2, 3	FISH	In-house (single center)	T1CE, T2	Semi‐automatic	CNNs	Internally validated only	1p/19q: 87.7%*
Cao et al [85]	954	IDH, 1p/19q	2, 3, 4	TCGA, EGD; In-house dataset: IHC, DNA sequencing	In-house (multi-center), public (TCGA, EGD)	T1CE, T2-FLAIR	U-Net	Hybrid (ResNet, Graph Convolutional Network)	Both internally and externally validated	IDH: 85%, 1p/19q: 81%
Hu et al [150]	256	IDH	2, 3, 4	PCR amplification, Sanger sequencing	In-house (single center)	T1, T1CE, T2	Manual	DenseNet	Internally validated only	IDH: 90.4%
Farahani et al [71]	1705	IDH, 1p/19q	2, 3, 4	TCGA, UCSF-PDGM, Ivy GAP, LGG-1p/19q, RHUH-GBM, UPenn-GBM, EGD	Public (TCGA, UCSF-PDGM, Ivy GAP, LGG-1p/19q, RHUH-GBM, UPenn-GBM, EGD)	T1, T1CE, T2, T2-FLAIR	MTS-UNET	MTS-UNET (SWIN-UNETR backbone)	Both internally and externally validated	IDH: 90.58%, 1p/19q: 69.22%
Niu et al [68]	1185	IDH	2, 3, 4	TCGA, In-house dataset: Sanger sequencing	In-house (single center), public (TCGA)	T1CE, T2-FLAIR	Manual	Hybrid (Vision transformer, Radiomics)	Externally validated only	IDH: 85.9%
Wu et al [30]	2776	IDH, 1p/19q	2, 3, 4	TCGA, UCSF, EGD; In-house dataset: IDH: Sanger sequencing; 1p19q: FISH	In-house (multi-center), public (TCGA, UCSF, EGD)	T1CE, T2-FLAIR	mmFormer	3D ResNet-10	Both internally and externally validated	IDH: 85.6%, 1p/19q: 78.8%
Chen et al [94]	1806	IDH, 1p/19q	2, 3, 4	BraTS 2020, EGD, LGG-1p/19q, UCSF-PDGM, REMBRANDT; In-house: NR	In-house (single center), public (BraTS 2020, EGD, LGG-1p/19q, UCSF-PDGM, REMBRANDT)	T1CE, T2	Not undertaken (whole MRI volume + whole-brain mask)	CMTLNet	Both internally and externally validated	IDH: 86.8%, 1p/19q: 74.4%

*Total no. pts* total number of patients, *NR* not reported, *FISH* fluorescence in situ hybridization, *IDH* isocitrate dehydrogenase, *DL* deep learning, *AE* autoencoder, *CNN* convolutional neural network, *GNN* graph neural network, *nnUNet* no-new-net U-Net framework, *RNN* recurrent neural network, *VNet* volumetric neural network, *T1* T1-weighted imaging, *T2* T2-weighted imaging, *T1CE* T1-weighted contrast-enhanced imaging with gadolinium, *T2-FLAIR* T2-weighted fluid-attenuated inversion recovery imaging, *DWI* diffusion-weighted imaging, *2D 55-direction HARDI* 2D 55-direction high angular resolution diffusion imaging, *DSC* dynamic susceptibility contrast MR perfusion, *DSC-PWI* dynamic susceptibility-contrast perfusion-weighted imaging, *CEST* chemical-exchange saturation transfer, *MRAGE* magnetization-prepared rapid acquisition gradient-echo, *qBOLD* quantitative blood-oxygen-level-dependent imaging, *SWI* susceptibility-weighted imaging, *TR-SPACE* T2 sampling perfection with application-optimized contrasts using different flip-angle evolutions, *AUC* area under the curve, *TCGA* the Cancer Genome Atlas, *Ivy GAP* Ivy Glioblastoma Atlas Project, *RHUH-GBM* Río Hortega University Hospital Glioblastoma Dataset, *UPenn-GBM* University of Pennsylvania Glioblastoma Dataset, *UCSF-PDGM* University of California San Francisco Preoperative Diffuse Glioma MRI, *EGD* Erasmus Glioma Database, *LGG-1p19qDeletion* LGG-1p19q deletion dataset

* AUC was not available; accuracy is reported instead

In our qualitative analysis, we identified three primary imaging data sources: private (in-house), public, and a combination of both (Fig. 2B). In-house collections accounted for 35% of the data, whereas 26% relied solely on public datasets, especially The Cancer Imaging Archive (TCIA). Approximately 38% of the studies combined both sources for enhanced research robustness and data diversity. Furthermore, around 63% of the studies implemented data augmentation techniques—either conventional methods [12, 31–35] or generative adversarial networks (GANs) [36–38]—to mitigate overfitting and address class imbalance related to genotype distributions (Supplementary Table 5).

The extensive use of public datasets influenced MRI sequence choices, with conventional methods employed in 81% of the studies. The combination of T1, T1CE, T2, and T2-FLAIR sequences was most prevalent, accounting for 40% of cases. Notably, T1CE was the most frequently utilized, appearing in 26% of studies, followed by T2-FLAIR and T2. Advanced imaging techniques, such as diffusion- and perfusion-weighted imaging, were less commonly employed, appearing in 8% [29, 33, 39–44] and 3% [45–47] of studies, respectively, or in 5% [48–50] when used in combination (Fig. 2C).

DL methods, predominantly based on convolutional neural networks (CNNs), were used for tumor segmentation in 43% of the studies, followed by manual segmentation (26%) and semi-automatic methods (12%) (Fig. 2D). Twenty studies did not incorporate precisely delineated tumors into their prediction models; among these, 55% relied on whole preprocessed MRI slices, 30% utilized regions of interest (ROIs) confined to bounding boxes, and 15% used cropped tumor-bearing regions. Feature extraction primarily relied on CNN-based models, such as ResNet [35, 43, 51–57], DenseNet [32, 58–60], and Inception [61, 62], which were employed in more than half of the studies. Hybrid CNN–radiomics approaches [63–67] and transformer-based models [68–75] followed, appearing in approximately 12% and 7% of cases, respectively. Less commonly used methods included hybrid DL models [36, 41, 64, 65, 76–86], CAE [87, 88], graph neural networks (GNNs) [89], and recurrent neural networks (RNNs) [45] (Fig. 2E). More details on DL model architectures are provided in Supplementary Table 5.

Our review highlights the rise in DL-based radiomics research since 2016 [90]. Initially, CNNs dominated exclusively, making up 100% of methodologies from 2016 to 2018. However, diversification has since increased. By 2020, CNNs still led at 60%, with CAEs [87] and hybrid models combining CNNs with attention mechanisms [36] emerging as alternative models. Transformers, introduced in recent years, peaked at 25% in 2024 (Fig. 2G), indicating a shift to more complex architectures. These networks were primarily integrated into prediction frameworks in an end-to-end manner [33, 41, 51, 52, 62, 71–73,75, 77, 82, 84, 87, 91–94]. In some cases, DL was specifically applied for tumor segmentation [95–97], image preprocessing [98], or classification [48, 99] within the radiomics pipeline.

Pretrained models were used in approximately 38% of the studies (Supplementary Table 5). Among these, 75% fine-tuned the pretrained weights on their own training data, while the remaining studies applied them “as-is,” primarily for tumor segmentation. Notably, several studies that did not fine-tune the segmentation models incorporated expert review and manual correction of the automatically generated ROIs to ensure accuracy [29, 35, 44, 81, 96, 100]. Additionally, clinical parameters, mainly age and sex, were incorporated in 23% of the studies [12, 28, 31, 33, 42, 43, 51, 52, 59, 63, 67, 70, 96, 101–105] (Supplementary Table 5). Regarding model development and evaluation, 37 studies performed external validation, while 65 studies relied solely on internal validation. Figure 2F summarizes the internal validation strategies, highlighting the predominance of the held-out test set approach, followed by K-fold cross-validation.

### Quality assessment

The median RQS was 15 (41.67%), ranging from 7 (19.44%) to 22 (61.11%) out of 36. In Domain 1 (mean score: 2.47 ± 0.92), most studies reported image protocols, but none included multiple time points or phantom studies; however, 71 studies conducted multiple segmentations. Domain 2 scored the highest (mean score: 5.65 ± 1.60), with 35% of studies validating their findings on external datasets. In Domain 3 (mean score: 2.60 ± 0.76), 29% of studies included multivariable analyses incorporating non-radiomic features, and 22% explored biological correlates. Domain 4 had a mean score of 2.74 ± 0.61, with most studies conducting statistical analysis. More than half of the studies used resampling techniques, though only three reported calibration statistics. All studies were retrospective and lacked prospective validation or cost-effectiveness analysis. For Domain 6 (average score: 1.51 ± 1.12), 64% of the studies used open-source data, but only 22% made their code available (Fig. 2H and Supplementary Section 4).

According to the QUADAS-2, the overall risk of bias was high in 48 studies and low in 53 studies, mainly due to limited segmentation methods or the lack of resampling techniques to mitigate overfitting. Additionally, 55 studies raised applicability concerns primarily due to a lack of validation on external datasets (Fig. 2I, J and Supplementary Section 3).

### Publication bias and statistical power

Funnel plot asymmetry and Egger’s test indicated potential publication bias in IDH studies for both internal validation and test sets (*p* < 0.05), whereas no significant bias was detected in 1p/19q studies (*p* > 0.05). To account for the potential bias in IDH prediction, we applied the Trim and Fill method by Duval and Tweedie to adjust the pooled estimates of sensitivity and specificity (Supplementary Sections 7 and 9). The statistical power analysis revealed a high detection capability for larger effect sizes in most included studies but relatively lower power for detecting smaller sensitivity and specificity measures (< 0.3) in some studies [49, 87, 106, 107] (Supplementary Section 11).

### IDH mutation

Most models primarily targeted IDH mutation, either alone in 60% or alongside 1p/19q prediction in 36% of the studies. Over 60% of the studies focused on Grades 2, 3, and 4 gliomas. Grade 4 gliomas were exclusively studied in 12% of the experiments, while Grade 2 gliomas were addressed in only four studies. In the meta-analysis, 75% of studies used DL–based features and 25% relied on conventional radiomics; among the latter, 10 studies applied DL solely for tumor segmentation and 3 for classification.

#### Meta-analysis

In both the internal validation and test cohorts, there was no significant correlation between sensitivity and specificity for IDH prediction, as indicated by SCC of 0.04 (95% CI: −0.27 to 0.33) for sensitivity and 0.01 (95% CI: −0.26 to 0.28) for specificity. In the test cohorts, the bivariate model estimated a pooled sensitivity of 80.4% (95% CI: 77.5–83.0%) and specificity of 84.6% (95% CI: 81.1–87.5%), with 95% prediction intervals ranging from 0.62 to 0.92 for sensitivity and 0.55 to 0.96 for specificity. Although unadjusted heterogeneity was moderate (*I*^2^ = 38.1–69.4%, *p* < 0.001), it was markedly reduced to 2.9–3.5% after adjusting for sample size. Similar performance was achieved for internal validation cohorts (Table 2 and Supplementary Figs. 13 and 15). These results are illustrated in the SROC curves (Fig. 4A, B), which demonstrate strong overall diagnostic performance, with an area under the curve (AUC) of 0.88 for test cohorts and 0.93 for internal validation cohorts. Separate analyses of studies employing DL-based and conventional radiomic features are provided in Table 2. Additionally, forest plots with pooled estimates, including original and imputed studies using the Duval & Tweedie Trim-and-Fill method, are presented in Supplementary Section 9. Sensitivity analyses are reported in Supplementary Section 8.

**Table 2:** Diagnostic performance of MRI-DL models for predicting IDH mutation and 1p/19q codeletion, stratified by studies using DL for feature extraction, radiomic analysis, and all studies combined

	Gene	Dataset	No. of studies	No. of patients	Sensitivity (95% CI)	Specificity (95% CI)	AUC	*I*² (Holling unadj.)	*I*² (Holling adj.)	*p*-value (SEN)	*p*-value (SPE)
DL	IDH	InV	39	7467	0.86 (0.83–0.88)	0.89 (0.87–0.91)	0.93	48.7–74.9%	2.8–4.0%	1.18 × 10^−14^	< 2 × 10^−16^
		Test	39	7284	0.80 (0.76–0.83)	0.86 (0.82–0.89)	0.89	49.2–77.3%	3.5–4.4%	< 2 × 10^−16^	< 2 × 10^−16^
	1p/19q	InV	14	2695	0.83 (0.74–0.89)	0.89 (0.85–0.92)	0.93	60.8–82.4%	4.2–8.0%	3.36e-13	2.88e-10
		Test	13	1599	0.77 (0.66–0.85)	0.84 (0.76–0.89)	0.87	49.9–72.2%	5.0–6.0%	3.42 × 10^−8^	< 2 × 10^−16^
Radiomic	IDH	InV	8	1452	0.85 (0.79–0.89)	0.77 (0.69–0.84)	0.88	30.4–55.7%	1.3–1.7%	0.010	4.02e-4
		Test	13	959	0.79 (0.73–0.83)	0.80 (0.71–0.86)	0.82	0–0%	0–0%	0.86	1.14 × 10^−8^
	1p/19q	InV	0	0	–	–	–	–	–	–	–
		Test^a^	2	16	0–83%	73.3–84%	0.71–0.94	–	–	–	–
All studies	IDH	InV	43^†^	8133	0.86 (0.83–0.88)	0.88 (0.86–0.90)	0.93	52.5–74.0%	2.9–3.9%	6.92e-15	< 2e-16
		Test	52	8243	0.80 (0.77–0.83)	0.85 (0.81–0.87)	0.88	38.1–69.4%	2.9–3.5%	3.27e-11	< 2e-16
	1p/19q	InV	14	2695	0.83 (0.74–0.89)	0.89 (0.85–0.92)	0.93	60.8–82.4%	4.2–8.0%	3.36e-13	2.88e-10
		Test	15	1615	0.75 (0.65–0.82)	0.82 (0.75–0.88)	0.85	45.9–67.5%	5.0–5.8%	3.18e-07	< 2e-16

For each subgroup, the table reports the number of studies, total patients, pooled sensitivity and specificity (95% CI), AUC, heterogeneity (*I*² for Holling unadjusted and adjusted), and *p*-value for both internal validation and test datasets

*No. of studies* number of studies, *No. of patients* number of patients, *IDH* isocitrate dehydrogenase, *CI* confidence interval, *DL* deep learning, *AUC* area under the curve, *InV* internal validation, *SEN* sensitivity, *SPE* specificity, *unadj.* unadjusted, *adj.* adjusted

^a^ For 1p/19q codeletion studies in the test cohorts, only two studies [29, 106] reported radiomics results; therefore, their respective validation ranges are presented instead of pooled estimates

^†^ Some studies included both DL and radiomics models. These were analyzed separately in the DL and Radiomics categories, but only the DL models were included in the All studies analysis

#### Subgroup analysis

We restricted the subgroup analysis to test cohorts (Table 3). Except for the segmentation method and the level of DL integration within the radiomics pipeline, none of the between-group differences reached statistical significance. Semi-automatic segmentation yielded the highest sensitivity, followed by DL-based and manual approaches. End-to-end DL pipelines outperformed those using DL only for feature extraction.

**Table 3:** Meta-regression subgroup analysis exploring heterogeneity in IDH mutation prediction within test cohorts

Covariates	Subgroup	No. of studies	No. of patients	Sensitivity (95% CI)	*p*-value	Specificity (95% CI)	*p*-value
Glioma grade	LGG	7	563	0.86 [0.78; 0.91]	0.24	0.79 [0.71; 0.86]	0.39
	HGG	5	344	0.73 [0.49; 0.88]		0.82 [0.71; 0.90]	
	LGG & HGG	43	6620	0.79 [0.75; 0.83]		0.85 [0.80; 0.89]	
Clinical information	Included	16	3136	0.79 [0.71; 0.84]	0.66	0.85 [0.77; 0.90]	0.90
	Not included	55	6819	0.80 [0.77; 0.83]		0.84 [0.81; 0.87]	
Data augmentation	Included	39	7139	0.79 [0.76; 0.82]	0.71	0.85 [0.82; 0.88]	0.60
	Not included	32	2816	0.80 [0.74; 0.85]		0.83 [0.77; 0.88]	
Dataset	In-house	22	1545	0.79 [0.72; 0.85]	0.08	0.81 [0.75; 0.86]	0.37
	Public	10	7680	0.86 [0.81; 0.90]		0.87 [0.68; 0.96]	
	In-house + Public	39	730	0.79 [0.75; 0.82]		0.85 [0.82; 0.88]	
Segmentation method	DL	12	4334	0.80 [0.76; 0.84]	0.04	0.89 [0.84; 0.92]	0.25
	Manual	8	1093	0.77 [0.67; 0.85]		0.83 [0.78; 0.87]	
	Not undertaken	7	2084	0.72 [0.65; 0.77]		0.84 [0.79; 0.89]	
	Semi-automatic	4	584	0.85 [0.74; 0.93]		0.86 [0.81; 0.90]	
Feature extraction	DL	52	8554	0.80 [0.77; 0.83]	0.93	0.85 [0.82; 0.88]	0.43
	Radiomics	16	1097	0.79 [0.69; 0.86]		0.79 [0.68; 0.87]	
	DL + Radiomics	3	304	0.79 [0.71; 0.85]		0.85 [0.71; 0.93]	
DL models	CNN	34	5909	0.79 [0.75; 0.83]	0.93	0.85 [0.82; 0.88]	0.68
	GNN	4	371	0.80 [0.71; 0.87]		0.86 [0.81; 0.89]	
	Transformer	7	1409	0.78 [0.70; 0.85]		0.84 [0.80; 0.87]	
DL Integration	End-to-end	29	5639	0.81 [0.77; 0.85]	0.37	0.88 [0.83; 0.92]	0.03
	Feature extraction	26	3219	0.78 [0.74; 0.83]		0.82 [0.80; 0.84]	
MRI technique	Conventional	62	9441	0.79 [0.77; 0.83]	0.71	0.85 [0.82; 0.87]	0.42
	Advanced	3	108	0.73 [0.20; 0.97]		0.73 [0.09; 0.99]	
	Advanced + Conventional	6	406	0.83 [0.74; 0.90]		0.80 [0.71; 0.87]	
Validation method	Internally Validated Only	35	2433	0.80 [0.77; 0.83]	0.42	0.83 [0.80; 0.86]	0.52
	Both Internally and Externally Validated	35	7339	0.78 [0.74; 0.82]		0.85 [0.80; 0.88]	

The table reports pooled sensitivity and specificity (95% CI) for each subgroup, alongside *p*-values for between-group differences across covariates

*No. of studies* number of studies, *No. of patients* number of patients, *IDH* isocitrate dehydrogenase, *CI* confidence interval, *DL* deep learning, *AUC* area under the curve, *CNNs* convolutional neural networks, *GNN* graph neural network, *HGG* high-grade glioma, *LGG* low-grade glioma

#### 1p/19q Co-deletion

**Fig. 3:**
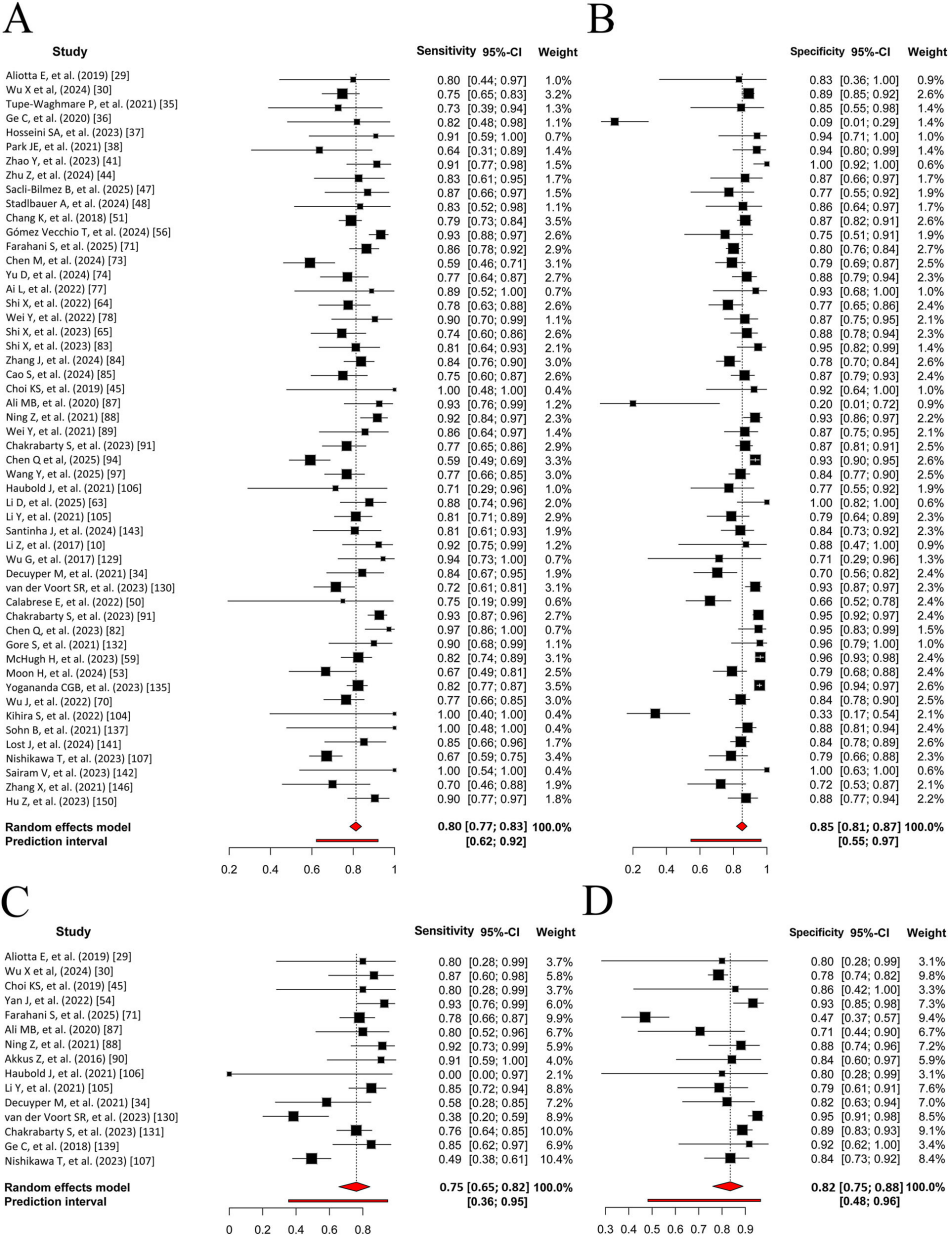
Random forest visualization of test cohorts for molecular marker prediction. **A** Sensitivity for IDH prediction. **B** Specificity for IDH prediction. **C** Sensitivity for 1p/19q prediction. **D** Specificity for 1p/19q prediction. Each plot shows the sensitivity and specificity with 95% CIs and weights for each study. The pooled estimates and prediction intervals under a random effects model are depicted at the bottom of the plots. The numbers represent pooled estimates with 95% CIs in brackets, depicted by horizontal lines. IDH, isocitrate dehydrogenase; CI, confidence interval

**Fig. 4:**
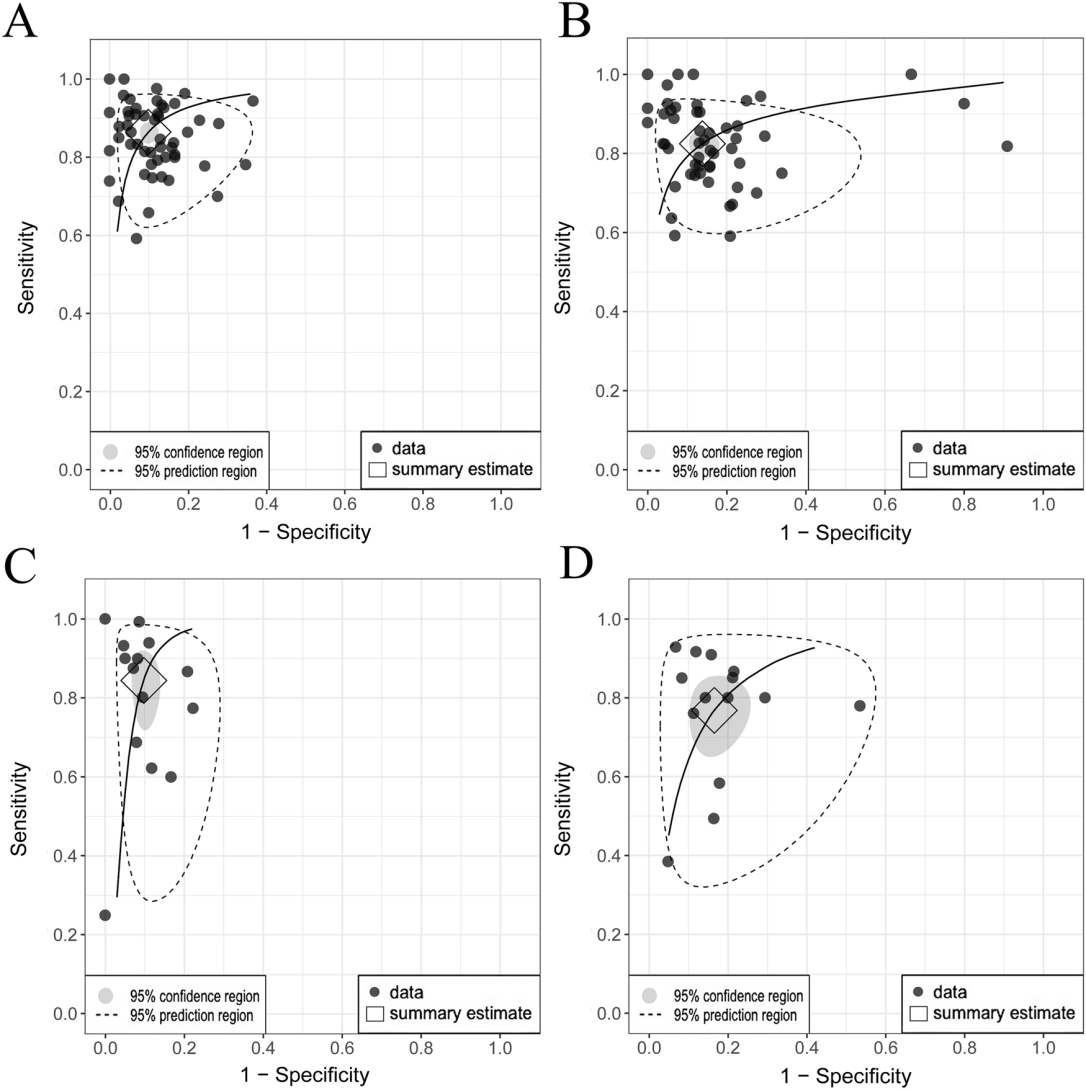
Comparison of SROC curves [26] for IDH and 1p/19q prediction in the internal validation and test cohorts. **A** IDH internal validation: pooled sensitivity 0.86 (95% CI: 0.83–0.88), specificity 0.88 (95% CI: 0.86–0.90), AUC 0.93. **B** IDH test: pooled sensitivity 0.80 (95% CI: 0.77–0.83), specificity 0.85 (95% CI: 0.81–0.87), AUC 0.88. **C** 1p/19q internal validation: pooled sensitivity 0.83 (95% CI: 0.74–0.89), specificity 0.89 (95% CI: 0.85–0.92), AUC 0.93. **D** 1p/19q test: pooled sensitivity 0.75 (95% CI: 0.65–0.82), specificity 0.82 (95% CI: 0.75–0.88), AUC 0.85. Considerable differences between the 95% confidence and prediction regions, particularly for 1p/19q codeletion, highlight significant between-study heterogeneity. SROC, summary receiver operating characteristic; IDH, isocitrate dehydrogenase; AUC, area under the curve; CI, confidence interval

Approximately 5% of the studies focused only on 1p/19q co-deletion, whereas 34% addressed both 1p/19q co-deletion and IDH prediction, mainly in Grades 2 and 3 gliomas. The diagnostic performance of 1p/19q co-deletion in the internal validation and test cohorts (Fig. 3C, D) showed no significant correlation between sensitivity and specificity, with SCCs of 0.08 (95% CI: −0.47 to 0.58) and 0.03 (95% CI: −0.49 to 0.54), respectively. Meta-analysis of test datasets yielded a pooled sensitivity of 74.6% (95% CI: 64.9–82.3%) and specificity of 82.2% (95% CI: 74.8–87.8%) across fourteen experiments. Significant heterogeneity was observed (*I*² = 45.9–67.5%, *p* < 0.001), as shown in the SROC curves (Fig. 4C, D) by the wide, non-overlapping 95% confidence and prediction regions. However, heterogeneity was notably reduced to 5.0–5.8% following sample-size adjustment using the Holling method. Internal validation cohorts demonstrated higher predictive performance (Table 2 and Supplementary Section 9). One study used conventional radiomic features by employing DL solely for image segmentation [29]. Furthermore, sensitivity analyses are detailed in Supplementary Section 8.

#### Subgroup analysis

Due to the limited number of studies per subgroup, meta-regression was not feasible for most covariates. As detailed in Table 4, studies using only in-house datasets demonstrated higher sensitivity but lower specificity compared to those trained and validated on a combination of in-house and public datasets, though the differences were not statistically significant.

**Table 4:** Subgroup meta-regression analysis of heterogeneity in 1p/19q codeletion prediction within test cohorts

Covariates	Subgroup	No. of studies	No. of patients	Sensitivity (95% CI)	*p*-value	Specificity (95% CI)	*p*-value
Data augmentation	Included	10	1038	0.79 [0.68; 0.88]	0.31	0.81 [0.71; 0.88]	0.23
	Not included	5	577	0.68 [0.43; 0.85]		0.88 [0.78; 0.94]	
Dataset	In-house	7	202	0.83 [0.74; 0.89]	0.24	0.80 [0.70; 0.87]	0.08
	In-house + public	7	1245	0.73 [0.53; 0.86]		0.88 [0.82; 0.92]	
Validation method	Internally validated only	7	202	0.83 [0.74; 0.89]	0.10	0.80 [0.70; 0.87]	0.51
	Both Internally and externally validated	7	1347	0.70 [0.53; 0.83]		0.84 [0.71; 0.92]	

Pooled sensitivity and specificity (95% CI) are reported for each subgroup, with *p*-values denoting between-group differences for each covariate

*No. of studies* number of studies, *No. of patients* number of patients, *CI* confidence interval

## Discussion

Our systematic review and meta-analysis critically evaluated the diagnostic performance of MRI-based DL models for predicting IDH mutation and 1p/19q co-deletion in glioma patients. Consistent with prior research [17, 108–110], our findings demonstrate promising overall performance but reveal substantial between-study heterogeneity. Notably, heterogeneity declined markedly after adjusting for sample size, indicating that most of the observed variability in sensitivity and specificity stems from sampling error rather than systematic study differences. Subsequent subgroup analyses confirmed the stability of our pooled estimates, as most examined covariates had no significant effect on model performance. Moreover, our statistical power analysis shows that while some studies had low power for small changes, most were sufficiently powered to detect pooled estimates.

Our meta-regression analysis highlighted tumor segmentation as a major source of variability. Since most features are extracted from defined ROIs, variations in segmentation methods can significantly impact feature reproducibility [111, 112]. We also refined our QUADAS-2 assessment to include an evaluation of segmentation methods, identifying one-third of the studies as unclear or high risk due to inadequate segmentation approaches. To mitigate this, future studies should standardize and streamline the segmentation process. Utilizing robust automated segmentation tools or well-validated semi-automated pipelines can reduce inter-observer variability. Where manual segmentation is unavoidable, having multiple raters and using consensus or average segmentations might improve reliability [113]. Furthermore, several strategies have been proposed to enhance automatic segmentation. For example, applying small dilations and erosions to masks during model training can improve tolerance to boundary shifts [114]. Uncertainty in segmentation can also be estimated using ensemble methods or Monte Carlo dropout [115]. Finally, segmentation-free DL approaches offer a way to bypass manual ROI delineation entirely, potentially avoiding this source of heterogeneity. Adopting these strategies can enhance the reliability of model performance, independent of the segmentation method used.

Studies employing DL in an end-to-end approach outperformed those using DL solely for feature extraction in radiomics workflows. This direct method minimizes potential errors, enhances reproducibility, and improves predictive accuracy. Previous studies have indicated that DL, particularly CNNs, bypasses traditional complexities associated with radiomics workflows, leading to more robust feature extraction [28, 116]. However, our analysis did not reveal any significant differences in predictive performance between radiomic and DL-based features. Importantly, no variation in performance was observed across different DL architectures, including CNNs, GNNs, and transformers. It is worth noting, though, that the limited sample size in some subgroups, such as GNN-based studies with only 304 cases, may affect the reliability of these findings.

Although not statistically significant, consistent trends were observed across both IDH and 1p/19q co-deletion studies regarding data sources. Models trained and validated on the same dataset demonstrated higher pooled sensitivity compared to those using multi-center datasets. In-house datasets, with standardized imaging protocols, typically offer more uniform data quality. In contrast, multi-center datasets introduce greater diversity in scanner vendors, MRI protocols, and patient populations, which can challenge models and lead to apparent performance drops, but ultimately confer more robustness. To reduce scanner- or site-specific biases, various harmonization methods have been developed [117]. At the feature level, techniques like ComBat help align radiomic feature distributions across different scanners by correcting for batch-related effects [118]. On the image level, DL–based approaches such as cycle-consistent generative adversarial networks (CycleGANs) and style transfer can be used to standardize image appearance across datasets [117, 119]. Additionally, fundamental preprocessing steps, such as correcting for bias field inhomogeneity, applying noise reduction filters, and normalizing intensities through methods like z-score scaling or histogram matching, may reduce image heterogeneity at its source [120].

The quality assessments in our systematic review revealed several areas for improvement and current limitations in the field. Consistent with previous reviews [116, 121], the median RQS score of 15 (41.67%) indicates moderate methodological quality, with deficiencies across several domains. Many studies detailed image protocols but lacked multiple time points or phantom studies, reducing reproducibility. Although over 70% of studies evaluated their models on unseen data, nearly half did not use external test datasets. This raises concerns about real-world applicability, as reflected in the RQS and QUADAS-2 assessments. Recent Food and Drug Administration (FDA) guidance on AI-enabled devices highlights that models can inadvertently overfit to features unique to a particular scanner or site [122]. To address this, it is essential to include multi-center training and external validation, and when performance declines across datasets, strategies such as domain adaptation should be employed. Foundation-based DL models offer a promising way forward. Pretrained on large, multi-institutional datasets, they tend to capture more stable and biologically meaningful features, making them more robust to variations in input data [123]. Moreover, to promote fairness across varied populations, models should undergo rigorous testing on diverse subgroups during the development and validation phases. This need is underscored by the fact that fewer than 4% of FDA-approved AI devices report race or ethnicity data [124].

Demonstrating technical performance is only one step; prospective clinical validation under real-world conditions is indispensable to bridging the gap to clinical adoption. In our review, all studies were retrospective. Prospective validation through real-time studies or clinical trials is crucial to show that DL models not only achieve high diagnostic accuracy but also improve patient outcomes compared to standard care. Unlike retrospective studies, prospective validation captures the full clinical workflow—data acquisition, model inference, and clinician decision-making—without hindsight bias, providing a more realistic assessment of the model [125]. Prospective validation also builds the case for regulatory approval and clinical acceptance, as required by related standards such as International Organization for Standardization (ISO) 13485 [126] and International Electrotechnical Commission (IEC) 62304 [127]. For instance, ISO 13485 involves risk management, documentation of design processes, and predefined acceptance criteria for performance. Collaborating with clinical partners to test the model prospectively in a workflow-simulated environment can generate the clinical evidence needed for eventual translation. Finally, incorporating DL into clinical workflows demands compatibility with electronic health records, clinician training, and robust IT infrastructure to support continuous model updates and real-time data integration. It incurs hardware, software, staffing, and maintenance costs that hospitals must weigh against potential benefits [128]. Overcoming these challenges is essential to move DL models from research into practice and advancing personalized oncology care.

This systematic review has several limitations. We focused on top-performing DL models and categorized them broadly due to a scarcity of articles. Nevertheless, we considered variations such as including clinical data, radiomic features, or different MRI sequences within a single study as separate experiments for more detailed analysis. However, these findings are observational rather than causal because randomization did not occur between studies, which is typical in most meta-analyses [22]. There may be other confounding variables influencing these results. Although reconstructing 2 × 2 tables increased the number of studies eligible for meta-analysis, imputation may introduce minor biases. Moreover, we did not assess potential patient overlap across studies. Approximately 26% of included studies relied exclusively on public datasets (mainly TCIA). While this raises the possibility of patient-level overlap, excluding these studies could introduce bias, as model performance on the same dataset can vary considerably depending on the DL framework. Our subgroup analysis further confirmed that the segmentation method and DL integration, rather than dataset origin, were the primary sources of heterogeneity.

In conclusion, our review highlights the promising performance of MRI-based DL models in accurately predicting IDH and 1p/19q co-deletion in glioma patients. To enhance the rigor and facilitate clinical translation of DL models for glioma molecular diagnosis, we propose the following minimum standards identified by our comprehensive analysis: use validated automated or consensus-based segmentation protocols, harmonize multi-center MRI data through methods such as ComBat or DL-based style transfer, incorporate phantom studies to assess feature stability, perform independent external validations without model retraining, and open data and code sharing. The next critical steps are to embed these models in prospective, multi-institutional clinical trials, integrating them into electronic health record workflows, assessing diagnostic accuracy, clinical impact, and cost-effectiveness in real time, and gathering the regulatory evidence needed for safe and effective routine use in neuro-oncology.

## Supplementary information

ELECTRONIC SUPPLEMENTARY MATERIAL

## Abbreviation

AUC: Area under the curve
CAE: Convolutional autoencoder
CI: Confidence interval
CNN: Convolutional neural network
CycleGAN: Cycle-consistent generative adversarial network
DL: Deep learning
DSC: Dynamic susceptibility contrast
FDA: Food and Drug Administration
GNN: Graph neural network
IDH: Isocitrate dehydrogenase
ISO: International Organization for Standardization
RNN: Recurrent neural network
ROC: Receiver operating characteristic
ROI: Region of interest
RQS: Radiomics quality score
SCC: Spearman correlation coefficient
SROC: Summary receiver operating characteristic
TCIA: The cancer imaging archive
