# Supplementary material for: Diagnostic performance of deep learning for predicting glioma isocitrate dehydrogenase and 1p/19q co-deletion in MRI: a systematic review and meta-analysis
Source: Eur Radiol. 2025 Aug 16;36(2):1562–91. doi: 10.1007/s00330-025-11898-2 (PMC12953305; doi:10.1007/s00330-025-11898-2)
Supplement: Supplementary file 1 — ELECTRONIC SUPPLEMENTARY MATERIAL [file 330_2025_11898_MOESM1_ESM.pdf]

Diagnostic Performance of Deep Learning for Predicting Glioma Isocitrate Dehydrogenase and 1p/19q Co-deletion in MRI: A Systematic Review and Meta-Analysis

ELECTRONIC SUPPLEMENTARY MATERIAL

Eur Radiol (2025) Farahani S, Hejazi M, Tabassum M, Di Ieva A, Mahdavifar N, Liu S.

Contents

1. Search Strategy 4
1.2. PubMed 4
1.2. Scopus 5
1.3. Web of Science 6
1.4. Ovid (Embase) 7
1.5. Google Scholar 8
2. Data Extraction Form 9
3. Quality Assessment of Diagnostic Accuracy Studies (QUADAS-2) 10
3.1. QUADAS-2 Questions 10
3.2. QUADAS-2 Results 11
4. Radiomics Quality Score (RQS) 15
4.1. RQS Domains 15
4.2. RQS Results 16
5. Summary of Study Inclusion in Meta-Analysis 18
6. Methodological Characteristics of Included Studies 22
7. Publication Bias 28
7.1. IDH mutation Prediction in Internal Validation Datasets 28
7.1.1. Pooled sensitivity estimate 28
7.2. IDH mutation Prediction in Test Cohorts 32
7.2.1. Pooled sensitivity estimate 32
7.3. 1p/19q Codeletion Prediction in Internal Validation Sets 35
7.3.1. Pooled sensitivity estimate 35
7.3.2. Pooled specificity estimate 36
7.4. 1p/19q Codeletion Prediction in Test Cohorts 38
7.4.1. Pooled sensitivity estimate 38
7.4.2. Pooled specificity estimate 39
8. Sensitivity analysis 40
8.1. IDH mutation Prediction in Internal Validation Datasets 40
8.1.1. Pooled sensitivity estimate 40
8.1.2. Pooled specificity estimate 41
8.2. IDH mutation Prediction in Test Cohorts 42
8.2.1. Pooled sensitivity estimate 42

8.2.2. Pooled specificity estimate 43
8.3. 1p/19q Codeletion Prediction in Internal Validation Datasets 44
8.3.1. Pooled sensitivity estimate 44
8.3.2. Pooled specificity estimate 45
8.4. 1p/19q Codeletion Prediction in Test Cohorts 46
8.4.1. Pooled sensitivity estimate 46
8.4.2. Pooled specificity estimate 47
9. Forest plot 48
9.1. IDH Prediction in Internal Validation Sets 48
9.1.1. Pooled sensitivity estimate (before applying the Duval & Tweedie Trim-and-Fill method) 48
9.1.2. Pooled sensitivity estimate (after applying the Duval & Tweedie Trim-and-Fill method) 49
9.1.3. Pooled specificity estimate (before applying the Duval & Tweedie Trim-and-Fill method) 50
9.1.4. Pooled specificity estimate (after applying the Duval & Tweedie Trim-and-Fill method) 50
9.2. IDH Prediction in Test Cohorts 52
9.2.1. Pooled sensitivity estimate (after applying the Duval & Tweedie Trim-and-Fill method) 52
9.3. 1p/19q Codeletion Prediction in Internal Validation Datasets 53
9.3.1. Pooled sensitivity estimate 53
9.3.3. Pooled specificity estimate (after applying the Duval & Tweedie Trim-and-Fill method) 55
10. Cross-Hair Plot 56
10.1. IDH Prediction in Internal Validation Sets 56
10.2. IDH Prediction in Test Cohorts 57
10.3. 1p/19q Codeletion Prediction in Internal Validation Cohorts 58
10.4. 1p/19q Codeletion Prediction in Test Cohorts 59
11. Statistical Power 59
11.1. IDH Prediction in Internal Validation Datasets 60
11.1.1. Pooled sensitivity estimate 60
11.1.2. Pooled specificity estimate 61
11.2. IDH Prediction in Test Cohorts 62
11.2.1. Pooled sensitivity estimate 62
11.2.2. Pooled specificity estimate 63
11.3. 1p/19q Codeletion Prediction in Internal Validation Sets 64

11.3.1. Pooled sensitivity estimate 64
11.3.2. Pooled specificity estimate 65
11.4. 1p/19q Codeletion Prediction in Test Cohorts 66
11.4.1. Pooled sensitivity estimate 66
11.4.2. Pooled specificity estimate 67
12. Amendment to Registered Protocol 68
13. PRISMA Checklist 69
14. References: 72

1. Search Strategy

(28-3-2025)

1.2. PubMed: [142 results]

("Isocitrate Dehydrogenase"[mesh] OR Isocitrate Dehydrogenase*[tiab] OR "Oligodendroglioma"[mesh] OR "Chromosome Deletion"[mesh] OR "Chromosomes, Human, Pair 1"[mesh] OR "Chromosomes, Human, Pair 19"[mesh] OR "Molecular Diagnostic*"[mesh] OR Oligodendroglioma*[tiab] OR Oligodendroblastoma*[tiab] OR Chromosome Deletion*[tiab] OR Partial Monosom*[tiab] OR Molecular Diagnostic*[tiab] OR Molecular Pathology[tiab] OR Diagnostic Molecular Pathology[tiab] OR Molecular Testing[tiab])
AND
("Artificial Intelligence"[mesh] OR "Generative Artificial Intelligence"[mesh] OR "Deep Learning"[mesh] OR "Detection Algorithms"[mesh] OR "Machine Learning"[mesh] OR "Unsupervised Machine Learning"[mesh] OR "Supervised Machine Learning"[mesh] OR "Reinforcement Machine Learning"[mesh] OR Artificial Intelligence[tiab] OR Computer Reasoning[tiab] OR Machine Intelligence[tiab] OR Computational Intelligence[tiab] OR Computer Vision System*[tiab] OR Generative Artificial Intelligence[tiab] OR Chatbot*[tiab] OR Chat*GPT*[tiab] OR Deep Learning[tiab] OR Hierarchical Learning[tiab] OR Detection Algorithm*[tiab] OR Object Detection Algorithm*[tiab] OR Deep Learning-Based Object Detection Algorithm*[tiab] OR YOLO Network*[tiab] OR Transfer Learning[tiab] OR Federated Machine Learning*[tiab] OR Inductive Machine Learning[tiab] OR Semi*supervised Learning[tiab] OR Reinforcement Machine Learning*[tiab] OR Machine Reinforcement Learning*[tiab] OR Deep Reinforcement Learning*[tiab])
AND
("Glioblastoma"[mesh] OR "Glioma"[mesh] OR "Astrocytoma"[mesh] OR "Brain Neoplasms"[mesh] OR Glioblastoma*[tiab] OR Glioma*[tiab] OR Glial Cell Tumor*[tiab] OR Mixed Glioma*[tiab] OR Astrocytoma*[tiab] OR Astrocytic Glioma*[tiab] OR Astroglioma*[tiab] OR Brain Neoplasm*[tiab] OR Brain Tumor*[tiab] OR Brain Cancer*[tiab] OR Malignant Brain Neoplasm*[tiab] OR Brain Malignant Neoplasm*[tiab] OR Malignant Primary Brain Tumor*[tiab] OR Benign Brain Neoplasm*[tiab] OR Primary Brain Tumor*[tiab] OR Primary Brain Neoplasm*[tiab])
AND
("Magnetic Resonance Imaging"[mesh] OR "Multiparametric Magnetic Resonance Imaging"[mesh] OR "Diffusion Tensor Imaging"[mesh] OR "Diffusion Magnetic Resonance Imaging"[mesh] OR Magnetic Resonance Imaging[tiab] OR NMR Imaging[tiab] OR NMR Tomography[tiab] OR MR Tomography[tiab] OR Proton Spin Tomography[tiab] OR fMRI[tiab] OR Functional MRI*[tiab] OR Functional Magnetic Resonance Imaging[tiab] OR MRI Scan*[tiab] OR Chemical Shift Imaging[tiab] OR Multiparametric Magnetic Resonance Imaging[tiab] OR Multiparametric MRI*[tiab] OR Diffusion Tensor Imaging[tiab] OR DTI MRI[tiab] OR Diffusion Tractography[tiab] OR Diffusion Magnetic Resonance Imaging[tiab] OR Diffusion Weighted MRI*[tiab] OR Diffusion MRI*[tiab])

1.2. **Scopus**: [476 results]

(453 + 23 preprints, Chinese = 6, Russian=1)

TITLE-ABS-KEY (("Isocitrate Dehydrogenase*" OR "isocitric acid dehydrogenase" OR "Oligodendroglioma" OR "anaplastic oligodendroglioma" OR "olegodendrocytoma" OR "olegodendroglioma" OR "oligodendrocyto*" OR "Chromosom* Deletion" OR "Chromosome*" OR "molecular patho*" OR "molecular etiology" OR "Oligodendroglioma*" OR "Oligodendroblastoma*" OR "Anaplastic Oligodendroglioma*" OR "Chromosome Deletion*" OR "Partial Monosom*" OR "Chromosomes, Human, Pair 1" OR "Chromosomes, Human, Pair 19" OR "Chromosome 1" OR "Chromosome 19" OR "Molecular Pathology" OR "Diagnostic Molecular Pathology" OR "Molecular Diagnostic*" OR "Molecular Testing" OR "Chromosome Deletion"))
AND
TITLE-ABS-KEY (("Artificial Intelligence" OR "Computer Reasoning" OR "Machine Intelligence" OR "Computational Intelligence" OR "Computer Vision System*" OR "Generative Artificial Intelligence" OR "Chatbot*" OR "ChatGPT" OR "Deep Learning" OR "Hierarchical Learning" OR "Detection Algorithm*" OR "Object Detection Algorithm*" OR "YOLO Network*" OR "Machine Learning" OR "Transfer Learning" OR "Federated Learning*" OR "Machine Federated Learning*" OR "Federated Machine Learning*" OR "Unsupervised Machine Learning" OR "Supervised Machine Learning" OR "Inductive Machine Learning" OR "Semi*supervised Learning" OR "Reinforcement Machine Learning*" OR "Machine Reinforcement Learning*" OR "Deep Reinforcement Learning*" OR "creative AI" OR "creative artificial intelligence" OR "generative AI" OR "generative A.I." OR "deep machine learning" OR "deep ML" OR "Detect* Algorithm*" OR "feature detect* algorithm" OR "learning machine*" OR "unsupervised learning" OR "supervised learning" OR "reinforcement machine learning" OR "reinforcement learning algorithm"))
AND
TITLE-ABS-KEY (("Glioblastoma*" OR "Glioma*" OR "Glial Cell Tumor*" OR "Mixed Glioma*" OR "Astrocytoma*" OR "Astrocytic Glioma*" OR "Astroglioma*" OR "Brain Neoplasm*" OR "Brain Tumor*" OR "Brain Cancer*" OR "Malignant Brain Neoplasm*" OR "Brain Malignant Neoplasm*" OR "Malignant Primary Brain Tumor*" OR "Primary Malignant Brain Neoplasm*" OR "Brain Benign Neoplasm*" OR "Benign Brain Neoplasm*" OR "Primary Brain Tumor*" OR "Primary Brain Neoplasm*" OR "anaplastic astrocytoma" OR "astrocytoma, anaplastic" OR "gliobastoma multiforme" OR "glioblastoma multiforme" OR "glyoblastoma" OR "malignant glioma" OR "Glioma" OR "brain glioma" OR "cerebral glioma" OR "ganglioglioma" OR "glia* tumo*r" OR "recurrent glioma" OR "astroglioma" OR "oligoastrocytoma" OR "Astrocytoma" OR "brain supratentorial tumo*r" OR "brain tumo*r diagnosis" OR "encephalophyma" OR "intracerebral tumo*r" OR "intracranial neoplasm"))
AND
TITLE-ABS-KEY (("Magnetic Resonance Imaging" OR "NMR Imaging" OR "NMR Tomography" OR "MR Tomography" OR "Proton Spin Tomography" OR "fMRI" OR "Functional MRI*" OR "Functional Magnetic Resonance Imaging" OR "MRI Scan*" OR "Chemical Shift Imaging" OR "Multiparametric Magnetic Resonance Imaging" OR "MpMRI" OR "Multiparametric MRI*" OR "Diffusion Tensor Imaging" OR "DTI MRI" OR "Diffusion Tractography" OR "Diffusion Magnetic Resonance Imaging" OR "Diffusion Weighted MRI*" OR "Diffusion MRI*" OR "nuclear magnetic resonance imaging" OR "magnetic resonance imag*" OR "magnetization transfer imaging" OR "mr imag*" OR "MRI" OR "NMR imag*" OR "Mp*MRI" OR "Multiparametric magnetic resonance imag" OR "multi*parametric MRI" OR "Diffusion Tensor Imag*" OR "diffusion tensor magnetic resonance imag*" OR "diffusion tensor MRI" OR "diffusion tensor tractography" OR "DTI" OR "DTT" OR "magnetic resonance diffusion tensor imag*" OR "Diffusion Magnetic Resonance Imag*" OR "diffusion weighted magnetic resonance" OR "diffusion weighted nuclear magnetic resonance"))

1.3. **Web of Science**: [352 results]
(337 + 15 preprints)

(TS=("Isocitrate Dehydrogenase*" OR "isocitric acid dehydrogenase" OR "Anaplastic Oligodendroglioma*" OR "oligodendrocytoma" OR "oligodendroglioma*" OR "oligodendrocyto*" OR "Chromosom* Deletion" OR "Chromosome*" OR "molecular patho*" OR "molecular etiology" OR "Oligodendroblastoma*" OR "Chromosome Deletion*" OR "Partial Monosom*" OR "Chromosomes, Human, Pair 1" OR "Chromosomes, Human, Pair 19" OR "Chromosome 1" OR "Chromosome 19" OR "Molecular Pathology" OR "Diagnostic Molecular Pathology" OR "Molecular Diagnostic*" OR "Molecular Testing"))

AND

(TS=("Artificial Intelligence" OR "Computer Reasoning" OR "Machine Intelligence" OR "Computational Intelligence" OR "Computer Vision System*" OR "Generative Artificial Intelligence" OR "Chatbot*" OR "ChatGPT" OR "Deep Learning" OR "Hierarchical Learning" OR "Detection Algorithm*" OR "Object Detection Algorithm*" OR "YOLO Network*" OR "Machine Learning" OR "Transfer Learning" OR "Federated Learning*" OR "Unsupervised Machine Learning" OR "Supervised Machine Learning" OR "Inductive Machine Learning" OR "Semisupervised Learning" OR "Reinforcement Machine Learning" OR "Deep Reinforcement Learning*" OR "creative AI" OR "creative artificial intelligence" OR "generative AI" OR "generative A.I." OR "deep machine learning" OR "deep ML" OR "feature detect* algorithm" OR "learning machine*" OR "unsupervised learning" OR "supervised learning" OR "reinforcement machine learning" OR "reinforcement learning algorithm"))

AND

(TS=("Glioblastoma*" OR "Glioma*" OR "Glial Cell Tumor*" OR "Mixed Glioma*" OR "Astrocytoma*" OR "Astrocytic Glioma*" OR "astroglia*" OR "Brain Neoplasm*" OR "Brain Tumor*" OR "Brain Cancer*" OR "Malignant Brain Neoplasm*" OR "Brain Malignant Neoplasm*" OR "Malignant Primary Brain Tumor*" OR "Primary Malignant Brain Neoplasm*" OR "Brain Benign Neoplasm*" OR "Benign Brain Neoplasm*" OR "Primary Brain Tumor*" OR "Primary Brain Neoplasm*" OR "anaplastic astrocytoma" OR "astrocytoma, anaplastic" OR "gliobastoma multiforme" OR "ganglioglioma" OR "glia* tumor" OR "recurrent glioma" OR "oligoastrocytoma" OR "brain supratentorial tumor" OR "brain tumor diagnosis" OR "encephalophy" OR "intracerebral tumor" OR "intracranial neoplasm"))

AND

(TS=("Magnetic Resonance Imaging" OR "NMR Imaging" OR "NMR Tomography" OR "MR Tomography" OR "Proton Spin Tomography" OR "fMRI" OR "Functional MRI*" OR "Functional Magnetic Resonance Imaging" OR "MRI Scan*" OR "Chemical Shift Imaging" OR "Multiparametric Magnetic Resonance Imaging" OR "memri" OR "Multiparametric MRI*" OR "Diffusion Tensor Imaging" OR "DTI MRI" OR "Diffusion Tractography" OR "Diffusion Magnetic Resonance Imaging" OR "Diffusion Weighted MRI*" OR "Diffusion MRI*" OR "nuclear magnetic resonance imaging" OR "magnetic resonance imag*" OR "magnetization transfer imaging" OR "mr imag*" OR "MRI" OR "NMR imag*" OR "MpMRI" OR "Multiparametric magnetic resonance imag" OR "multiparametric MRI" OR "Diffusion Tensor Imag*" OR "diffusion tensor magnetic resonance imag*" OR "diffusion tensor MRI" OR "diffusion tensor tractography" OR "DTI" OR "DTT" OR "magnetic resonance diffusion tensor imag*" OR "Diffusion Magnetic Resonance Imag*" OR "diffusion weighted magnetic resonance" OR "diffusion weighted nuclear magnetic resonance"))

1.4. Ovid (Embase): [858 results]

(Genetic Marker/ OR Biological Marker/ OR Imaging Genomics/ OR Radiomics/ OR Radiogenomics/ OR Isocitrate Dehydrogenase/ OR Chromosome Deletion/ OR Molecular Pathology/ OR Molecular Diagnosis/ OR ("genetic marker*" OR "biological marker*" OR "biomarker*" OR "Imaging Genomics" OR "radiomics" OR "imaging radiogenomics" OR "neuroimaging genomics" OR "radiation genomics" OR "radio*genomics" OR "isocitrate dehydrogenase" OR "isocitric acid dehydrogenase" OR "chromosome deletion" OR "molecular pathology" OR "molecular etiology" OR "molecular pathogenesis" OR "molecular pathophysiology" OR "molecular diagnos*").ti,ab)

AND
(Artificial Intelligence/ OR Generative Artificial Intelligence/ OR Deep Learning/ OR Detection Algorithm/ OR Machine Learning/ OR Federated Learning/ OR Unsupervised Machine Learning/ OR Supervised Machine Learning/ OR Artificial Neural Network/ OR ("artificial intelligence" OR "machine intelligence" OR "generative artificial intelligence" OR "creative AI" OR "creative artificial intelligence" OR "gen*AI" OR "generative A.I." OR "generative AI" OR "deep learning" OR "deep machine learning" OR "deep ML" OR "detection algorithm" OR "detecting algorithm" OR "feature detection algorithm" OR "feature detective algorithm" OR "feature detector algorithm" OR "machine learning" OR "learning machine*" OR "federated learning" OR "unsupervised machine learning" OR "unsupervised learning (machine learning)" OR "supervised machine learning" OR "supervised learning (machine learning)" OR "reinforcement learning algorithm" OR "reinforcement machine learning" OR "artificial neural network*" OR "algorithmic neural network" OR "ANN (artificial neural network)" OR "computational neural network" OR "computer neural network*" OR "mathematical neural network" OR "neural network algorithm").ti,ab)
AND
(Glioblastoma/ OR Glioma/ OR Astrocytoma/ OR ("glioblastoma*" OR "anaplastic astrocytoma" OR "glyoblastoma" OR "malignant glioma" OR "glioma" OR "brain glioma" OR "cerebral glioma" OR "ganglioglioma" OR "glia tumor" OR "glia tumour" OR "glial tumor" OR "glial tumour" OR "high grade glioma" OR "low grade glioma" OR "recurrent glioma" OR "astroglioma" OR "oligoastrocytoma").ti,ab)
AND
(Nuclear Magnetic Resonance Imaging/ OR Multiparametric Magnetic Resonance Imaging/ OR Perfusion Weighted Imaging/ OR Diffusion Weighted Imaging/ OR ("nuclear magnetic resonance imaging" OR "magnetic resonance imaging" OR "magnetic resonance tomography" OR "magnetization transfer imaging" OR "mr imaging" OR "MRI" OR "NMR imaging" OR "Multi*parametric magnetic resonance imaging" OR "mpMRI" OR "multi*parametric MRI" OR "perfusion weighted imaging" OR "perfusion magnetic resonance imaging" OR "perfusion MRI" OR "perfusion nuclear magnetic resonance imaging" OR "perfusion weighted magnetic resonance" OR "perfusion weighted MRI" OR "perfusion weighted nuclear magnetic resonance imaging" OR "diffusion weighted imaging" OR "diffusion magnetic resonance imaging" OR "diffusion MRI" OR "diffusion weighted magnetic resonance" OR "diffusion weighted MRI" OR "diffusion weighted nuclear magnetic resonance").ti,ab)

1.5. Google Scholar: [Top 300 publications sorted by relevance]

("Isocitrate Dehydrogenase*" OR "1p/19q co*deletion")
AND
("Artificial Intelligence" OR "Computer Reasoning" OR "Machine Intelligence" OR "Computational Intelligence" OR "Computer Vision System*" OR "Generative Artificial Intelligence" OR "Chatbot*" OR "ChatGPT" OR "Deep Learning" OR "Hierarchical Learning" OR "Detection Algorithm*" OR "Object Detection Algorithm*" OR "YOLO Network*" OR "Machine Learning" OR "Transfer Learning" OR "Federated Learning*" OR "Machine Federated Learning*" OR "Federated Machine Learning*" OR "Unsupervised Machine Learning" OR "Supervised Machine Learning" OR "Inductive Machine Learning" OR "Semi*supervised Learning" OR "Reinforcement Machine Learning*" OR "Machine Reinforcement Learning*" OR "Deep Reinforcement Learning*" OR "creative AI" OR "creative artificial intelligence" OR "generative AI" OR "generative A.I." OR "deep machine learning" OR "deep ML" OR "Detect* Algorithm*" OR "feature detect* algorithm" OR "learning machine*" OR "unsupervised learning" OR "supervised learning" OR "reinforcement machine learning" OR "reinforcement learning algorithm")
AND
("Glioblastoma*" OR "Glioma*" OR "Glial Cell Tumor*" OR "Mixed Glioma*" OR "Astrocytoma*" OR "Astrocytic Glioma*" OR "astroglia*" OR "Brain Neoplasm*" OR "Brain Tumor*" OR "Brain Cancer*" OR "Malignant Brain Neoplasm*" OR "Brain Malignant Neoplasm*" OR "Malignant Primary Brain Tumor*" OR "Primary Malignant Brain Neoplasm*" OR "Brain Benign Neoplasm*" OR "Benign Brain Neoplasm*" OR "Primary Brain Tumor*" OR "Primary Brain Neoplasm*" OR "anaplastic astrocytoma" OR "astrocytoma, anaplastic" OR "gliobastoma multiforme" OR "glioblastoma" OR "malignant glioma" OR "Glioma" OR "brain glioma" OR "cerebral glioma" OR "ganglioglioma" OR "glia* tumo*r" OR "recurrent glioma" OR "astroglia" OR "oligoastrocytoma" OR "Astrocytoma" OR "brain supratentorial tumo*r" OR "brain tumo*r diagnosis" OR "encephalophy" OR "intracerebral tumo*r" OR "intracranial neoplasm")
AND
("Magnetic Resonance Imaging" OR "NMR Imaging" OR "NMR Tomography" OR "MR Tomography" OR "Proton Spin Tomography" OR "fMRI" OR "Functional MRI*" OR "Functional Magnetic Resonance Imaging" OR "MRI Scan*" OR "Chemical Shift Imaging" OR "Multiparametric Magnetic Resonance Imaging" OR "memri" OR "Multiparametric MRI*" OR "Diffusion Tensor Imaging" OR "DTI MRI" OR "Diffusion Tractography" OR "Diffusion Magnetic Resonance Imaging" OR "Diffusion Weighted MRI*" OR "Diffusion MRI*" OR "nuclear magnetic resonance imaging" OR "magnetic resonance imag*" OR "magnetization transfer imaging" OR "mr imag*" OR "MRI" OR "NMR imag*" OR "Mp*MRI" OR "Multiparametric magnetic resonance imag" OR "multi*parametric MRI" OR "Diffusion Tensor Imag*" OR "diffusion tensor magnetic resonance imag*" OR "diffusion tensor MRI" OR "diffusion tensor tractography" OR "DTI" OR "DTT" OR "magnetic resonance diffusion tensor imag*" OR "Diffusion Magnetic Resonance Imag*" OR "diffusion weighted magnetic resonance" OR "diffusion weighted nuclear magnetic resonance")

2. Data Extraction Form

Table 1. Template data collection form.

	Source					Eligibility		Methods			Patients											Index test																		
Number	Study ID	Report ID	Sources of data (Journal articles, Conference articles, …)	Year	Country	Eligibility for review	Reason for exclusion	Study design	Dataset (single- or multi-center)	Objective	No. of patients (train/test/external validation)	Clinical data inclusion (age, sex, overall survival, …)	Genes	Reference standard	Glioma Grade	Phantom study on all scanners	Imaging at multiple time points	Image protocol quality	MRI	MRI Techniques (Conventional vs. Advanced)	Number of MRI Sequences	Pre-trained Model	Data Augmentation	Segmentation Methods	Manual or Semi-automatic Segmentation: Expert Count	Feature Extraction Method	Cut-off analyses	Calibration statistics	Model Classification	Level of DL Integration	Internal Validation	External Validation	External Validation: Number of Institutions	Performance Metrics (AUC, ACC, Sensitivity, Specificity, …)	Comparison to “ gold standard”	Detect and discuss biological correlates	Potential clinical utility	Cost-effectiveness analysis	Data availability	Code availability

Abbreviations: AUC for Area Under the Curve, ACC for Accuracy.

3. Quality Assessment of Diagnostic Accuracy Studies (QUADAS-2)

3.1. QUADAS-2 Questions

Risk of bias:

Domain 1: Patient Selection
1. Were imaging acquisition protocols clearly documented for each dataset? (Yes/No/Unclear)
2. If a portion of the dataset was selected, was it done randomly? (Yes/No/Unclear)
3. Did the study avoid inappropriate exclusions? (Yes/No/Unclear)
4. Did the study avoid introducing bias by employing appropriate methods to handle missing data? (Yes/No/Unclear)

Domain 2: Index Test
1. Were multiple segmentations performed for manual or semi-automatic methods, or was deep learning-based segmentation utilized? (Yes/No/Unclear)
2. Was a predetermined threshold applied for model predictions? (Yes/No/Unclear)
3. Are discrimination statistics and their significance reported? (Yes/No/Unclear)

Domain 3: Reference Standard
1. Is the reference standard reliable for classifying the target condition? (Yes/No/Unclear)
2. Were severe genotype class imbalances avoided? (Yes/No/Unclear)

Domain 4: Flow & Timing
1. Did all patient groups (train, test, validation) undergo the reference standard? (Yes/No/Unclear)
2. Was the same reference standard applied to all patients? (Yes/No/Unclear)

Applicability Concerns

Domain 1: Patient Selection
Is there concern regarding patient inclusion matching the review question? (Low/High/Unclear)

Domain 2: Index Test
Was validation conducted on an external dataset? (Yes/No/Unclear)

Domain 3: Reference Standard
Does the target condition as defined by the reference standard align with the review question? (Yes/No/Unclear)

Overall Assessment:

- The study is considered to have a "High" risk of bias if any single domain is rated as high.
- The study is considered to have an "Unclear" risk if two or more domains are rated as unclear.

(**Yes**: Low risk of bias, **No**: High risk of bias, **Unclear**: Some concerns)

3.2. QUADAS-2 Results

Table 2. QUADAS-2 assessment results.

	Risk of Bias					Applicability Concerns			
**Study**	D1	D2	D3	D4	**Overall**	D1	D2	D3	**Overall**
Choi KS, et al. (2019) [1]	✓	✓	✓	✓	✓	✓	✗	✓	✗
Fukuma R, et al. (2019) [2]	✗	✗	✓	✓	✗	✓	!	✓	✓
Ge C, et al. (2020) [3]	✓	✓	✓	✓	✓	✓	✗	✓	✗
Li Z, et al. (2017) [4]	✓	✓	✓	✓	✓	!	✗	✓	✗
Liang S, et al. (2018) [5]	✓	✓	✓	✓	✓	✓	✗	✓	✗
Wu G, et al. (2017) [6]	✓	✓	✓	✓	✓	✓	✗	✓	✗
Kim D, et al. (2019) [7]	✓	!	✓	✓	✓	✓	✗	✓	✗
Chang P, et al. (2018) [8]	✓	✓	✓	✓	✓	✓	✗	✓	✗
Ali MB, et al. (2020) [9]	✗	✓	✓	✓	✗	✓	!	✓	✓
Tang Z, et al. (2020) [10]	✓	✓	✓	✓	✓	!	✗	✓	✗
Decuyper M, et al. (2021) [11]	✓	✗	✓	✓	✗	✓	✓	✓	✓
Ning Z, et al. (2021) [12]	✓	✗	✓	!	✗	✓	✓	✓	✓
van der Voort SR, et al. (2023) [13]	✓	✓	✓	!	✓	✓	✓	✓	✓
Cluceru J, et al. (2022) [14]	✓	✗	✓	✓	✗	✓	✓	✓	✓
Haubold J, et al. (2021) [15]	✗	✗	✓	✓	✗	✓	✗	✓	✗
Tupe-Waghmare P, et al. (2021) [16]	!	✗	✓	!	✗	✗	!	✓	✗
Matsui Y, et al. (2020) [17]	✗	✓	✓	✓	✗	✓	✗	✓	✗
Chang K, et al. (2018) [18]	✓	!	✓	✓	✓	✓	!	✓	✓
Calabrese E, et al. (2020) [19]	✓	✓	✓	✓	✓	!	✓	✓	✓
Ai L, et al. (2022) [20]	✓	✓	✓	✓	✓	✓	✗	✓	✗
Chaddad A, et al. (2023) [21]	✓	✓	✓	✓	✓	✓	✗	✓	✗
Chakrabarty S, et al. (2023) [22]	✓	✓	✓	✓	✓	✓	✓	✓	✓
Chakrabarty S, et al. (2023) [23]	✓	✓	✓	!	✓	✓	✓	✓	✓
Chen Q, et al. (2023) [24]	✓	✗	✓	!	✗	✓	✓	✓	✓
Chu W, et al. (2023) [25]	✓	✓	✓	✓	✓	!	✗	✓	✗
Buz-Yalug B, et al. (2024) [26]	✓	✓	✓	✓	✓	✓	✗	✓	✗
Calabrese E, et al. (2022) [27]	✓	✓	✓	✓	✓	!	✗	✓	✗
Cheng J, et al. (2022) [28]	✓	✓	✓	✓	✓	✓	✗	✓	✗
Choi Y, et al. (2020) [29]	✓	✓	✓	✓	✓	✓	✓	✓	✓
Choi YS, et al. (2021) [30]	✓	✓	✓	!	✓	✓	✓	✓	✓
Gore S, et al. (2021) [31]	✓	!	✓	✓	✓	✓	✗	✓	✗
Karami G, et al. (2023) [32]	✓	✓	✓	✓	✓	✓	✗	✓	✗
Liu J, et al. (2024) [33]	✓	✗	!	✓	✗	✓	✗	✓	✗

Table 2. Continued.

	Risk of Bias					Applicability Concerns			
**Study**	D1	D2	D3	D4	**Overall**	D1	D2	D3	**Overall**
McHugh H, et al. (2023) [34]	✓	✗	✓	✓	✗	✓	✓	✓	✓
Moon H, et al. (2024) [35]	✓	✓	✓	!	✓	✓	✓	✓	✓
Nalawade S, et al. (2019) [36]	✓	✓	✓	✓	✓	✓	✗	✓	✗
Nalawade SS, et al. (2022) [37]	✓	✓	✓	✓	✓	✓	✗	✓	✗
Pasquini L, et al. (2021) [38]	✓	✓	✓	✓	✓	✓	✗	✓	✗
Rui W, et al. (2023) [39]	✓	✗	✓	✓	✗	!	✗	✓	✗
Safari M, et al. (2022) [40]	✓	✓	✓	✓	✓	!	✗	✓	✗
Zhang J, et al. (2024) [41]	✓	✓	✓	✓	✓	✓	✓	✓	✓
Zhang H, et al. (2023) [42]	✓	✓	✓	✓	✓	✓	!	✓	✓
Yogananda CGB, et al. (2023) [43]	✓	✓	✓	✓	✓	✓	✓	✓	✓
Zeng H, et al. (2022) [44]	✓	✓	✓	!	✓	!	!	✓	!
Xu Q, et al. (2022) [45]	✗	✓	✓	✓	✗	✓	✗	✓	✗
Wu J, et al. (2022) [46]	✓	✗	✓	✓	✗	✓	✓	✓	✓
Wei Y, et al. (2021) [47]	✓	✓	✓	!	✓	✓	!	✓	✓
Wang Y, et al. (2021) [48]	✓	✓	✓	✓	✓	✓	✗	✓	✗
Wei Y, et al. (2022) [49]	✓	✗	✓	!	✗	✓	!	✓	✓
Wei Y, et al. (2023) [50]	✓	✗	✓	!	✗	✓	!	✓	✓
Tripathi PC, et al. (2023) [51]	✓	✓	✓	✓	✓	✓	!	✓	✓
Shi X, et al. (2023) [52]	✗	✗	✓	✓	✗	✓	✗	✓	✗
Shi X, et al. (2023) [53]	✓	✗	✓	✓	✗	✓	✗	✓	✗
Yan J, et al. (2022) [54]	✓	✗	✓	✓	✗	✓	✓	✓	✓
Kihira S, et al. (2022) [55]	✓	✗	✓	✓	✗	✓	✓	✓	✓
Sohn B, et al. (2021) [56]	✓	✗	✓	✓	✗	✓	✗	✓	✗
Buda M, et al. (2019) [57]	✓	!	✓	✓	✓	✓	✗	✓	✗
Ali MB, et al. (2023) [58]	✓	!	✓	!	!	✓	!	✓	✓
Chen M, et al. (2024) [59]	✓	!	✓	!	!	✓	✓	✓	✓
Elyassirad D, et al. (2024) [60]	✓	✓	✓	✓	✓	✓	✗	✓	✗
Fayyaz M, et al. (2023) [61]	✓	✗	✓	✓	✗	✓	✗	✓	✗
Ge C, et al. (2018) [62]	✗	!	✓	✓	✗	✓	✗	✓	✗
Gómez Vecchio T, et al. (2024) [63]	✓	✓	!	!	!	✓	✓	✓	✓
Hosseini SA, et al. (2023) [64]	✓	✗	✓	✓	✗	✓	✗	✓	✗
Jeon YH, et al. (2025) [65]	✓	✗	!	✓	✗	✓	✗	✓	✗

Table 2. Continued.

	Risk of Bias					Applicability Concerns			
**Study**	D1	D2	D3	D4	**Overall**	D1	D2	D3	**Overall**
Jian J, et al. (2025) [66]	✓	✗	✓	✓	✗	✓	✗	✓	✗
Li D, et al. (2025) [67]	✓	✗	!	✓	✗	✓	✓	✓	✓
Li X, et al. (2021) [68]	✗	✓	✓	✓	✗	✓	✗	✓	✗
Li Y, et al. (2021) [69]	✓	✓	✓	✓	✓	✓	✗	✓	✗
Lost J, et al. (2024) [70]	✓	✓	✓	✓	✓	✓	✓	✓	✓
Nishikawa T, et al. (2023) [71]	✓	✓	✓	✓	✓	✓	✓	✓	✓
Park JE, et al. (2021) [72]	✓	✗	✓	✓	✗	✓	✗	✓	✗
Sacli-Bilmez B, et al. (2025) [73]	✓	✗	✓	✓	✗	✓	✗	✓	✗
Sairam VA, et al. (2023) [74]	✓	✗	✓	✓	✗	!	✗	✓	✗
Santinha J, et al. (2024) [75]	✓	✗	✓	!	✗	✓	✓	✓	✓
Shi X, et al. (2022) [76]	✗	✗	✓	✓	✗	✓	✗	✓	✗
Stadlbauer A, et al. (2024) [77]	✓	✗	✓	✓	✗	✓	✓	✓	✓
Taha B, et al. (2021) [78]	✓	✗	!	!	✗	!	✓	✓	✓
Usuzaki T, et al. (2024) [79]	!	✗	✓	✓	✗	!	✓	✓	✓
Wang Y, et al. (2025) [80]	✓	✓	✓	✓	✓	✓	✓	✓	✓
Wankhede DS, et al. (2022) [81]	✗	!	✓	✓	✗	!	✗	✓	✗
Yang Z, et al. (2025) [82]	✓	✗	✓	!	✗	✓	✓	✓	✓
Yu D, et al. (2024) [83]	✓	✓	✓	✓	✓	✓	✓	✓	✓
Yuan Y, et al. (2024) [84]	✓	✓	✓	✓	✓	✓	✗	✓	✗
Yuan Y, et al. (2023) [85]	✓	✗	✓	✓	✗	✓	✗	✓	✗
Zhang S, et al. (2021) [86]	✓	✓	✓	✓	✓	✓	✗	✓	✗
Zhang X, et al. (2021) [87]	✗	✗	✓	✓	✗	✓	✗	✓	✗
Zhao K, et al. (2023) [88]	✓	✓	✓	!	✓	✓	!	✓	✓
Zhu Z, et al. (2024) [89]	✓	✗	✓	✓	✗	✓	✓	✓	✓
Aliotta E, et al. (2019) [90]	✓	✗	✓	✓	✗	✓	✗	✓	✗
Alom Z, et al. (2023) [91]	✓	✓	✓	✓	✓	✓	✗	✓	✗
González SR, et al. (2019) [92]	✓	✗	✓	✓	✗	✓	✗	✓	✗
Riahi Samani Z, et al. (2023) [93]	✓	✗	✓	✓	✗	✓	✗	✓	✗
Sun X, et al. (2024) [94]	✓	✓	✓	!	✓	✓	!	✓	✓
Zhao Y, et al. (2023) [95]	✓	✗	✓	!	✗	✓	✓	✓	✓
Zhang L, et al. (2024) [96]	✓	✗	✓	!	✗	✓	✓	✓	✓
Yogananda CG, et al. (2020) [97]	✓	✓	✓	✓	✓	✓	✗	✓	✗
Akkus Z, et al. (2016) [98]	✓	✗	✓	✓	✗	✓	✗	✓	✗

Table 2. Continued.

Study	Risk of Bias					Applicability Concerns			
	D1	D2	D3	D4	**Overall**	D1	D2	D3	**Overall**
Cao S, et al. (2024) [99]	✓	✓	✓	✓	✓	✓	✓	✓	✓
Hu et al. (2023) [100]	✓	✗	✓	✓	✗	✓	✗	✓	✗
Farahani S, et al. (2025) [101]	✓	✓	✓	✓	✓	✓	✓	✓	✓
Niu W, et al. (2025) [102]	✓	✓	✓	✓	✓	✓	✓	✓	✓
Wu X et al, 2024 [103]	✓	✓	✓	✓	✓	✓	✓	✓	✓
Chen Q et al, 2025 [104]	✓	✓	✓	✓	✓	✓	✓	✓	✓

4. Radiomics Quality Score (RQS)

4.1. RQS Domains

- **Domain 1**—Protocol quality and stability in image and segmentation (0–5 points)
- **Domain 2**—Feature selection and validation (−8 to 8 points)
- **Domain 3**—Biologic/clinical validation and utility (0–6 points)
- **Domain 4**—Model performance index (0–5 points)
- **Domain 5**—High level of evidence (0–8 points)
- **Domain 6**—Open science and data (0–4 points)

4.2. RQS Results

Table 3. Heatmap of Radiomics Quality Scores (RQSs) for included articles.

Study	Domain 1	Domain 2	Domain 3	Domain 4	Domain 5	Domain 6
Choi KS, et al. (2019) [1]						
Fukuma R, et al. (2019) [2]						
Ge C, et al. (2020) [3]						
Li Z, et al. (2017) [4]						
Liang S, et al. (2018) [5]						
Wu G, et al. (2017) [6]						
Kim D, et al. (2019) [7]						
Chang P, et al. (2018) [8]						
Ali MB, et al. (2020) [9]						
Tang Z, et al. (2020) [10]						
Decuyper M, et al. (2021) [11]						
Ning Z, et al. (2021) [12]						
van der Voort SR, et al. (2023) [13]						
Cluceru J, et al. (2022) [14]						
Haubold J, et al. (2021) [15]						
Tupe-Waghmare P, et al. (2021) [16]						
Matsui Y, et al. (2020) [17]						
Chang K, et al. (2018) [18]						
Calabrese E, et al. (2020) [19]						
Ai L, et al. (2022) [20]						
Chaddad A, et al. (2023) [21]						
Chakrabarty S, et al. (2023) [22]						
Chakrabarty S, et al. (2023) [23]						
Chen Q, et al. (2023) [24]						
Chu W, et al. (2023) [25]						
Buz-Yalug B, et al. (2024) [26]						
Calabrese E, et al. (2022) [27]						
Cheng J, et al. (2022) [28]						
Choi Y, et al. (2020) [29]						
Choi YS, et al. (2021) [30]						
Gore S, et al. (2021) [31]						
Karami G, et al. (2023) [32]						
Liu J, et al. (2024) [33]						
McHugh H, et al. (2023) [34]						
Moon H, et al. (2024) [35]						
Nalawade S, et al. (2019) [36]						
Nalawade SS, et al. (2022) [37]						
Pasquini L, et al. (2021) [38]						
Rui W, et al. (2023) [39]						
Safari M, et al. (2022) [40]						
Zhang J, et al. (2024) [41]						
Zhang H, et al. (2023) [42]						
Yogananda CGB, et al. (2023) [43]						
Zeng H, et al. (2022) [44]						
Xu Q, et al. (2022) [45]						

0

100

Table 3. Continued.

Study	Domain 1	Domain 2	Domain 3	Domain 4	Domain 5	Domain 6
Wu J, et al. (2022) [46]						
Wei Y, et al. (2021) [47]						
Wang Y, et al. (2021) [48]						
Wei Y, et al. (2022) [49]						
Wei Y, et al. (2023) [50]						
Tripathi PC, et al. (2023) [51]						
Shi X, et al. (2023) [52]						
Shi X, et al. (2023) [53]						
Yan J, et al. (2022) [54]						
Kihira S, et al. (2022) [55]						
Sohn B, et al. (2021) [56]						
Buda M, et al. (2019) [57]						
Ali MB, et al. (2023) [58]						
Chen M, et al. (2024) [59]						
Elyassirad D, et al. (2024) [60]						
Fayyaz M, et al. (2023) [61]						
Ge C, et al. (2018) [62]						
Gómez Vecchio T, et al. (2024) [63]						
Hosseini SA, et al. (2023) [64]						
Jeon YH, et al. (2025) [65]						
Jian J, et al. (2025) [66]						
Li D, et al. (2025) [67]						
Li X, et al. (2021) [68]						
Li Y, et al. (2021) [69]						
Lost J, et al. (2024) [70]						
Nishikawa T, et al. (2023) [71]						
Park JE, et al. (2021) [72]						
Sacli-Bilmez B, et al. (2025) [73]						
Sairam VA, et al. (2023) [74]						
Santinha J, et al. (2024) [75]						
Shi X, et al. (2022) [76]						
Stadlbauer A, et al. (2024) [77]						
Taha B, et al. (2021) [78]						
Usuzaki T, et al. (2024) [79]						
Wang Y, et al. (2025) [80]						
Wankhede DS, et al. (2022) [81]						
Yang Z, et al. (2025) [82]						
Yu D, et al. (2024) [83]						
Yuan Y, et al. (2024) [84]						
Yuan Y, et al. (2023) [85]						
Zhang S, et al. (2021) [86]						
Zhang X, et al. (2021) [87]						
Zhao K, et al. (2023) [88]						
Zhu Z, et al. (2024) [89]						

0

100

Table 3. Continued.

Study	Domain 1	Domain 2	Domain 3	Domain 4	Domain 5	Domain 6
Aliotta E, et al. (2019) [90]						
Alom Z, et al. (2023) [91]						
González SR, et al. (2019) [92]						
Riahi Samani Z, et al. (2023) [93]						
Sun X, et al. (2024) [94]						
Zhao Y, et al. (2023) [95]						
Zhang L, et al. (2024) [96]						
Yogananda CG, et al. (2020) [97]						
Akkus Z, et al. (2016) [98]						
Cao S, et al. (2024) [99]						
Hu et al. (2023) [100]						
Farahani S, et al. (2025) [101]						
Niu W, et al. (2025) [102]						
Wu X et al, 2024 [103]						
Chen Q et al, 2025 [104]						

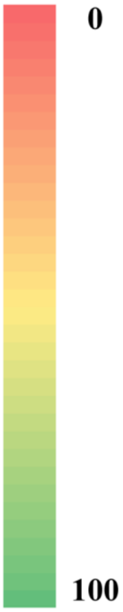

5. Summary of Study Inclusion in Meta-Analysis

Table 4. Summary of inclusion status of each eligible study in the meta-analysis. For each study, inclusion based on internal validation and test set (unseen dataset) is indicated. Studies excluded from the meta-analysis are annotated with numeric codes denoting the reason: (1) experiment not performed in the study; (2) insufficient validation metrics to reconstruct the confusion matrix; (3) inconsistency between reconstructed and reported performance metrics (e.g., accuracy, precision).

Study	IDH included		1p/19q included		Exclusion reason(s)[1]
	Internal validation	Test set	Internal validation	Test set	
Choi KS, et al. (2019) [1]	Yes	Yes	No	No	—
Fukuma R, et al. (2019) [2]	No	No	No	No	2
Ge C, et al. (2020) [3]	No	Yes	No	No	1
Li Z, et al. (2017) [4]	Yes	Yes	No	No	1
Liang S, et al. (2018) [5]	Yes	No	No	No	1
Wu G, et al. (2017) [6]	Yes	Yes	No	No	1
Kim D, et al. (2019) [7]	No	No	No	No	1, 3
Chang P, et al. (2018) [8]	No	No	No	No	1, 2
Ali MB, et al. (2020) [9]	No	Yes	No	No	1, 3
Tang Z, et al. (2020) [10]	No	No	No	No	1, 3
Decuyper M, et al. (2021) [11]	Yes	Yes	Yes	Yes	—
Ning Z, et al. (2021) [12]	No	Yes	No	No	1
van der Voort SR, et al. (2023) [13]	No	Yes	No	No	1
Cluceru J, et al. (2022) [14]	No	No	No	No	1
Haubold J, et al. (2021) [15]	Yes	Yes	Yes	No	3
Tupe-Waghmare P, et al. (2021) [16]	No	Yes	No	No	1, 3
Matsui Y, et al. (2020) [17]	No	No	No	No	1
Chang K, et al. (2018) [18]	No	Yes	No	No	1
Calabrese E, et al. (2020) [19]	Yes	Yes	No	No	1
Ai L, et al. (2022) [20]	No	Yes	No	No	1
Chaddad A, et al. (2023) [21]	No	No	No	No	1
Chakrabarty S, et al. (2023) [22]	Yes	Yes	Yes	Yes	—
Chakrabarty S, et al. (2023) [23]	No	Yes	No	No	1
Chen Q, et al. (2023) [24]	Yes	Yes	No	No	1
Chu W, et al. (2023) [25]	No	No	No	No	1, 2
Buz-Yalug B, et al. (2024) [26]	Yes	No	No	No	1
Calabrese E, et al. (2022) [27]	Yes	No	No	No	1
Cheng J, et al. (2022) [28]	Yes	No	No	No	1
Choi Y, et al. (2020) [29]	No	No	No	No	1, 2
Choi YS, et al. (2021) [30]	No	No	No	No	1, 2
Gore S, et al. (2021) [31]	No	Yes	No	No	1
Karami G, et al. (2023) [32]	No	No	Yes	No	1, 3
Liu J, et al. (2024) [33]	No	No	No	No	1
McHugh H, et al. (2023) [34]	Yes	Yes	No	No	3
Moon H, et al. (2024) [35]	Yes	Yes	No	No	1
Nalawade S, et al. (2019) [36]	Yes	No	No	No	1
Nalawade SS, et al. (2022) [37]	No	No	No	No	1, 2
Pasquini L, et al. (2021) [38]	Yes	No	No	No	1
Rui W, et al. (2023) [39]	Yes	No	No	No	1

Safari M, et al. (2022) [40]	No	No	No	No	1, 2
Zhang J, et al. (2024) [41]	No	Yes	No	No	1, 2
Zhang H, et al. (2023) [42]	Yes	No	No	No	1, 2
Yogananda CGB, et al. (2023) [43]	No	Yes	No	No	1
Zeng H, et al. (2022) [44]	Yes	No	No	No	1
Xu Q, et al. (2022) [45]	Yes	No	No	No	1
Wu J, et al. (2022) [46]	Yes	Yes	No	No	1
Wei Y, et al. (2021) [47]	Yes	Yes	No	No	1
Wang Y, et al. (2021) [48]	Yes	No	No	No	1
Wei Y, et al. (2022) [49]	Yes	Yes	No	No	1
Wei Y, et al. (2023) [50]	No	No	No	No	1, 3
Tripathi PC, et al. (2023) [51]	Yes	No	Yes	No	1
Shi X, et al. (2023) [52]	No	Yes	No	No	1
Shi X, et al. (2023) [53]	No	Yes	No	No	1
Yan J, et al. (2022) [54]	No	No	Yes	Yes	1
Kihira S, et al. (2022) [55]	No	Yes	No	No	1
Sohn B, et al. (2021) [56]	No	Yes	No	No	1
Buda M, et al. (2019) [57]	No	No	No	No	1
Ali MB, et al. (2023) [58]	Yes	No	No	No	1
Chen M, et al. (2024) [59]	Yes	Yes	No	No	1
Elyassirad D, et al. (2024) [60]	No	No	No	No	1, 2
Fayyaz M, et al. (2023) [61]	Yes	No	No	No	1
Ge C, et al. (2018) [62]	No	No	No	Yes	1
Gómez Vecchio T, et al. (2024) [63]	No	Yes	No	No	1
Hosseini SA, et al. (2023) [64]	No	Yes	No	No	1
Jeon YH, et al. (2025) [65]	No	No	No	No	1, 2
Jian J, et al. (2025) [66]	No	No	No	No	1, 2
Li D, et al. (2025) [67]	Yes	Yes	No	No	1
Li X, et al. (2021) [68]	No	No	No	No	1, 2
Li Y, et al. (2021) [69]	Yes	Yes	Yes	Yes	—
Lost J, et al. (2024) [70]	Yes	Yes	No	No	1
Nishikawa T, et al. (2023) [71]	Yes	Yes	Yes	Yes	—
Park JE, et al. (2021) [72]	No	Yes	No	No	1
Sacli-Bilmez B, et al. (2025) [73]	Yes	Yes	No	No	1
Sairam VA, et al. (2023) [74]	No	Yes	No	No	1
Santinha J, et al. (2024) [75]	Yes	Yes	No	No	1
Shi X, et al. (2022) [76]	No	Yes	No	No	1
Stadlbauer A, et al. (2024) [77]	No	Yes	No	No	1, 2
Taha B, et al. (2021) [78]	No	No	No	No	1, 2
Usuzaki T, et al. (2024)* [79]	No	No	No	No	1, 2
Wang Y, et al. (2025) [80]	Yes	Yes	No	No	1
Wankhede DS, et al. (2022) [81]	No	No	No	No	1
Yang Z, et al. (2025) [82]	No	No	No	No	1, 2
Yu D, et al. (2024) [83]	Yes	Yes	No	No	1
Yuan J, et al. (2024) [84]	Yes	No	No	No	1, 3
Yuan Y, et al. (2023) [85]	Yes	No	No	No	1
Zhang S, et al. (2021) [86]	No	No	No	No	1
Zhang X, et al. (2021) [87]	No	Yes	No	No	1

Zhao K, et al. (2023) [88]	No	No	Yes	No	1
Zhu Z, et al. (2024) [89]	Yes	Yes	No	No	1
Aliotta E, et al. (2019) [90]	No	Yes	No	Yes	1
Alom Z, et al. (2023) [91]	No	No	Yes	No	1, 2
González SR, et al. (2019) [92]	No	No	Yes	No	1, 2
Riahi Samani Z, et al. (2023) [93]	No	No	Yes	No	1
Sun X, et al. (2024) [94]	No	No	No	No	1
Zhao Y, et al. (2023) [95]	No	Yes	No	No	1
Zhang L, et al. (2024) [96]	No	No	No	No	1, 2
Yogananda CG, et al. (2020) [97]	No	No	Yes	No	1
Akkus Z, et al. (2016) [98]	No	No	Yes	Yes	1
Cao S, et al. (2024) [99]	Yes	Yes	No	No	3
Hu Z, et al. (2023) [100]	Yes	Yes	No	No	1
Farahani S, et al. (2025) [101]	Yes	Yes	Yes	Yes	—
Niu W, et al. (2025) [102]	No	No	No	No	1, 3
Wu X et al, (2024) [103]	Yes	Yes	Yes	Yes	—
Chen Q et al, (2025) [104]	Yes	Yes	No	No	3

* The study by Usuzaki T, et al. (2024) was excluded since it reported perfect internal validation performance (F1 score, AUC, sensitivity, and specificity all equal to 100%), which may suggest potential overfitting.

6. Methodological Characteristics of Included Studies

Table 5. Overview of included studies, detailing patient numbers in training, internal validation, and external validation groups; deep learning architecture; pretrained weights; demographics; data augmentation; training setup. **Abbreviations**: CV, cross-validation; LOOCV, leave-one-out cross-validation; CNN, convolutional neural network; Bi-LSTM, bidirectional long short-term memory; FFN, feedforward network; GAN, generative adversarial network; SVM, support vector machine; RF, random forest; KPS, Karnofsky Performance Status; NR, not reported; RQS, Radiomics Quality Score

Study	No. of Patients	Model Architecture	Pretrained Weights	Weight Adaptation	Patient Demographics	Data Augmentation	Training Parameters	Tools	RQS (-8 to 36)
Choi KS, et al. (2019) [1]	Training/Internal validation (5-fold CV): IDH: 445, 1p/19q: 113<br>Held-out test: IDH: 18, 1p/19q: 12	1D CNN → Bi-LSTM with attention → FFN	No	NA	No	Sliding window	NR	Python, NordicICE, FSL, 3D Slicer	14
Fukuma R, et al. (2019) [2]	Training/Internal validation (10-fold nested CV): 164	AlexNet (conv5 features) + SVM classifier	Yes	Fine-tuned	Yes (Age)	Cropping, rotation, flipping	SVM parameters optimized internally; CNN NR (network not retrained)	Python, MATLAB, FSL, MRIConvert, Caffe, Chainer	11
Ge C, et al. (2020) [3]	Training/Internal validation: 133<br>Held-out test: IDH: 33	Multi-stream 2D CNN + GAN (U-Net generator, Markov discriminator), bilinear refinement	No	NA	No	GAN-based augmentation; flipping, shifting	GAN: 1 000 epochs, Adam lr=2×10^{-4}; CNN pre-train: 100 epochs Adagrad lr schedule; CNN fine-tune: 50 epochs lr=1×10^{-6}	Python, Keras	15
Li Z, et al. (2017) [4]	Training/Internal validation (LOOCV): 229<br>Temporal split (pre/post 2015) training: 85, held-out test: 34	Modified CNN (6 conv layers, FC-4096) + Fisher vector encoding; radiomics-SVM	No	NA	No	Multi-scale sampling	SGD (details NR); patch size 33×33; dropout after FC layers	Python, SPM, Brainsuite	13
Liang S, et al. (2018) [5]	Training/Internal validation (5-fold CV): 167	Multimodal 3D DenseNet variants (121, 161, 169, 201)	No	NA	No	Flipping, shifting	lr=0.01; momentum=0.9; lr decay 0.1 every 50 epochs; batch size=8	Python, MXNet v1.0	15
Wu G, et al. (2017) [6]	Training/Internal validation (LOOCV): 80<br>Held-out test: 80	Segmentation CNN (6 conv + 2 pool + 3 FC) + KSVD/OMP sparse coding + iterative selection	No	NA	No	No	NR (254 000 patches 33×33; other hyperparams NR)	Python, MATLAB 8.6	13
Kim D, et al. (2019) [7]	Training/Internal validation (5-fold CV): 114<br>Held-out test: IDH: 29	Random Forest, Logistic Regression, kTSP; pretrained CNN	Yes	Fine-tuned	Yes (age, gender, KPS)	Rotations, translations, noise, volume/contour perturbations	RF: 200–2 000 trees, max depth 10–100; LR: L1/L2 regs (10^{-3}–10^{5}); kTSP: k=3–15, 5 000 iterations	Python, Pyradiomics, GUDHI, Switchbox R, scikit-learn	15
Chang P, et al. (2018) [8]	Training/Internal validation (5-fold CV): 259	Custom 2D CNN (multi-conv + 64-d hidden)	No	NA	No	Normalization, resizing	25 k iter (~3 k epochs); bs 12–48; LR NR	FLIRT, Python	16
Ali MB, et al. (2020) [9]	Training/Internal validation (5-fold CV): 129<br>Held-out test: 32	CAE + CycleGAN domain mapping + DCGAN aug	Yes	Fine-tuned	No	DCGAN; horizontal flips, random rotations	CycleGAN: 150 ep, LR 2e-4 (decay); DCGAN: LR 0.002, bs 64; CAE: pretrain 200 ep (LR 0.002, bs 16), then fine-tune w/ early stop	Python, Keras, FSL, ANTs	10
Tang Z, et al. (2020) [10]	Training/Internal validation (10-fold CV): IDH: 93, 1p/19q: 59	Multi-task CNN (shared conv + genotype/OS branches)	No	NA	Yes (age, gender, tumor size, and location)	Rotation, mirroring	bs 3; max 100 k iter (~30 ep); LR NR	Python, PANDA	14
Decuyper M, et al. (2021) [11]	Training: IDH: 210, 1p/19q: 183<br>Internal Validation: IDH: 100, 1p/19q: 54<br>External Validation: IDH: 86, 1p/19q: 40	Two-stage: U-Net seg → 3D CNN class	No	NA	No	Random flip, rotations, intensity scaling; elastic; channel dropout	Segmentation: Adam (LR 1e-4, bs 2); Dice+CE loss, Classification: AdamW (LR 1e-5, bs 8); focal BCE; early stop 30 ep; dropout-0.1	Python, PyTorch, SPM12, MATLAB R2018b	15
Ning Z, et al. (2021) [12]	Training: IDH: 373, 1p/19q: 246<br>Internal Validation: N/A<br>External Validation: IDH: 183, 1p/19q: 66	2.5D patch CAE → view-sharable fusion → classifier	No	NA	No	Multi-view patch generation (axial/coronal/sagittal)	LR 1e-3; momentum 0.9; bs 512 (pretrain)/20 (ft); ep 100/50	Python, Keras, ITK-SNAP	14
van der Voort SR, et al. (2023) [13]	Training: IDH: 666, 1p/19q: 440<br>Held-out test: IDH: 226, 1p/19q: 66<br>External Validation: IDH: 217, 1p/19q: 233	Multi-task 3D CNN with separate branches for segmentation & for classification	No	NA	No	No	Optimal hyperparameters selected via 15% hold-out set (LR, batch size, epochs not specified)	Python, Docker container	19
Cluceru J, et al. (2022) [14]	Training: IDH: 349, 1p/19q: 237<br>Held-out test: IDH: 49, 1p/19q: 38<br>External validation: IDH: 159, 1p/19q: 72	VGG-16 for 3-class end-to-end; ResNet-18 for 1p/19q in 2-tiered model	Yes	Fine-tuned	Yes (age, gender)	No	“One Cycle” LR scheduling; Adam optimizer; weight decay 0.01; batch size & epochs not specified (see Supplementary)	FSL (FLIRT, BET), 3D Slicer, Python, scikit-learn	17

Haubold J, et al. (2021) [15]	Training/Internal validation (bootstrapping): IDH: 116, 1p/19q: 24<br>Held-out test: IDH: 29, 1p/19q: 6	Segmentation: DeepMedic (3D CNN); Classification: XGBoost	Yes	Fine-tuned	No	No	XGBoost hyperparam ranges: n_estimators 100–1500, max_depth 1–6, learning_rate 0.03–0.05, gamma 0–20, min_child_weight 1–20, subsample 0.5–1.0, colsample_bytree 0.1–1.0, reg_lambda 1e-8–1.0; early stopping 10; 1000-fold bootstrapping	Python, PyRadiomics, SimpleITK/SimpleElastix	11
Tupe-Waghmare P, et al. (2021) [16]	Training: IDH: 245, 1p/19q: 122<br>Held-out test: IDH: 24, 1p/19q: 24	Hierarchical multitask: CA (autoencoder) + CNN (ResNet50 backbone) with task-specific branches for IDH and 1p/19q	Yes	Fine-tuned	No	Flipping, rotation, zooming, shearing, shifts	CA: 500 epochs (LR 1×10^{-4}, dice loss); Base CNN: 425 epochs (LR $3\times10^{-3}\rightarrow3\times10^{-7}$); Multilabel models: 300 epochs; batch size NR	Python, FSL BET, ANTs, ITKSNAP	14
Matsui Y, et al. (2020) [17]	Training/Internal validation (LOOCV): IDH: 217, 1p/19q: 117	Residual networks with fully connected layers integrating numeric & imaging inputs	No	NA	Yes (age, gender, tumor location)	No	LOOCV; 1200 epochs for 3-group classification; sequential: IDH 700 epochs; 1p/19q 800 epochs; LR & batch size not specified	Python, SPM12	11
Chang K, et al. (2018) [18]	Training/Internal validation: 446<br>Held-out test: 50	Modified 34-layer ResNet	No	NA	Yes (age)	Rotations, translations, flips, shearing, zooming	LR = 1×10^{-4} (×0.25 after 20 no-improve); BS = 16; momentum = 0.9; epochs ~157; BCE loss	Python, Keras, MATLAB, FSL, Nipype	17
Calabrese E, et al. (2020) [19]	Training/Internal validation (10-fold CV): 199<br>External validation: 57	Cascaded 2D CNN (segmentation); RF classifier	Yes	No fine-tuning	No	No	Patch 96 × 96 × 4; BS = 5; epochs = 20; LR $1 \times 10^{-3}\rightarrow1 \times 10^{-7}$; Adam	Python, scikit-learn, PyRadiomics	19
Ai L, et al. (2022) [20]	Training/Internal validation (10-fold CV): 211<br>Held-out test: 24	3D multi-scale CNN (TDAB/ZAB/FSB blocks)	No	NA	No	Flipping, contrast adjustment, Gaussian sharpen/blurring	LR = 1×10^{-4} (decay 0.9); BS = 8; epochs = 50; cost-sensitive weights	Python, PyTorch, MONAI	15
Chaddad A, et al. (2023) [21]	Training/Internal validation (LOOCV): 58<br>Held-out test: 25	3D CNN: 2 convolutional layers + 2 fully connected layers; 2D CNN: ResNet50, DarkNet53, NasNet-large; PCA applied to CNN features	Yes	Fine-tuned	Yes (age, gender)	No	LR: 0.0005, Momentum: 0.9, Loss: Cross-entropy, Dropout: 0.8	MATLAB, Python, 3D Slicer	17
Chakrabarty S, et al. (2023) [22]	Training/Internal validation (10-fold CV): IDH: 223, 1p/19q: 348<br>Held-out test: IDH: 62, 1p/19q: 117<br>External validation: IDH: 762, 1p/19q: 437	2.5D multi-view CNN (Mask R-CNN-based, late fusion)	No	NA	Yes (age for IDH; location for 1p/19q)	No	NR	NR	20
Chakrabarty S, et al. (2023) [23]	Training/Internal validation (5-fold CV): 223<br>External validation: 323	3D Mask R-CNN (ResNet-101-FPN)	No	NA	No	Mirroring; random rotations	LR = 1×10^{-4}; BS = 4; epochs = 200	Python, PyTorch 0.4.1; scikit-learn; numpy; pandas; seaborn; R (DTComPair, pROC)	19
Chen Q, et al. (2023) [24]	Training: 145<br>Held-out test: 49<br>External validation: 77	WSOFNet: PEM per modality → AMF2M → OPM → FEM (transformer + MBConv) → FC classifier	No	NA	No	Random rotation, translation, shearing, scaling, flipping, Gaussian noise	SGD (momentum 0.9), lr 1e-4, batch 64, epochs 150, ReduceLROnPlateau (patience 8)	NR	15
Chu W, et al. (2023) [25]	Training/Internal validation: 171<br>Held-out test: 19	U-Net++ segmentation backbone with: 1) grading head (GAP + conv), 2) classification head (conv + residual blocks), 3) adversarial discriminator	No	NA	No	No	lr 2e-3; batch 64; 10 000 iterations with poly decay (power 0.99); classification head unfrozen after 1 000 iters	Python, PyTorch	15
Buz-Yalug B, et al. (2024) [26]	Training/Internal validation: 128<br>Held-out test: 34	ResNet50 & VGG16 backbones enhanced with attention gates (after stages 2&3 for ResNet; 3&4 for VGG)	Yes	Fine-tuned	No	Random rotations, width/height shifts, shear, zoom, vertical/horizontal flips	~20–30 epochs (until plateau); hyperparam grid search; batch size NR	Python, Keras, TensorFlow, Slicer3D, FSL, ANTs	14
Calabrese E, et al. (2022) [27]	Training/Internal validation (5-fold CV): 320<br>Held-out test: 80	CNN limb: 3D multiscale deep convolutional autoencoder with residual bottlenecks; Radiomics limb: random forest classifier	No	NA	No	Random dimension swaps, rotations	Adam optimizer; 40 epochs; batch 10; 5-fold CV; Glorot init	Python, TensorFlow 2.4, scikit-learn 0.24, PyRadiomics 2.2.0, FSL, ITK-SNAP	16
Cheng J, et al. (2022) [28]	Training: 148<br>Held-out test: 70	MTTU-Net: CNN-Transformer encoder; U-Net–like decoder (segmentation) + multi-scale classifier (genotyping)	No	NA	No	Random rotation, random cropping, random flipping, intensity shift	Adam optimizer; lr 2e-4 (decaying); batch 2; up to 1 000 epochs	Python, PyTorch	16
Choi Y, et al. (2020) [29]	Training: 45<br>External Validation: 91	V-Net	No	NA	No	No	LR 0.001; 128 epochs; batch 4	Python, PyTorch v1.0; 3D Slicer; R; PyRadiomics	17
Choi YS, et al. (2021) [30]	Training: 727<br>Held-out test: 129<br>External validation: 310	Model 1: modified 3D U-Net; Model 2: 34-layer ResNet-based CNN + FC layers (integrating radiomics + 2D slices + age)	Yes	Fine-tuned	Yes (age)	No	Model 1: 46 epochs; Model 2: 111 epochs warm-up + 72 epochs fine-tuning (LR & batch size NR)	Python, R	20
Gore S, et al. (2021) [31]	Training/Internal validation: 173<br>Held-out test:44	Three-pathway CNN (13 conv layers + 2 FC)	No	NA	No	No	Adam (LR 1e-5); batch 8; ~50 epochs	Python, Keras	14

Karami G, et al. (2023) [32]	Training/Internal validation (5-fold CV): IDH: 92, 1p/19q: 88<br>External validation: IDH: 8, 1p/19q: 12	ResNet10	No	NA	Yes (tumor location)	Flipping, rotation, translation, Gaussian noise	Adam (LR 1e-6; momentum 0.9; weight decay 0.1); LR scheduler (γ=0.1, patience 10); batch 32; epochs 100	Python, PyTorch, FSL (FLIRT), HD-GLIO, 3D Slicer	15
Liu J, et al. (2024) [33]	Training: 78	nnU-Net (for segmentation & habitat construction)	Yes	No fine-tuning	No	No	LR 1e-4; 200 epochs; combined Dice + CE loss; GPU: GTX 2080Ti	Python 3.7, PyTorch 1.8.1, CUDA 11.1, cuDNN 8.0.5, ANTs, 3D Slicer, SPM12, scikit-learn 0.19.1	7
McHugh H, et al. (2023) [34]	Training: IDH: 533, 1p/19q: 149<br>Held-out test: IDH: 205, 1p/19q: 32<br>External validation: IDH: 420, 1p/19q: 114	2D Dense U-Net (dense blocks in U-Net)	Segmentation: Yes; classification: No	Fine-tuned	Yes (age)	Intensity shift, Gaussian noise, random translation, rotation, flipping	LR=1e-4; batch size=8; epochs=200	Python, TensorFlow 2.0, Simple ITK, dcm2niix	17
Moon H, et al. (2024) [35]	Training/Internal validation: 651<br>Held-out test: 119<br>External validation: 108	Score-based diffusion model (augmentation), 3D U-Net, ResNet50 classifier	No	NA	No	Score-based diffusion augmentation; phenotype-based augmentation (size, contrast)	ResNet50: LR=1e-4; Adam; epochs=30 (diffusion params NR)	SPM12, HD-BET, nnUNet, PyTorch 1.1, Python 3.7	20
Nalawade S, et al. (2019) [36]	Training/Internal validation (5-fold CV): 208<br>Held-out test: 52	DenseNet161, ResNet50, Inceptionv4	Yes	Fine-tuned	No	Flips, rotations, translations, shear, zoom, elastic	LR=1e-7; decay=1e-7; momentum=0.8; epochs=200; batch size NR	Python, Keras, PyCharm IDE	15
Nalawade SS, et al. (2022) [37]	Training/Internal validation (5-fold CV): IDH: 142, 1p/19q: 246<br>Held-out test: IDH: 71, 1p/19q: 122	3D Dense-UNet (32×32×32 patch-based)	Yes	Fine-tuned	No	Flipping	LR=1e-5; batch size=4; training time 96–120 h (epochs NR)	Python, Keras, ANTs, FSL BET, NVIDIA Tesla V100 GPUs	15
Pasquini L, et al. (2021) [38]	Training/Internal validation (5-fold CV): 100	4-block 2D CNN (conv-ReLU-BN-pool ×4 → FC → softmax)	No	NA	No	Rotation	LR=1e-4; epochs=500; optimizer=Adam; ~7 s/epoch; 5-fold CV	Python 3, Keras API, NVIDIA CUDA GPU	15
Rui W, et al. (2023) [39]	Training/Internal validation (5-fold CV): 35<br>Held-out test: 7	Inception CNN (dual-path for annotated & unannotated slices)	No	NA	Yes (age, gender)	No	Not explicitly reported	ITK-SNAP	14
Safari M, et al. (2022) [40]	Training/Internal validation (3-fold CV): 89<br>Held-out test: 16	Shuffle-ResNet	No	NA	No	Flipping, rotation; brightness and hue jitter	Up to 150 epochs w/ early stopping (folds stopped at 131/106/96); e.g. LR=3.57×10^{-4}, BS=512, momentum=46.3×10^{-3}	Python 3, PyTorch 1.8, Ray 1.13.0	15
Zhang J, et al. (2024) [41]	Training/Internal validation (5-fold CV): 505<br>External validation: 254	MFEFnet: SFE (ResNet50+SE), AMF (ResNet18), DFF (Transformer-like self-attention + MIL pooling)	No	NA	No	Gaussian noise, flip, intensity shift, rotation	200 epochs; BS=4; LR=5e-3 with one-cycle scheduler	Python, PyTorch, NVIDIA Titan X GPU, nnU-Net	19
Zhang H, et al. (2023) [42]	Training/Internal validation (10-fold CV): 486	CNN+LSTM (eca_nfnet_l0 backbone → sequence modeling)	Yes	Fine-tuned	No	Flip, rotation, translation	BS=32; LR=1e-6; sequence length=11; stride=2; sigmoid; BCE loss	Python, PyTorch, scikit-learn, pycaret, pyradiomics, SimpleITK, FSL	15
Yogananda CGB, et al. (2023) [43]	Training/Internal validation (5-fold CV): 683<br>External validation: 1166	Two 2D nnU-Net networks: T2-net & MC-net	No	NA	No	nnU-NetTrainerV2_DA5 (e.g., rotations, scaling)	5-fold CV; ~5 days per CV	Python, PyTorch, FeTS, MATLAB	18
Zeng H, et al. (2022) [44]	Training/Internal validation (6-fold CV): 110	MDAS segmentation: cyclic GAN + UNet-like module, modified VGG19 for feature extraction	Yes	Fine-tuned	Yes (age)	Translation, normalization, resampling	Segmentation: LR 0.0002 (Adam), BS 4, 8 k iters × 2; Classification: sixfold CV	Python, TensorFlow, pyradiomics	16
Xu Q, et al. (2022) [45]	Training/Internal validation (4-fold CV): 151<br>Held-out test: 37	Multitask ViT (T2net, T1Cnet, TUnet)	No	NA	No	Flipping, rotation	Patch size = 8; 8 transformer layers; LR ≈ 1e-4; BS 32; epochs 150 × 4 runs	Keras, Python, PyCharm	11
Wu J, et al. (2022) [46]	Training: 207<br>Held-out test: 52<br>External validation: 234	Swin Transformer, ResNet-101	ResNet-101: Yes; Swin: No	Fine-tuned	Yes (age, tumor location)	Geometric and intensity transforms	Swin: LR 1×10^{-5}, BS 32, 300 iters; ResNet: LR 1×10^{-4}, BS 32, 300 iters; Adam (β_1 = 0.9, β_2 = 0.99); early stopping; StepLR scheduler	Python, PyTorch	17
Wei Y, et al. (2021) [47]	Training/Internal validation (5-fold CV): 298<br>Held-out test: 74	Graph Neural Network (3 graph-conv + embedding + 2 FC); benchmarks: 3D-CNN, 3D-DenseNet	No	NA	No	Random edge drop	Adam (binary cross-entropy, L2 = 0.001); LR decay; early stopping; dropout; exact BS/epochs NR	Python, FSL (FLIRT, BET), ANTs	15
Wang Y, et al. (2021) [48]	Training/Internal validation (5-fold CV): 121	3D multi-task residual U-Net with skip connections & residual units	No	NA	No	Random shifting, flipping	LR: 0.0001 (backbone & seg branch), 0.00005 (IDH branch); BS 2; cosine annealing; epochs NR	Python	15
Wei Y, et al. (2022) [49]	Training/Internal validation (5-fold CV): 298<br>Held-out test: 74	4×3DConv + 3 FC, 4×GraphConv + 3 FC	No	NA	No	Rotation	Adam (LR 0.001→0.0001), 200 epochs, early stopping, weight decay, dropout; batch size NR	Python, PyTorch Geometric	14

Wei Y, et al. (2023) [50]	Training: 270<br>Held-out test: 117	Image Encoder: 5×Conv + 3 FC; Geometric Encoder: NNConv, Brain Network Encoder: GATConv	Yes	Fine-tuned	No	Image and point cloud Rotation	Contrastive: 1000 epochs, LR 0.001 (decay 90%/50 epochs), BS 50; Population graph: 200 epochs, BS 20	F Python, SL, ANTs, PyTorch & PyTorch Geometric	11
Tripathi PC, et al. (2023) [51]	Training/Internal validation (10-fold CV): IDH: 377, 1p/19q: 275	Segmentation: 3D CNN, Classification: multi-task CNN (residual blocks + FC)	No	NA	No	Random flip, intensity shift, rotation, random cropping	Seg: BS 4, 200 epochs, LR 0.001 (×0.5/50 epochs); Cls: BS 4, 160 epochs, LR 0.0001 (×0.5/quarter); Adam	Python, Tesla K80	16
Shi X, et al. (2023) [52]	Training: 366<br>Held-out test: 122	SA-Net (self-attention) + Pyradiomics, BRNN classifiers + linear regression fusion	No	NA	No	No	BS 16; LR 0.0001; epochs NR	Python, Pyradiomics 2.0.0, ITK/SimpleITK	9
Shi X, et al. (2023) [53]	Training: 148<br>Held-out test: 70	TransBTS segmentation & IDH head, MLP fusion	Yes	Fine-tuned	No	No	Loss: Dice for seg; CE for IDH with uncertainty weighting; params NR	NR	14
Yan J, et al. (2022) [54]	Training: 330<br>Held-out test: 123<br>External validation: 102	ResNet-34-based 3D CNN	No	NA	No	Random rotation, shear, zoom	Adam (LR 0.001; batch 32; epochs NR)	ITK-SNAP	16
Kihira S, et al. (2022) [55]	Training: 176<br>Held-out test: 32<br>External validation: 31	U-Net seg. + DenseNet121/ResNet50 classifier	Yes	Fine-tuned	Yes (age, gender)	No	Batch 16; LR 0.001; BCE loss; 7-fold CV	Olea Sphere, Python 3.8.10, Pyradiomics	15
Sohn B, et al. (2021) [56]	Training: 292<br>Held-out test: 126	BR & ECC pipelines (SVM with linear kernel)	No	NA	No	MLSMOTE oversampling	Hyperparam tuning for LASSO & SVM (C) (values NR)	Python (scikit-learn 0.21.2), R 3.5.1, Pyradiomics	12
Buda M, et al. (2019) [57]	Training/Internal validation (22-fold CV): 110	U-Net for segmentation	No	NA	Yes (in separate clinical model; not integrated into DL)	Rotation, scaling, slice oversampling	22-fold CV; augmentation as above	Python	17
Ali MB, et al. (2023) [58]	Training/Internal validation (5-fold CV): 167	EtFedDyn: 2-stream CNN with attention fusion, bilinear & FC layers	No	NA	No	Flipping, rotations	FL sim: rounds 50; local epochs 5; batch 50; η = 0.01; decay = 0.001; α = 0.01; central: batch 50; LR = 0.001; decay = 0.0001; iter 50	Python, PyTorch, FSL, ANTs	14
Chen M, et al. (2024) [59]	Training/Internal validation: 797<br>Held-out test: 199<br>External validation: 157	ViT autoencoder (16 MHA blocks) + ViTClassifier_block4/16	Yes	Fine-tuned	No	Random coarse dropout masking during pretraining	Pretraining: 500 epochs, Adam lr 1e-5; Classification: 300 epochs, batch 24, lr 1e-5 with cosine decay; 8× NVIDIA A100 GPUs	Python, PyTorch, MONAI, HD-BET	18
Elyassirad D, et al. (2024) [60]	Training/Internal validation: 396<br>Held-out test: 99	ResNet	No	NA	No	Flipping, rotations, zooming, intensity shift, scaling, Gaussian noise, contrast adustment, Gaussian smoothing, elastic deformation, grid distortion, histogram shift	Weighted binary cross-entropy; dropout; early stopping; LR reduction; checkpoint callbacks; specific hyperparameters NR	Python, MONAI	15
Fayyaz M, et al. (2023) [61]	Training: 111<br>Held-out test: 22	Xception, ResNet152V2, InceptionV3, InceptionResNetV2, NASNetLarge	Yes	Fine-tuned	No	Rotations, translations, scaling, flipping	Classifier FC layers: (512,256,128), (256,256,128), (256,128)	Python, Keras	13
Ge C, et al. (2018) [62]	Training/Internal validation: 95<br>Held-out test: 32	Multistream 2D CNN (7-layer per sensor) with bilinear fusion & FC layers	No	NA	No	Multi-view slice extraction, flipping, random rotations	Adagrad, lr 0.0001; Dropout 0.5 in FC; L2 reg 0.0001; Epochs: ~50 (Case-A, params at epoch 13), 300 (Case-B, epoch 205)	Python, Keras	10
Gómez Vecchio T, et al. (2024) [63]	Training/Internal validation: 255<br>Held-out test: 59<br>External validation: 155	Convolutional autoencoders; ResNet152, DenseNet, MobileNet	No	NA	Yes (age, tumor location in separate clinical model; not integrated into DL)	No	Hyperparameters via grid search over 208 models (see Supplementary)	Python, IBM SPSS Stats 28	19
Hosseini SA, et al. (2023) [64]	Training/Internal validation (CV): 57	Various classifiers (GNB, DT, RF, LR, SVC, MLP, etc.)	No	NA	No	CTGAN synthesis, random noise	NR	Python, PyRadiomics, 3D Slicer	14
Jeon YH, et al. (2025) [65]	Training: 218	Regression	Yes	No fine-tuning	Yes	No	NR	Python, HD-GLIO, FSL FAST, MedCalc	7
Jian J, et al. (2025) [66]	Training: 294<br>Held-out test: 124	Autoencoder (7 layers)	No	NA	No	No	NR	Python, PyRadiomics, ANTs, skull-strip tool, R	14
Li D, et al. (2025) [67]	Training: 239<br>Held-out test: 103<br>External validation: 60	ResNet101 + SVM + logistic-regression nomogram (DLRN)	Yes	Fine-tuned	Yes	No	LR 0.001; BS 100; Epochs 100	Python, ANTs, SimpleITK, ITKsnap, PyRadiomics, Python 3.7, R 4.2	17
Li X, et al. (2021) [68]	Training/Internal validation (5-fold CV): 263	ResNet50 & ResNeSt50 with early/mid/late fusion	Yes	Fine-tuned	No	Translation, rotation, flipping	5-fold CV; NR hyperparams	labelImg, Python	11

Li Y, et al. (2021) [69]	Training/Internal validation (5-fold CV): IDH: 780, 1p/19q: 254<br>Held-out test: IDH: 226, 1p/19q: 80	Radiomics: SVM (LASSO), DCNN: ResNet18 ensemble	No	NA	Yes (age, gender)	No	5-fold CV (radiomics); ensemble of 5 DCNN; params NR	Python, PyRadiomics, scikit-learn, PyTorch, R, t-SNE, Circos	14
Lost J, et al. (2024) [70]	Training/Internal validation (5-fold CV): 377<br>External validation: 207	XGBoost classifier	No	NA	No	SMOTE oversampling	5-fold CV × 10 iterations; RandomizedSearchCV; seed 123	Python, Pyradiomics v3.0.1, scikit-learn, XGBoost	16
Nishikawa T, et al. (2023) [71]	Training: IDH: 232, 1p/19q: 106<br>Held-out test: IDH: 26, 1p/19q: 12<br>External validation: IDH: 202, 1p/19q: 146	1-conv (5×5) + max-pool + FC SoftMax	No	NA	No	Rotation, flipping	LR = 0.01; 500 iterations; init weight = 1; min-max norm; batch size NR	Python, Azure ML Studio, EZR	17
Park JE, et al. (2021) [72]	Training: 118<br>Held-out test: 44	StyleGAN2 (synthesis), logistic regression	No	NA	Yes (age, gender)	GAN-based image generation, geometric transforms	StyleGAN2: lr 2×10^{-3}; Adam; batch 32; trained on 80 k→4 M images	TensorFlow (StyleGAN2), SPM12, HD-BET	13
Sacli-Bilmez B, et al. (2025) [73]	Training/Internal validation: 180<br>Held-out test: 45	1D-CNNs: BM, DSN, ADSN	No	NA	Yes (age)	No	Hyperparameters tuned	Python, MATLAB 2023a	14
Sairam VA, et al. (2023) [74]	Training: 57<br>Held-out test: 14	VGG16, ResNet101, DenseNet121, EfficientNetV2S, InceptionV3 (multi-task CNN)	No	NA	No	No	15 epochs; batch size 64; Adam optimizer; binary cross-entropy loss	Python, PyQt	13
Santinha J, et al. (2024) [75]	Training/Internal validation (5-fold CV): 142<br>External validation: 76	Logistic regression classifier, segmentation via HD-BET/HD-GLIO	Yes	No fine-tuning	No	No	Ridge regularization optimized by AUC; Platt calibration; balanced class weights	Python, scikit-learn v1.1.3, Fairlearn v0.7.0, PyRadiomics v3.0.0, HD-BET, HD-GLIO	19
Shi X, et al. (2022) [76]	Training/Internal validation (10-fold CV): 366<br>Held-out test: 122	SA-Net, Bayesian Regularization Neural Network, linear regression for fusion	No	NA	No	No	Adam optimizer; batch size 16; LR 0.001 (proposed); batch size 8; LR 0.001; epochs NR	PyRadiomics 2.0.0, SimpleITK, Python, NVIDIA RTX 3090 GPU	10
Stadlbauer A, et al. (2024) [77]	Training/Internal validation (10-fold CV): 166<br>Held-out test: 16<br>External validation: 33	ABoost, MLP, RF, 1D-CNN, LSTM	No	NA	No	SMOTE oversampling	1D-CNN: up to 300 epochs; dropout 0.2; Adam optimizer; LSTM: 300 epochs; LR 0.001; batch size 28/10	Weka v3.8.5, KNIME v4.7.4, Python, PyRadiomics, MATLAB	14
Taha B, et al. (2021) [78]	Training/Internal validation: 188<br>External validation: 138	CNNs, GAN (anomaly detection), SVM, GMM, RF, MLP	No	NA	Yes (age)	No	100 iterations for train-test splits; GMM K = 1–4; other parameters NR	Python, PyRadiomics, 3D Slicer	17
Usuzaki T, et al. (2024) [79]	Training: 271<br>Held-out test: 35<br>External validation: 291	Variable Vision Transformer (vViT) + MLP head	No	NA	Yes (age, gender)	Flip, perspective transform, invert, posterize, solarize, equalize	BCE loss; Adam (β_1=0.9, β_2=0.999, ε=1e-8); patch 32; 2 heads; MLP dim 64; depth 2; pretrain 1 layer; FT linear; batch size & epochs NR	PyTorch v1.7.1, Python 3.8.2, PyRadiomics, Pillow	17
Wang Y, et al. (2025) [80]	Training (CV, bootstrapping): 279<br>Held-out test: 120<br>External validation: 228	LASSO models (single-task, multi-task, collaborative)	Yes	No fine-tuning	Yes (age, gender)	No	LASSO λ=0.001; λ iterated 0.1–0.0001 (1-SE rule); bootstrap 100 reps (80% train; 20% CV)	Python, PyRadiomics v3.0, ITK-SNAP, Elastix, HD-BET, SciPy	20
Wankhede DS, et al. (2022) [81]	Training/Internal validation: 202<br>Held-out test: 25	Ensemble CNN (32-filter conv, MaxPooling2D), compare VGG16/19	No	NA	No	No	LR=1e-4; epochs=80; batch size=16; categorical cross-entropy	Python, Keras	11
Yang Z, et al. (2025) [82]	Training: IDH: 597, 1p/19q: 226<br>Held-out test: IDH: 150, 1p/19q: 56<br>External validation: IDH: 64, 1p/19q: 39	Swin Transformer	Yes	Fine-tuned	No	Rotation, translation, flip, scaling	AdamW (weight decay 0.05); LR=1e-4; 100 epochs; linear warm-up 500 steps (warm-up ratio 0.001)	MMDetection v2.3.0, PyTorch v2.1.0, Python 3.10	17
Yu D, et al. (2024) [83]	Training (bootstrapping): 414<br>Held-out test: 72<br>External validation: 178	ViT + linear classifier	Yes	Fine-tuned	No	No	63 epochs; LR & batch size NR	Python 3.9, R 4.1.3, Tofu-fish	22
Yuan J, et al. (2024) [84]	Training/Internal validation (5-fold CV): 206<br>Held-out test: 20	VGG16 (fixed) + SVM (RBF)	Yes	No fine-tuning	Yes (age)	No	Nested 5-fold CV; inner 20% grid search for SVM C, γ; CNN fixed	ITK-SNAP, FSL, MATLAB R2021a/R2020b, Python	15
Yuan Y, et al. (2023) [85]	Training/Internal validation (5-fold CV): 84	2D CNN (3× small conv, 4×4 conv, GAP, 3× FC)	No	NA	No	No	35 epochs; LR 0.01→0.001→0.0001; batch 32; Adam (weight decay 0.001)	MATLAB R2020a/R2022b, scikit-learn, Python, NVIDIA GTX 3080	12
Zhang S, et al. (2021) [86]	Training/Internal validation (4-fold CV): 162	Gradient Boosting (TPOT AutoML)	Yes	No fine-tuning	No	No	TPOT: 50 generations, pop 100; inner 10-fold CV; outer 4-fold CV	NiftyNet, ITK-SNAP, Python, Pyradiomics v2.0.0, FSL, TPOT	14
Zhang X, et al. (2021) [87]	Training: 453<br>Held-out test: 49	Modality Self-Attention Net, baselines	No	NA	No	Flip, uniform resolution	100 epochs; batch 8; LR 0.01; Adam	Python	9
Zhao K, et al. (2023) [88]	Training/Internal validation: 150	Custom U-net, ResNet152	No	NA	No	Flip, noise injection	LR 1e-4; momentum 0.9; weight decay 1e-4; LR decay 5%/5 rounds; 6 runs	3D Slicer, UMAP, Python, Quadro RTX 6000	15

Zhu Z, et al. (2024) [89]	Training: 426<br>Held-out test: 67<br>External validation: 46	RF for radiomics, ensemble DL for segmentation	Yes	No fine-tuning	No	No	NR	Python 3.7.13, SimpleITK, Pyradiomics	16
Aliotta E, et al. (2019) [90]	Training: IDH: 25, 1p/19q: 16<br>Held-out test: IDH: 16, 1p/19q: 10	DiffNet (MLP, 2 hidden layers) + logistic regression	Yes	No fine-tuning	No	No	Bootstrapped LR with imbalance-adjusted resampling; split 60/40	MATLAB, DeepMedic (BCIPT), Python, MATLAB radiomic toolbox	13
Alom Z, et al. (2023) [91]	Training/Internal validation (5-fold CV): IDH: 78, 1p/19q: 51	Radiomic: DNN (7 FC layers), RF, SVM; DCNN: VGG-Net, ResNet50, DenseNet	No	NA	No	No	DCNN: Adam; batch=4; epochs=150; early stopping (p=15);<br>10 bootstraps×5-fold CV; Radiomic: 5-fold CV	Python, scikit-learn, TensorFlow, R, Bioconductor	14
González SR, et al. (2019) [92]	Training/Internal validation: IDH: 84, 1p/19q: 85<br>Held-out test: IDH: 15, 1p/19q: 15	Inception v3, ResNet50	Inception v3: No;<br>ResNet50: Yes	Fine-tuned	No	No	50 epochs; Adam (LR 1e-4); batch=8; LR ↓×0.1 after 10 stable epochs; early stopping after 10 stable epochs	Python, Keras, FSL, ANTs	14
Riahi Samani Z, et al. (2023) [93]	Training/Internal validation: 275	CNN (6 conv layers + FC + softmax)	No	NA	No	Random patch shifting	Weight decay 5×10^{-5}; momentum 0.9; LR 1×10^{-4}; batch & epochs NR	FSL, DIPY, ANTs, DeepMedic, Python	13
Sun X, et al. (2024) [94]	Training/Internal validation (5-fold CV): IDH: 358, 1p/19q: 83<br>Held-out test: IDH: 66, 1p/19q: 20	3D U-Net (segmentation),<br>SVM / LR / DT / RF / XGB (classifiers)	Yes	Fine-tuned	No	Random cropping	SGD + Nesterov (momentum 0.99); lr=0.001×(1–epoch/200)^0.9; pretrain 200 epochs; up to 500 epochs; early stopping (patience 20)	Python, PyTorch 1.12, PyRadiomics, 3D Slicer, CaPTk, FeTS, SPSS	16
Zhao Y, et al. (2023) [95]	Training: 236<br>Held-out test: 80<br>External validation: 208	Improved U-Net + CRNN (UCNet / F-UCNet)	No	NA	No	Rotation, reflection, intensity normalization, resampling	Feature extractor: lr=1e-5, bz=4, 300 epochs; CRNN: lr=1e-4, bz=40 / 30, dropout 0.5	Keras, Python, MATLAB 2016, SPSS	16
Zhang L, et al. (2024) [96]	Training: 213<br>Internal Validation: 53<br>External Validation: 200	ViT (MRI), DenseNet-121 + FCN (WSI), stacking meta-learner	Yes	Fine-tuned	No	Flip, translate, rotate, Gaussian noise, projective transform	Lr= 1e-4, batch =8, max iterations=500; StepLR(step=50, γ=0.1); focal loss	Python, PyTorch, Adam optimizer, StepLR scheduler	15
Yogananda CG, et al. (2020) [97]	Training/Internal validation (3-fold CV): 368	3D Dense-UNet (segmentation + voxel classification, dual volume fusion & voting)	Yes	Fine-tuned	No	Flip, translate, rotate, Gaussian and salt-pepper noise, projective transform, down sampling	Lr= 1e-5, batch =15, max iterations=100	Python, Keras, MATLAB, R	15
Akkus Z, et al. (2016) [98]	Training/Internal validation: 109<br>Held-out test: 30	Multi-branch, multi-scale CNN	No	NA	No	Translations, rotations, flips	Lr: 1e-3, batch 32, stop when Δval loss < 0.02 for 10 epochs	Python, Keras, FSL (BET), ANTs	12
Cao S, et al. (2024) [99]	Training/Internal validation (10-fold CV): IDH: 720, 1p/19q: 490<br>Held-out test: IDH: 85, 1p/19q: 85<br>External validation: IDH: 149, 1p/19q: 171	Two-stage: 3D U-Net → ResNet + GCN	Yes	Fine-tuned	No	Flipping, translation, normalization	Lr: 1×10^{-5}, batch 24, AdamW, weight decay 1×10^{-2}, 200 epochs	Python, PyTorch (CUDA 11.2), NVIDIA RTX 3090 GPU	18
Hu Z, et al. (2023) [100]	Training: 150<br>Held-out test: 106	Three-branch DenseNet (one per modality) + bilinear pooling + Softmax	No	NA	No	No	Lr: 1e-3, batch: 64, max 500 iters, weight decay 1×10^{-4}, dropout, exp LR decay, thresh 0.5	ITK-SNAP, SPSS v26.0,<br>R v3.6.1, Python	12
Farahani S, et al. (2025) [101]	Training/Internal validation (5-fold CV): IDH: 1705, 1p/19q: 544<br>External Validation: IDH: 1492, 1p/19q: 665	MTS-UNET multi-task: foundation model built upon BrainSegFounder	Yes	Fine-tuned	No	Flipping, rotation, intensity scaling, elastic deformation; dropout	lr 1e-4, batch 2; up to 100 epochs, early stopping (patience 5)	I3CR-WANO, R v4.4.1, Python, PyTorch, MONAI, scikit-learn	19
Niu W, et al. (2025) [102]	Training/Internal validation: 1185<br>External Validation: 623	Ensemble stacking of 2D CrossFormer, 2.5D CrossFormer, radiomics	No	NA	No	Flipping; cropping	lr: 1e-3, batch: 64, Adam; dropout, (epochs not specified)	ITK, Python, PyRadiomics, scikit-learn, R, Onekey AI platform	21
Wu X, et al. (2024) [103]	Training: 1352, Held-out test: 579<br>External Validation: 845	Segmentation: mmFormer (Transformer-based), classification: 3D ResNet-10	Segmentation: Yes; classification : No	No fine-tuning	No	Rotation, scale transforms, elastic deformation	lr: 0.001, batch: 32, loss: cross-entropy (classification)	Python 3.6, PyTorch 0.4.0, NVIDIA RTX 3080 Ti, R 4.2.3 with DESeq2, clusterProfiler, GSVA, limma, maftools, ESTIMATE, maxstat	21
Chen Q, et al. (2025) [104]	Training/Internal validation (5-fold CV): 1126<br>External validation: 680	Deep CNN–based multi-task feature extraction (CMTLNet): SFTFP module (mask-guided tumor-aware feature learning), UFE module (orthogonal projection of task-unique features), UCFC module (attention-based fusion)	No	NA	No	Random cropping	Initial lr = 1×10^{-4}, Optimizer: Adam, momentum = 0.9, ReduceLROnPlateau (patience = 8), batch: 6, epochs: 60 (CFE only) + 40 (full network)	FSL BET (skull stripping), ANTsPy (rigid registration), PyRadiomics (1578 radiomic features), Python, PyTorch, SHAP (deep-feature interpretability)	20

7. Publication Bias

7.1. IDH mutation Prediction in Internal Validation Datasets

7.1.1. Pooled sensitivity estimate

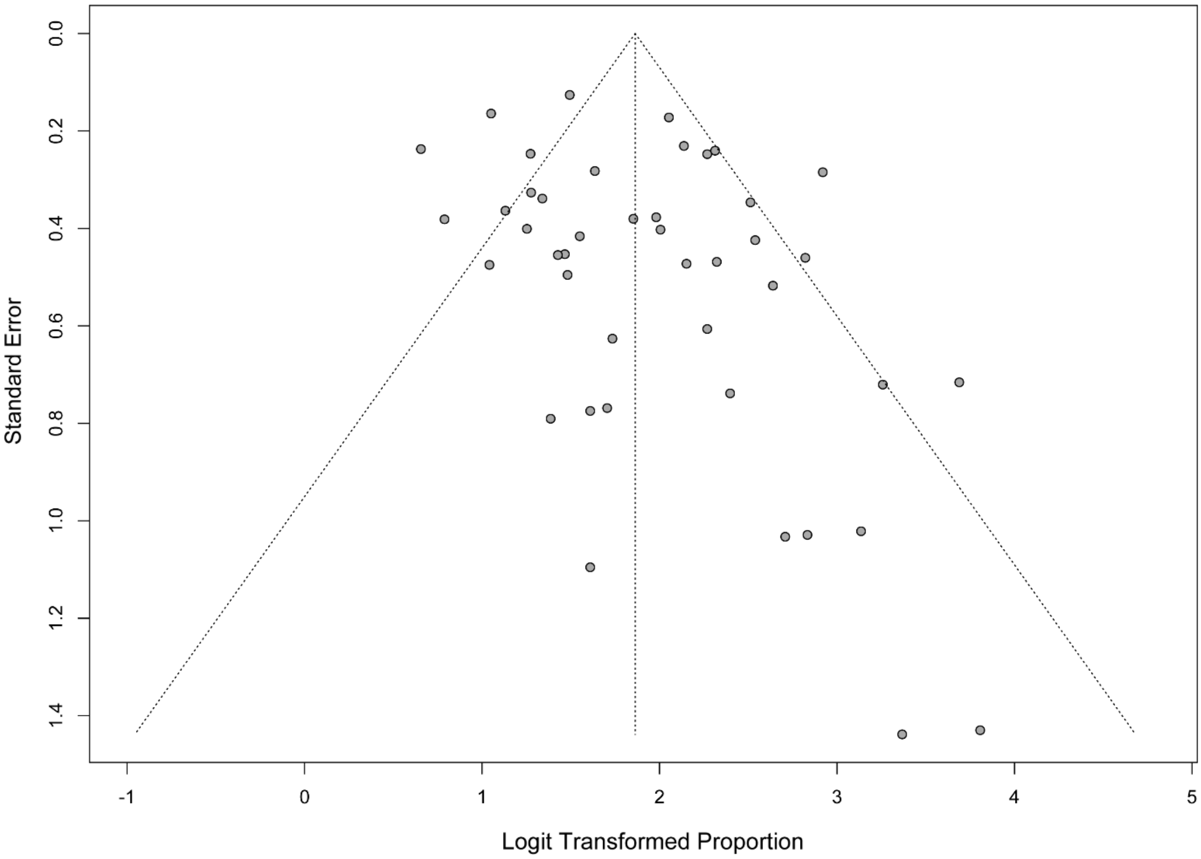

Figure 1. Funnel plot of the distribution of studies on IDH status prediction in internal validation sets, with each point corresponding to a study.

Linear regression test of funnel plot asymmetry

Test result: t = 2.30, df = 41, p-value = 0.0268
Bias estimate: 1.1983 (SE = 0.5218)

Details:
- multiplicative residual heterogeneity variance (tau^2 = 2.9853)
- predictor: standard error
- weight: inverse variance

Applying Trim and Fill to assess publication bias

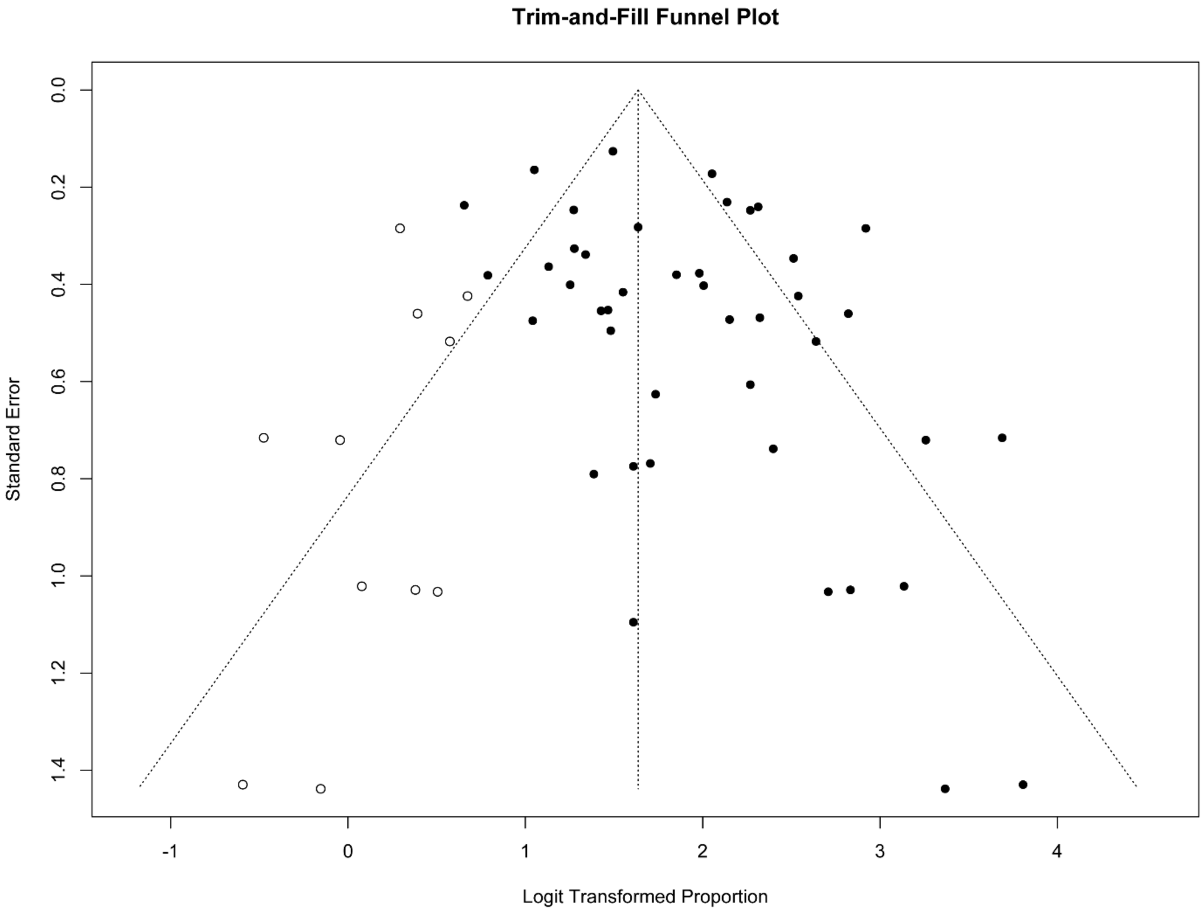

Figure 2. Funnel plot of studies reporting sensitivity for IDH mutation status prediction in the internal validation cohort after applying the Trim-and-Fill method. Adjustment for 11 imputed studies yielded a pooled sensitivity of 0.84 [95% CI: 0.80–0.87].

7.1.2. Pooled specificity estimate

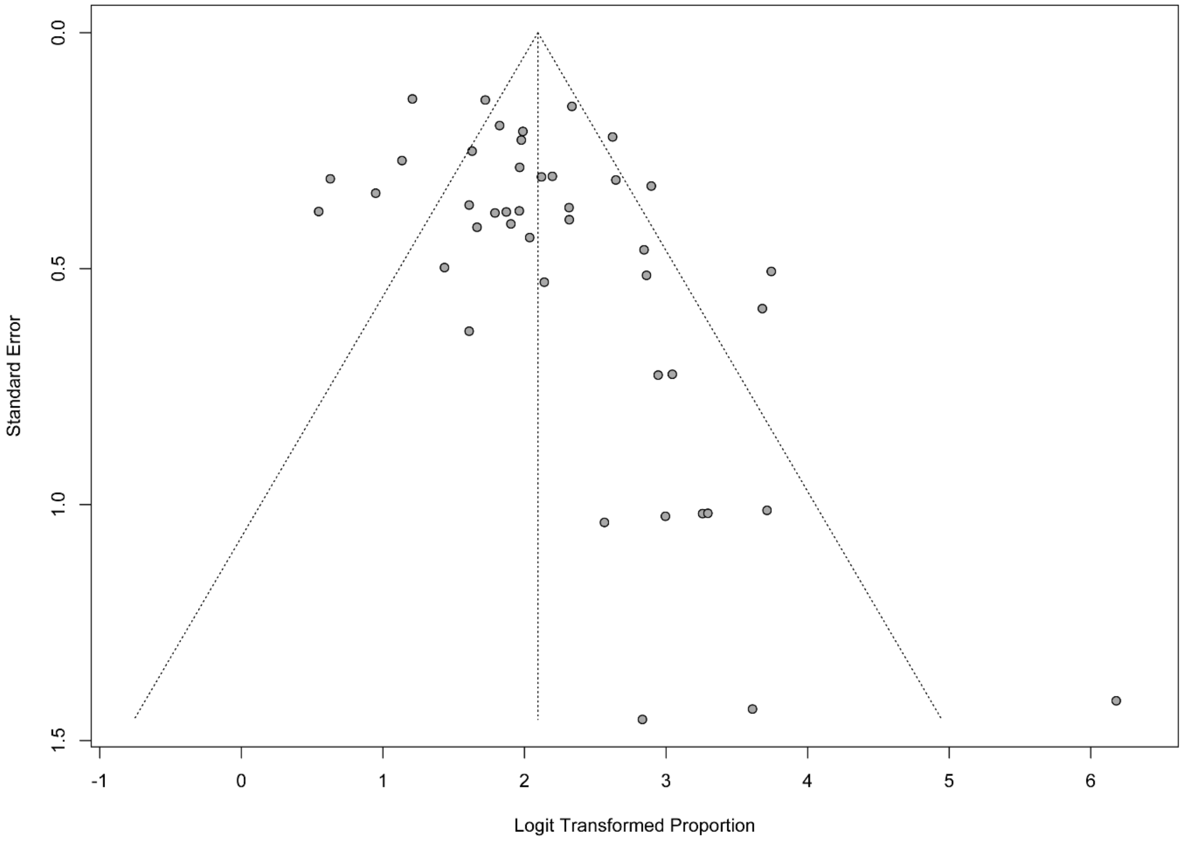

Figure 3. Funnel plot of the distribution of studies on IDH status prediction in validation cohorts, with each point corresponding to a study.

Linear regression test of funnel plot asymmetry

Test result: t = 2.84, df = 41, p-value = 0.0071
Bias estimate: 1.5973 (SE = 0.56)

Details:
- multiplicative residual heterogeneity variance (tau^2 = 3.4062)
- predictor: standard error
- weight: inverse variance

Applying Trim and Fill to assess publication bias

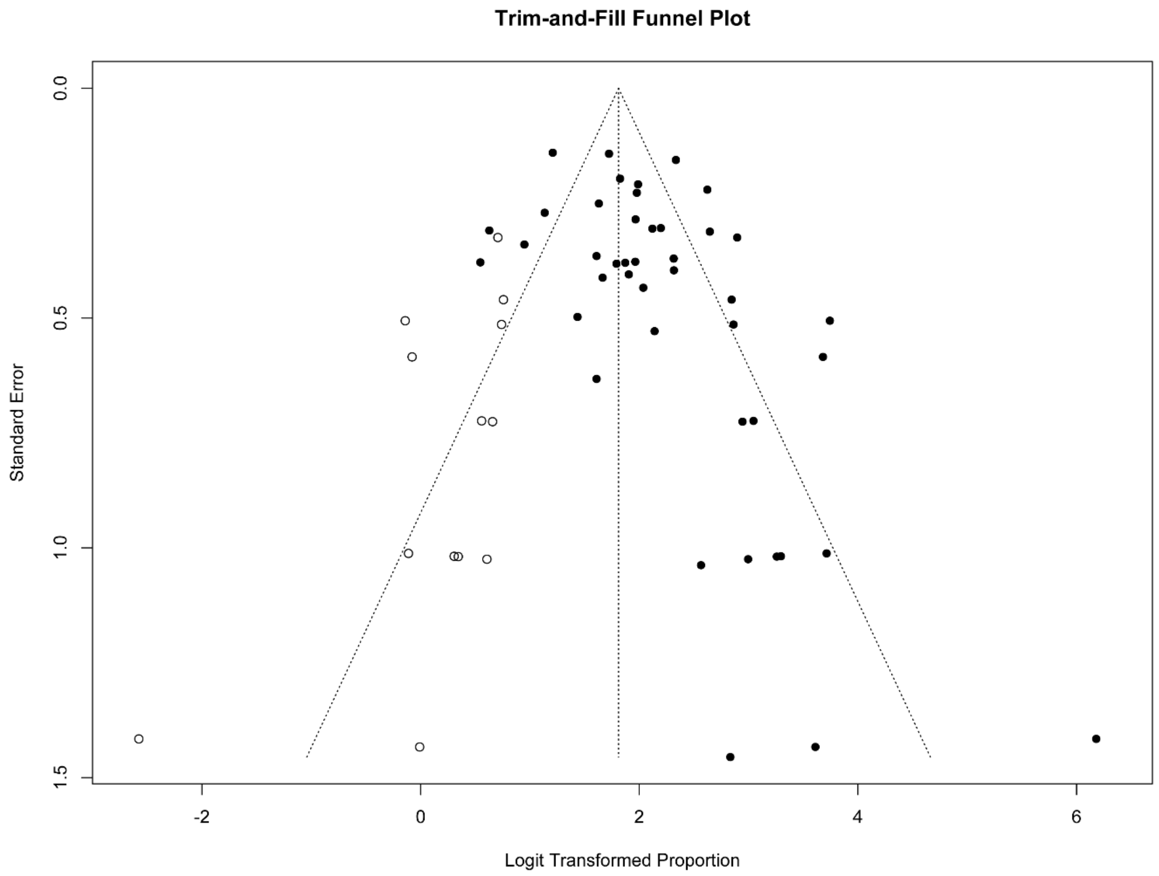

Figure 4. Funnel plot of studies reporting specificity for IDH mutation status prediction in the internal validation cohort after applying the Trim-and-Fill method. Adjustment for 13 imputed studies yielded a pooled specificity of 0.86 [95% CI: 0.83–0.89].

7.2. IDH mutation Prediction in Test Cohorts

7.2.1. Pooled sensitivity estimate

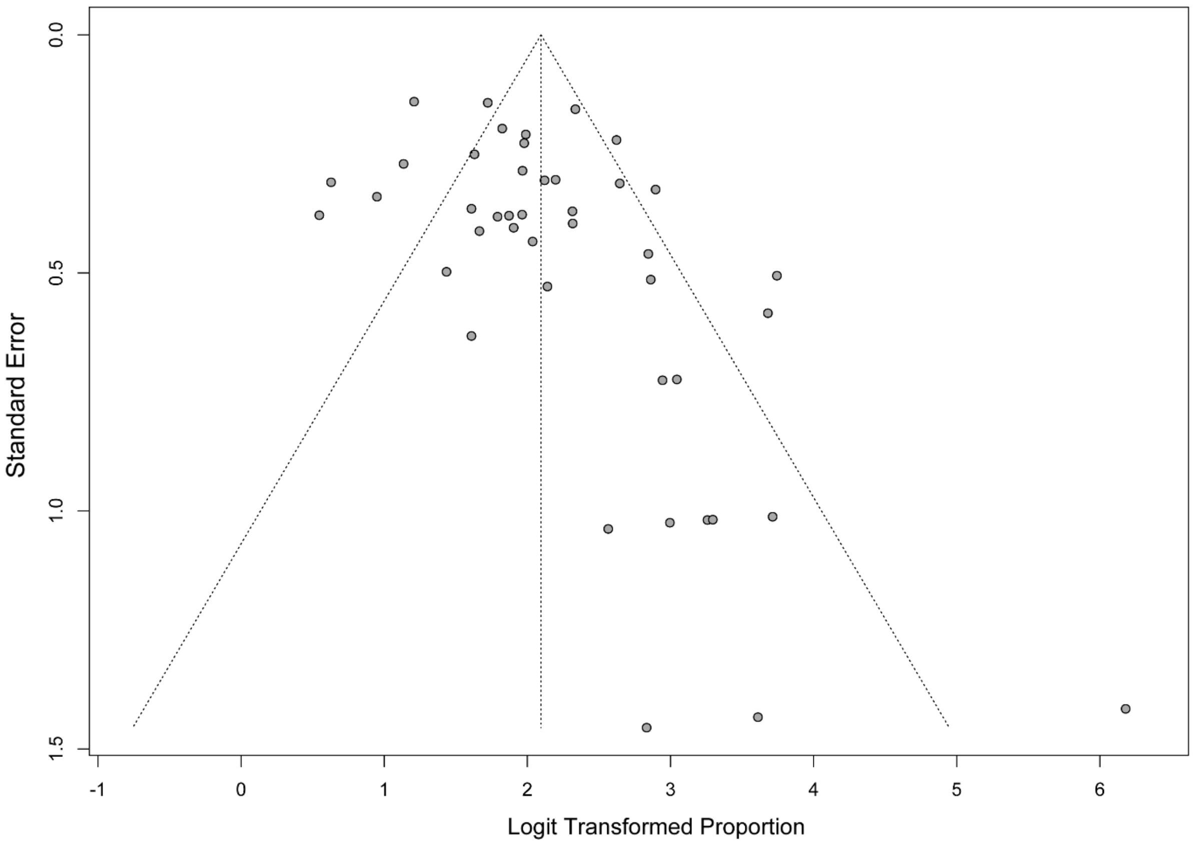

Figure 5. Funnel plot of the distribution of studies on IDH status prediction in test cohorts, with each point corresponding to a study.

Linear regression test (Egger's test) of funnel plot asymmetry

Test result: t = 3.29, df = 50, p-value = 0.0018
Bias estimate: 1.3425 (SE = 0.4080)

Details:
- multiplicative residual heterogeneity variance (tau^2 = 2.2208)
- predictor: standard error
- weight: inverse variance

Applying Trim and Fill to assess publication bias

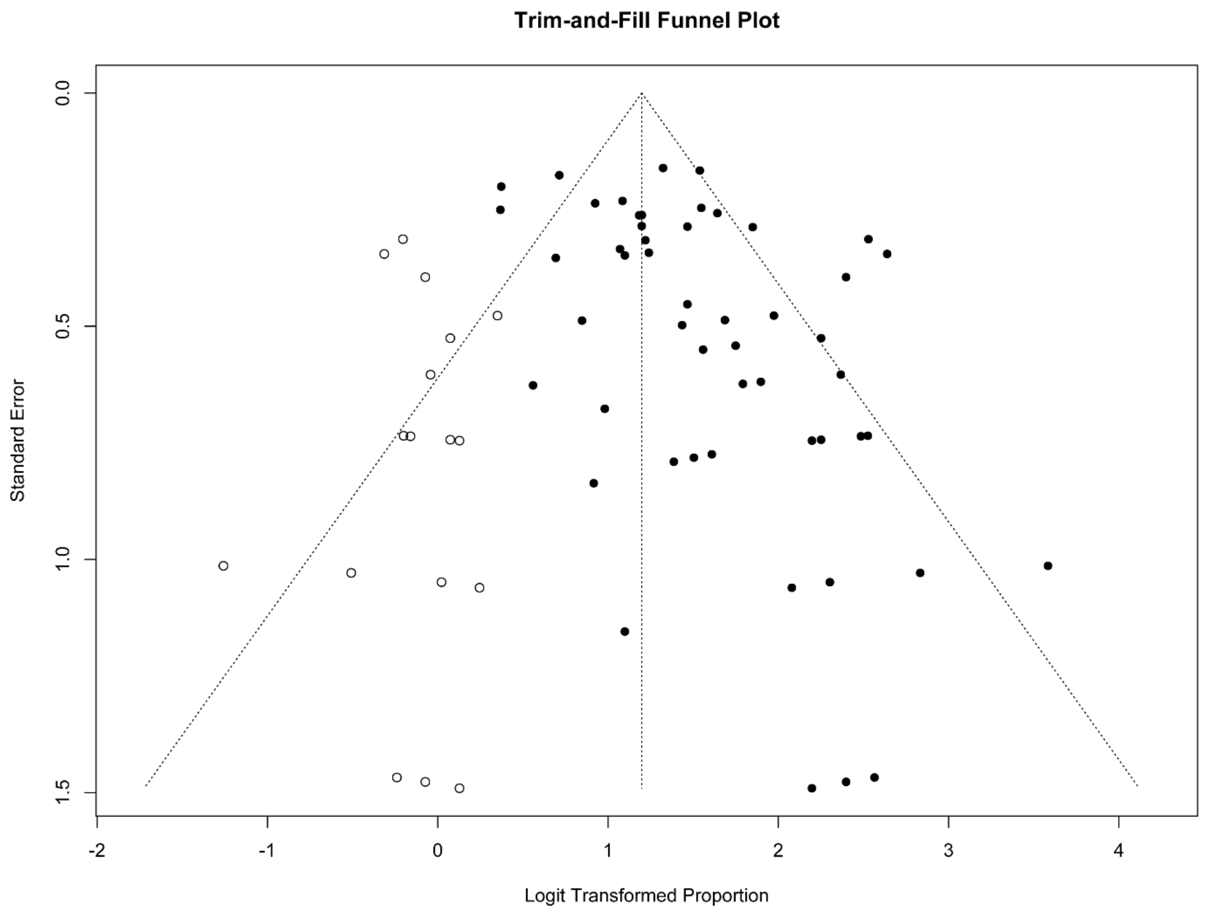

Figure 6. Funnel plot of studies reporting sensitivity for IDH mutation status prediction in the test cohorts after applying the Trim-and-Fill method. Adjustment for 18 imputed studies yielded a pooled specificity of 0.77 [95% CI: 0.73; 0.80].

7.2.2. Pooled specificity estimate

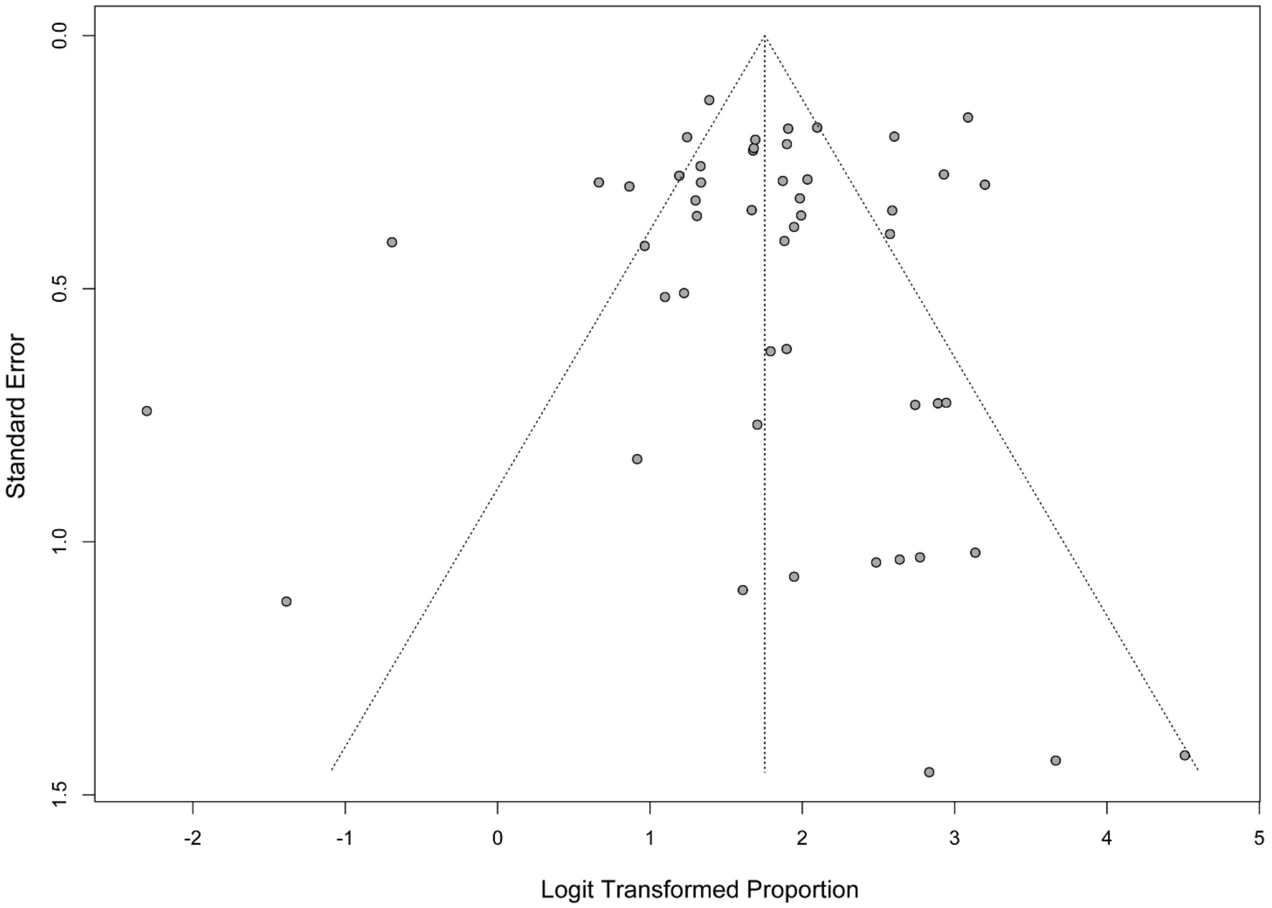

Figure 7. Funnel plot of the distribution of studies on IDH status prediction in test cohorts, with each point corresponding to a study.

Linear regression test of funnel plot asymmetry

Test result: t = -0.51, df = 50, p-value = 0.6136
Bias estimate: -0.3378 (SE = 0.6647)

Details:
- multiplicative residual heterogeneity variance (tau^2 = 5.8451)
- predictor: standard error
- weight: inverse variance

7.3. 1p/19q Codeletion Prediction in Internal Validation Sets

7.3.1. Pooled sensitivity estimate

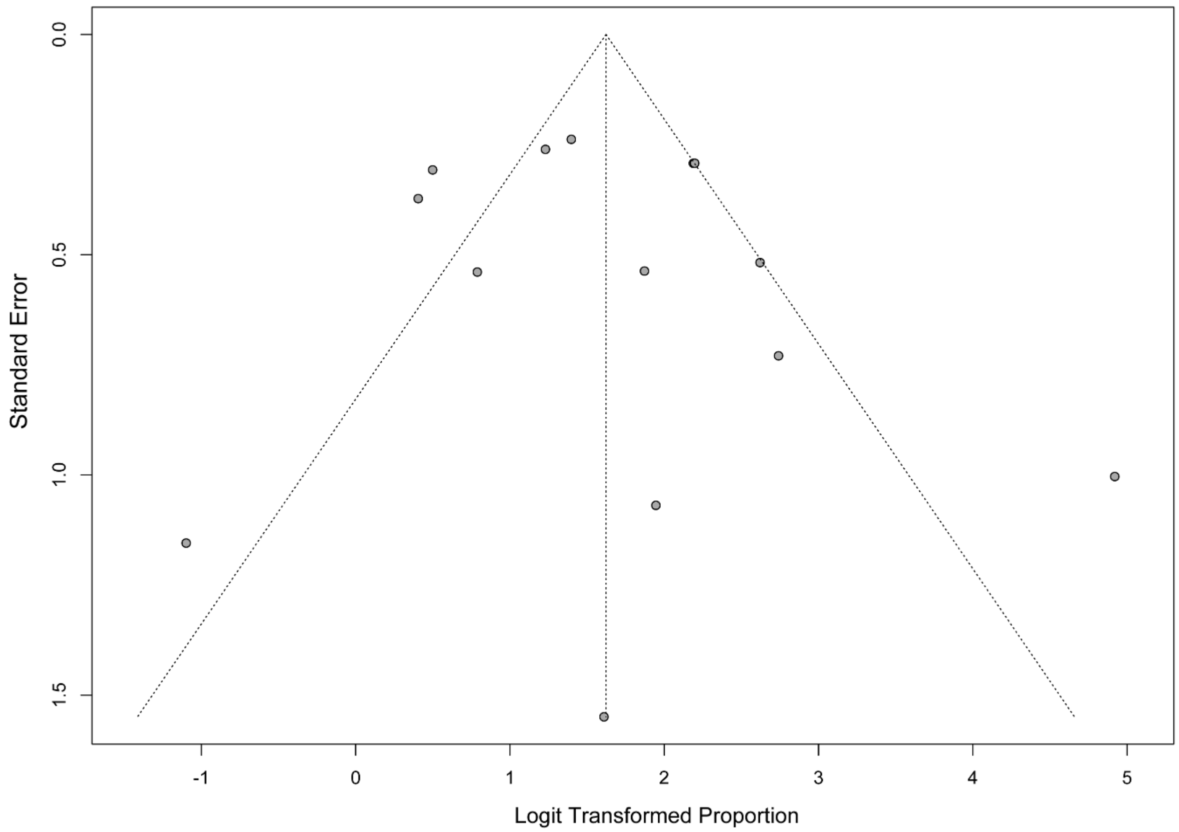

Figure 8. Funnel plot of the distribution of studies on 1p/19q codeletion status prediction in internal validation cohorts, with each point corresponding to a study.

Linear regression test of funnel plot asymmetry

Test result: t = 0.59, df = 12, p-value = 0.5631
Bias estimate: 0.7438 (SE = 1.2507)

Details:
- multiplicative residual heterogeneity variance (tau^2 = 4.7241)
- predictor: standard error
- weight: inverse variance

7.3.2. Pooled specificity estimate

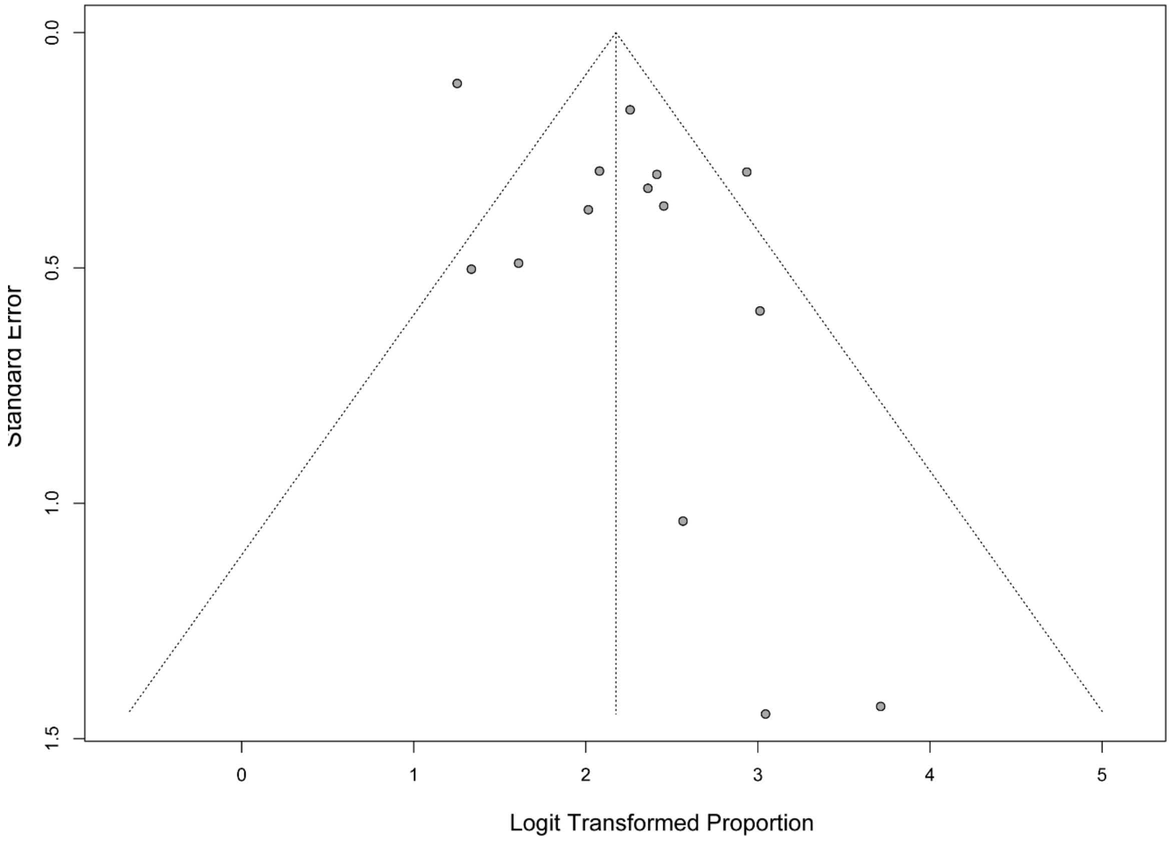

Figure 9. Funnel plot of the distribution of studies on 1p/19q codeletion prediction in internal validation cohorts, with each point corresponding to a study.

Linear regression test of funnel plot asymmetry

Test result: t = 2.48, df = 12, p-value = 0.0287
Bias estimate: 2.1610 (SE = 0.8699)

Details:
- multiplicative residual heterogeneity variance (tau^2 = 3.7121)
- predictor: standard error
- weight: inverse variance

Applying Trim and Fill to assess publication bias

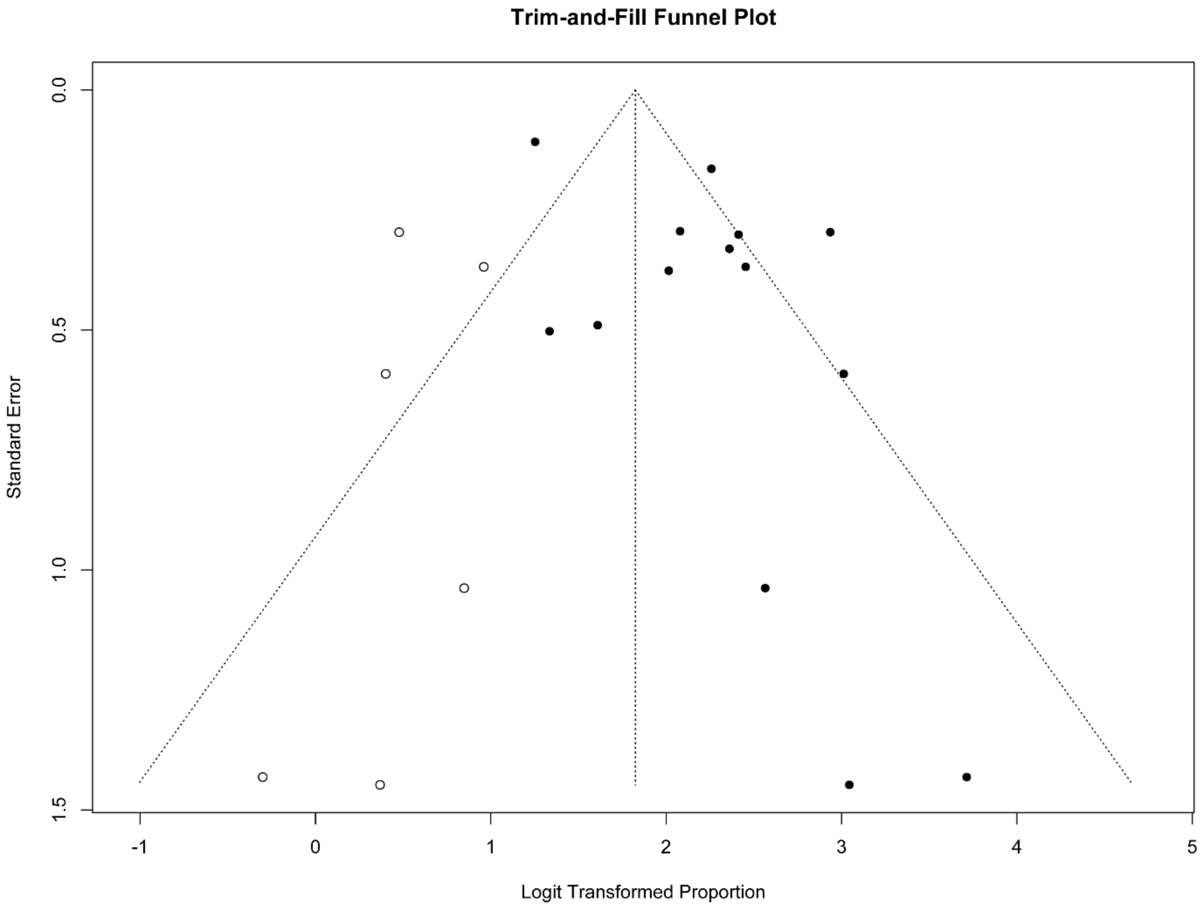

Figure 10. Funnel plot of studies reporting specificity for 1p/19q codeletion status prediction in the test cohorts after applying the Trim-and-Fill method. Adjustment for 6 imputed studies yielded a pooled specificity of 0.86 [95% CI: 0.81-0.90].

7.4. 1p/19q Codeletion Prediction in Test Cohorts

7.4.1. Pooled sensitivity estimate

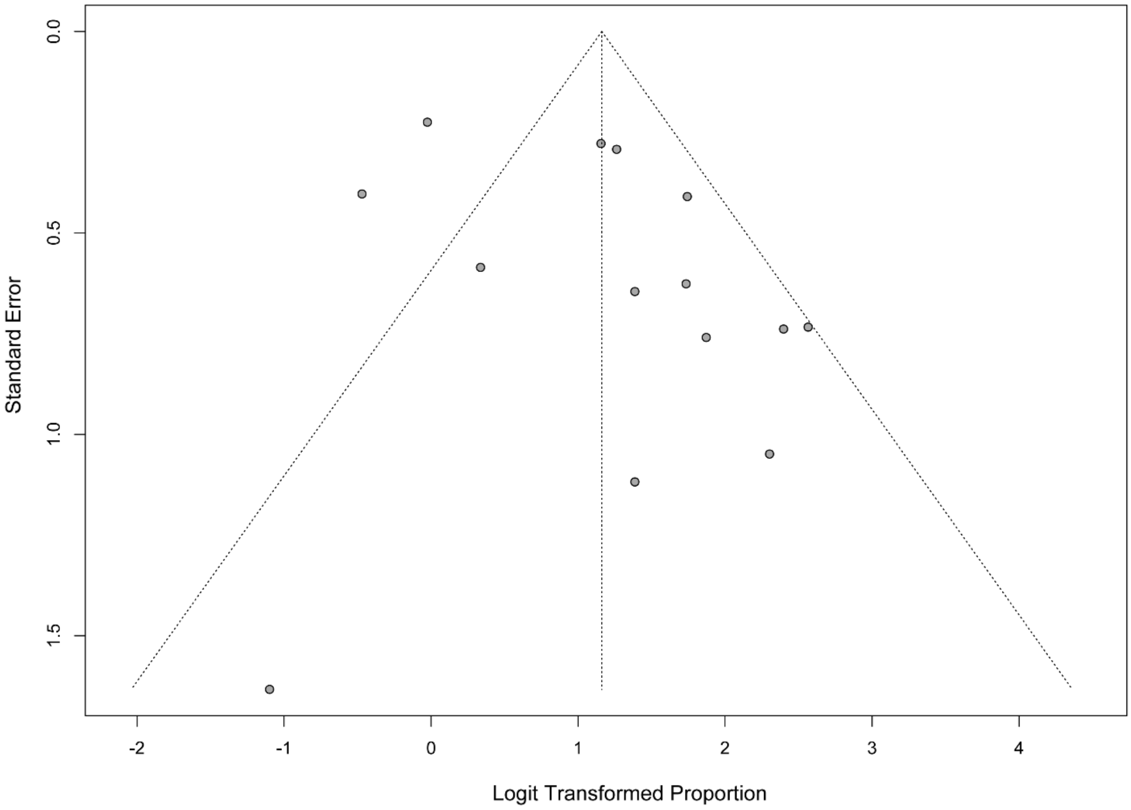

Figure 11. Funnel plot of the distribution of studies on 1p/19q codeletion prediction in test cohorts, with each point corresponding to a study.

Linear regression test of funnel plot asymmetry

Test result: t = 1.65, df = 13, p-value = 0.1229
Bias estimate: 1.5658 (SE = 0.9489)

Details:
- multiplicative residual heterogeneity variance (tau^2 = 3.3530)
- predictor: standard error
- weight: inverse variance
- reference: Egger et al. (1997), BMJ

7.4.2. Pooled specificity estimate

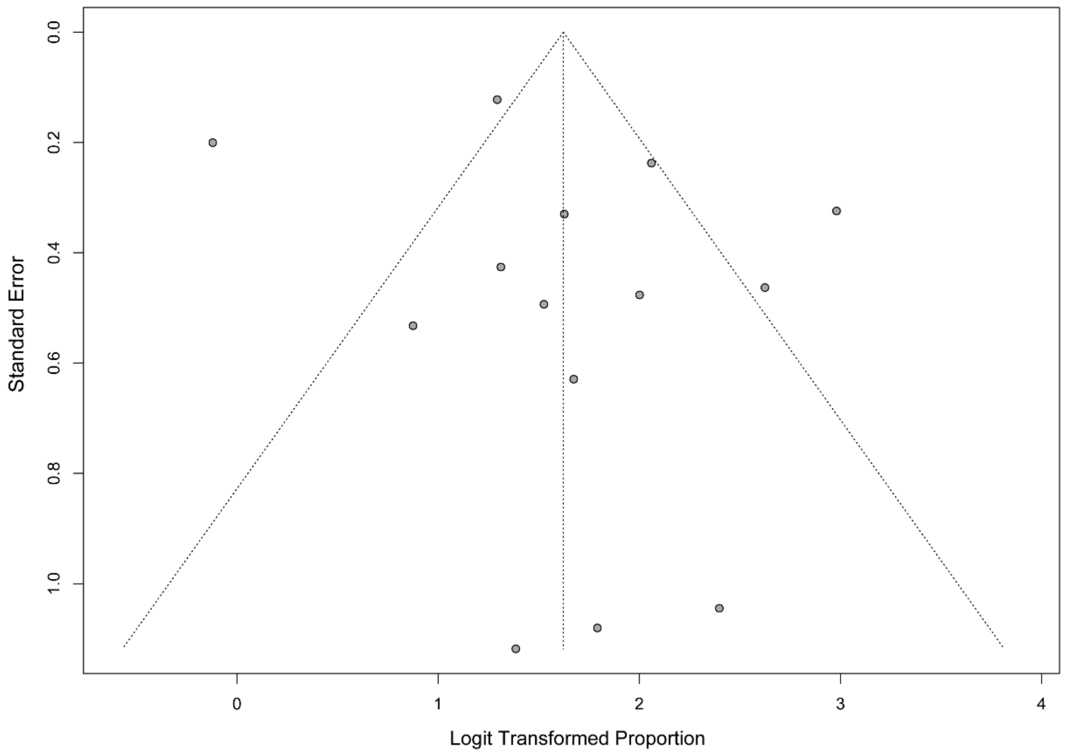

Figure 12. Funnel plot of the distribution of studies on 1p/19q codeletion prediction in test cohorts, with each point corresponding to a study.

Linear regression test of funnel plot asymmetry

Test result: t = 1.08, df = 13, p-value = 0.3009
Bias estimate: 1.2691 (SE = 1.1780)

Details:
- multiplicative residual heterogeneity variance (tau^2 = 7.1206)
- predictor: standard error
- weight: inverse variance
- reference: Egger et al. (1997), BMJ

8. Sensitivity analysis

8.1. IDH mutation Prediction in Internal Validation Datasets

8.1.1. Pooled sensitivity estimate

Identified outliers (random-effects model):

"McHugh H, et al. (2023) [34]", "Wei Y, et al. (2021) [47]", "Chen M, et al. (2024) [59]", "Li Y, et al. (2021) [69]", "Wu X, et al. (2024) [103]"

Results with outliers removed:

Number of studies: k = 38
Number of observations: o = 2786
Number of events: e = 2429

	proportion	95%-CI
Random effects model	0.8683	[0.8467; 0.8872]
Prediction interval		[0.7663; 0.9298]

Quantifying heterogeneity (with 95%-CIs):
tau^2 = 0.1106 [0.0174; 0.3102]; tau = 0.3326 [0.1318; 0.5570]
I^2 = 44.5% [18.6%; 62.2%]; H = 1.34 [1.11; 1.63]

Test of heterogeneity:

Q	d.f.	p-value
66.71	37	0.0020

Details of meta-analysis methods:
- Inverse variance method
- Restricted maximum-likelihood estimator for tau^2
- Q-Profile method for confidence interval of tau^2 and tau
- Calculation of I^2 based on Q
- Prediction interval based on t-distribution (df = 37)
- Logit transformation
- Continuity correction of 0.5 in studies with zero cell frequencies

8.1.2. Pooled specificity estimate

Identified outliers (random-effects model):

"Li Z, et al. (2017) [4]", "McHugh H, et al. (2023) [34]", "Zhang H, et al. (2023) [42]", "Zeng H, et al. (2022) [44]", "Xu Q, et al. (2022) [45]", "Lost J, et al. (2024) [70]", "Santinha J, et al. (2024) [75]", "Chen Q, et al. (2025) [104]"

Results with outliers removed:

Number of studies: k = 35
Number of observations: o = 3691
Number of events: e = 3312

	**proportion**	**95%-CI**
Random effects model	0.8946	[0.8794; 0.9080]
Prediction interval		[0.8335; 0.9350]

Quantifying heterogeneity (with 95%-CIs):
tau^2 = 0.0615 [0.0013; 0.2182]; tau = 0.2480 [0.0364; 0.4671]
I^2 = 35.9% [3.2%; 57.5%]; H = 1.25 [1.02; 1.53]

Test of heterogeneity:

Q	d.f.	p-value
53.03	34	0.0198

Details of meta-analysis methods:
- Inverse variance method
- Restricted maximum-likelihood estimator for tau^2
- Q-Profile method for confidence interval of tau^2 and tau
- Calculation of I^2 based on Q
- Prediction interval based on t-distribution (df = 34)
- Logit transformation
- Continuity correction of 0.5 in studies with zero cell frequencies

8.2. IDH mutation Prediction in Test Cohorts

8.2.1. Pooled sensitivity estimate

Identified outliers (random-effects model):
"Chakrabarty S, et al. (2023) [23]", "Chen M, et al. (2024) [59]", "Gómez Vecchio T, et al. (2024) [63]", "Nishikawa T, et al. (2023) [71]", "Chen Q, et al. (2025) [104]"

Results with outliers removed:

Number of studies: k = 47
Number of observations: o = 2203
Number of events: e = 1797

	proportion	95%-CI
Random effects model	0.8080	[0.7874; 0.8271]
Prediction interval		[0.7471; 0.8571]

Quantifying heterogeneity (with 95%-CIs):
tau^2 = 0.0267 [0.0000; 0.1764]; tau = 0.1634 [0.0000; 0.4199]
I^2 = 14.7% [0.0%; 41.0%]; H = 1.08 [1.00; 1.30]

Test of heterogeneity:

Q	d.f.	p-value
53.95	46	0.1965

Details of meta-analysis methods:
- Inverse variance method
- Restricted maximum-likelihood estimator for tau^2
- Q-Profile method for confidence interval of tau^2 and tau
- Calculation of I^2 based on Q
- Prediction interval based on t-distribution (df = 46)
- Logit transformation
- Continuity correction of 0.5 in studies with zero cell frequencies

8.2.2. Pooled specificity estimate

Identified outliers (random-effects model):
"Ge C, et al. (2020) [3]", "Ali MB, et al. (2020) [9]", "Decuyper M, et al. (2021) [11]", "Calabrese E, et al. (2020) [19]", "Chakrabarty S, et al. (2023) [23]", "McHugh H, et al. (2023) [34]", "Yogananda CGB, et al. (2023) [43]", "Kihira S, et al. (2022) [55]", "Chen Q, et al. (2025) [104]"

Results with outliers removed:

Number of studies: k = 43
Number of observations: o = 3389
Number of events: e = 2898

	**proportion**	**95%-CI**
Random effects model	0.8511	[0.8326; 0.8679]
Prediction interval		[0.7707; 0.9067]

Quantifying heterogeneity (with 95%-CIs):
tau^2 = 0.0641 [0.0097; 0.2729]; tau = 0.2532 [0.0983; 0.5224]
I^2 = 39.3% [12.3%; 58.0%]; H = 1.28 [1.07; 1.54]

Test of heterogeneity:

Q	d.f.	p-value
69.21	42	0.0051

Details of meta-analysis methods:
- Inverse variance method
- Restricted maximum-likelihood estimator for tau^2
- Q-Profile method for confidence interval of tau^2 and tau
- Calculation of I^2 based on Q
- Prediction interval based on t-distribution (df = 42)
- Logit transformation
- Continuity correction of 0.5 in studies with zero cell frequencies

8.3. 1p/19q Codeletion Prediction in Internal Validation Datasets

8.3.1. Pooled sensitivity estimate

Identified outliers (random-effects model):
"Li Y, et al. (2021) [69]"

Results with outliers removed:

Number of studies: k = 13
Number of observations: o = 681
Number of events: e = 566

	proportion	95%-CI
Random effects model	0.8124	[0.7262; 0.8761]
Prediction interval		[0.4500; 0.9582]

Quantifying heterogeneity (with 95%-CIs):
tau^2 = 0.5225 [0.1617; 2.3856]; tau = 0.7228 [0.4022; 1.5445]
I^2 = 74.2% [55.4%; 85.1%]; H = 1.97 [1.50; 2.59]

Test of heterogeneity:

Q	d.f.	p-value
46.56	12	< 0.0001

Details of meta-analysis methods:
- Inverse variance method
- Restricted maximum-likelihood estimator for tau^2
- Q-Profile method for confidence interval of tau^2 and tau
- Calculation of I^2 based on Q
- Prediction interval based on t-distribution (df = 12)
- Logit transformation
- Continuity correction of 0.5 in studies with zero cell frequencies

8.3.2. Pooled specificity estimate

Identified outliers (random-effects model):
"Wu X et al, 2024 [103]"

Results with outliers removed:

Number of studies: k = 13
Number of observations: o = 1381
Number of events: e = 1263

	proportion	95%-CI
Random effects model	0.9095	[0.8905; 0.9255]
Prediction interval		[0.8750; 0.9352]

Quantifying heterogeneity (with 95%-CIs):
tau^2 = 0.0159 [0.0000; 0.5145]; tau = 0.1259 [0.0000; 0.7173]
I^2 = 17.7% [0.0%; 56.2%]; H = 1.10 [1.00; 1.51]

Test of heterogeneity:

Q	d.f.	p-value
14.58	12	0.2653

Details of meta-analysis methods:
- Inverse variance method
- Restricted maximum-likelihood estimator for tau^2
- Q-Profile method for confidence interval of tau^2 and tau
- Calculation of I^2 based on Q
- Prediction interval based on t-distribution (df = 12)
- Logit transformation
- Continuity correction of 0.5 in studies with zero cell frequencies

8.4. 1p/19q Codeletion Prediction in Test Cohorts

8.4.1. Pooled sensitivity estimate

Identified outliers (random-effects model)

"van der Voort SR, et al. (2023) [13]", "Nishikawa T, et al. (2023) [71]"

Results with outliers removed

Number of studies: k = 13
Number of observations: o = 322
Number of events: e = 262

	**proportion**	**95%-CI**
Random effects model	0.8010	[0.7514; 0.8428]
Prediction interval		[0.7454; 0.8470]

Quantifying heterogeneity (with 95%-CIs):
$tau^2 < 0.0001$ [0.0000; 1.1632]; tau = 0.0019 [0.0000; 1.0785]
$I^2 = 8.3\%$ [0.0%; 46.1%]; H = 1.04 [1.00; 1.36]

Test of heterogeneity:

Q	d.f.	p-value
13.09	12	0.3625

Details of meta-analysis methods:
- Inverse variance method
- Restricted maximum-likelihood estimator for tau^2
- Q-Profile method for confidence interval of tau^2 and tau
- Calculation of I^2 based on Q
- Prediction interval based on t-distribution (df = 12)

8.4.2. Pooled specificity estimate

Identified outliers (random-effects model):

"van der Voort SR, et al. (2023) [13]", "Farahani S, et al. (2025) [101]"

Results with outliers removed:

Number of studies: k = 13
Number of observations: o = 881
Number of events: e = 731

	proportion	95%-CI
Random effects model	0.8400	[0.7951; 0.8765]
Prediction interval		[0.7148; 0.9166]

Quantifying heterogeneity (with 95%-CIs):
tau^2 = 0.0913 [0.0000; 0.3393]; tau = 0.3021 [0.0000; 0.5825]
I^2 = 33.2% [0.0%; 65.5%]; H = 1.22 [1.00; 1.70]

Test of heterogeneity:

Q	d.f.	p-value
17.97	12	0.1166

Details of meta-analysis methods:
- Inverse variance method
- Restricted maximum-likelihood estimator for tau^2
- Q-Profile method for confidence interval of tau^2 and tau
- Calculation of I^2 based on Q
- Prediction interval based on t-distribution (df = 12)
- Logit transformation

9. Forest plot

9.1. IDH Prediction in Internal Validation Sets

9.1.1. Pooled sensitivity estimate (before applying the Duval & Tweedie Trim-and-Fill method)

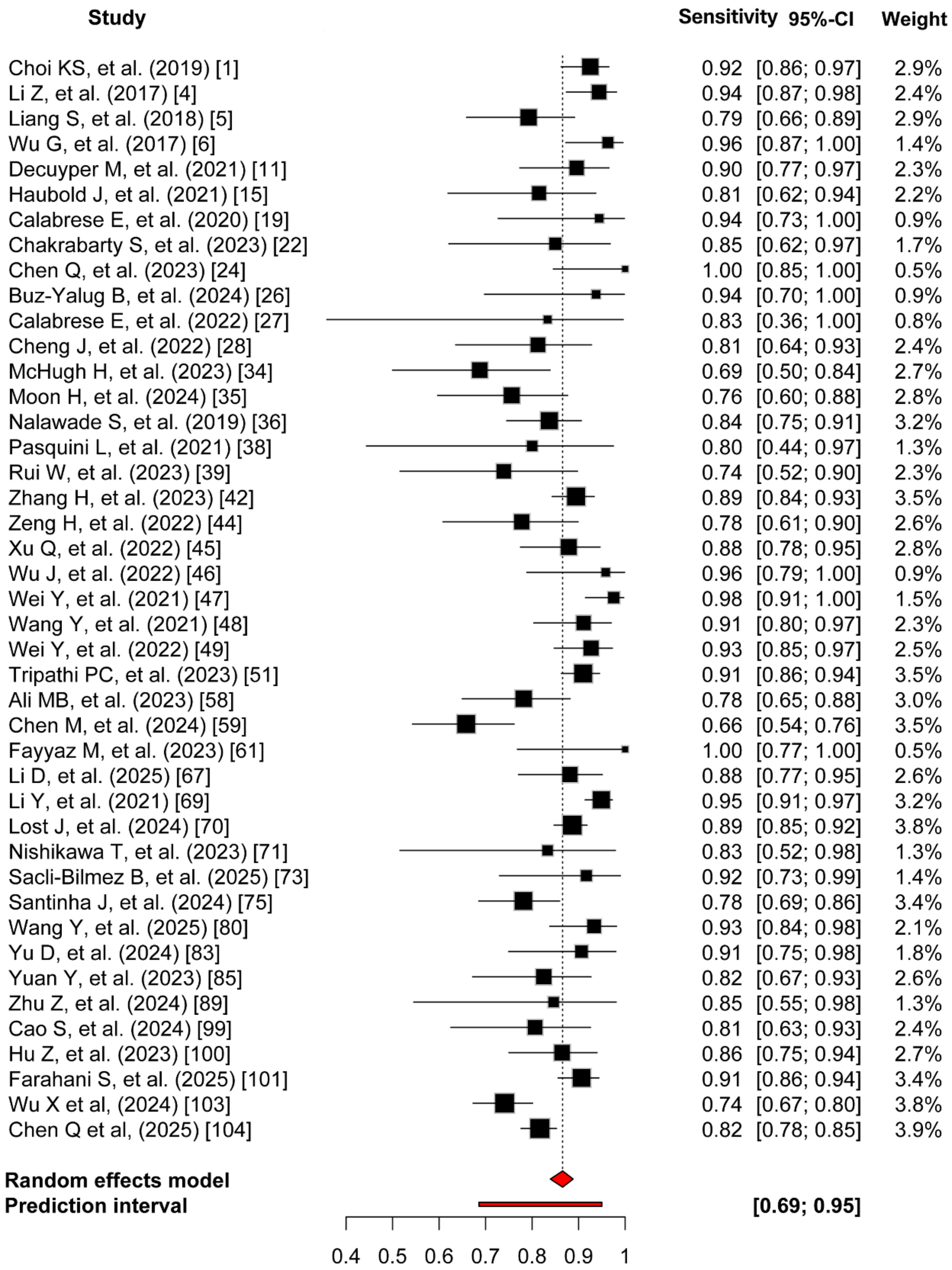

Figure 13. Forest plot visualization of internal validation sets for sensitivity of IDH status prediction. The prediction interval estimate under a random effects model is depicted at the bottom of the plot. **Abbreviations**: IDH, isocitrate dehydrogenase; CI, confidence interval.

9.1.2. Pooled sensitivity estimate (after applying the Duval & Tweedie Trim-and-Fill method)

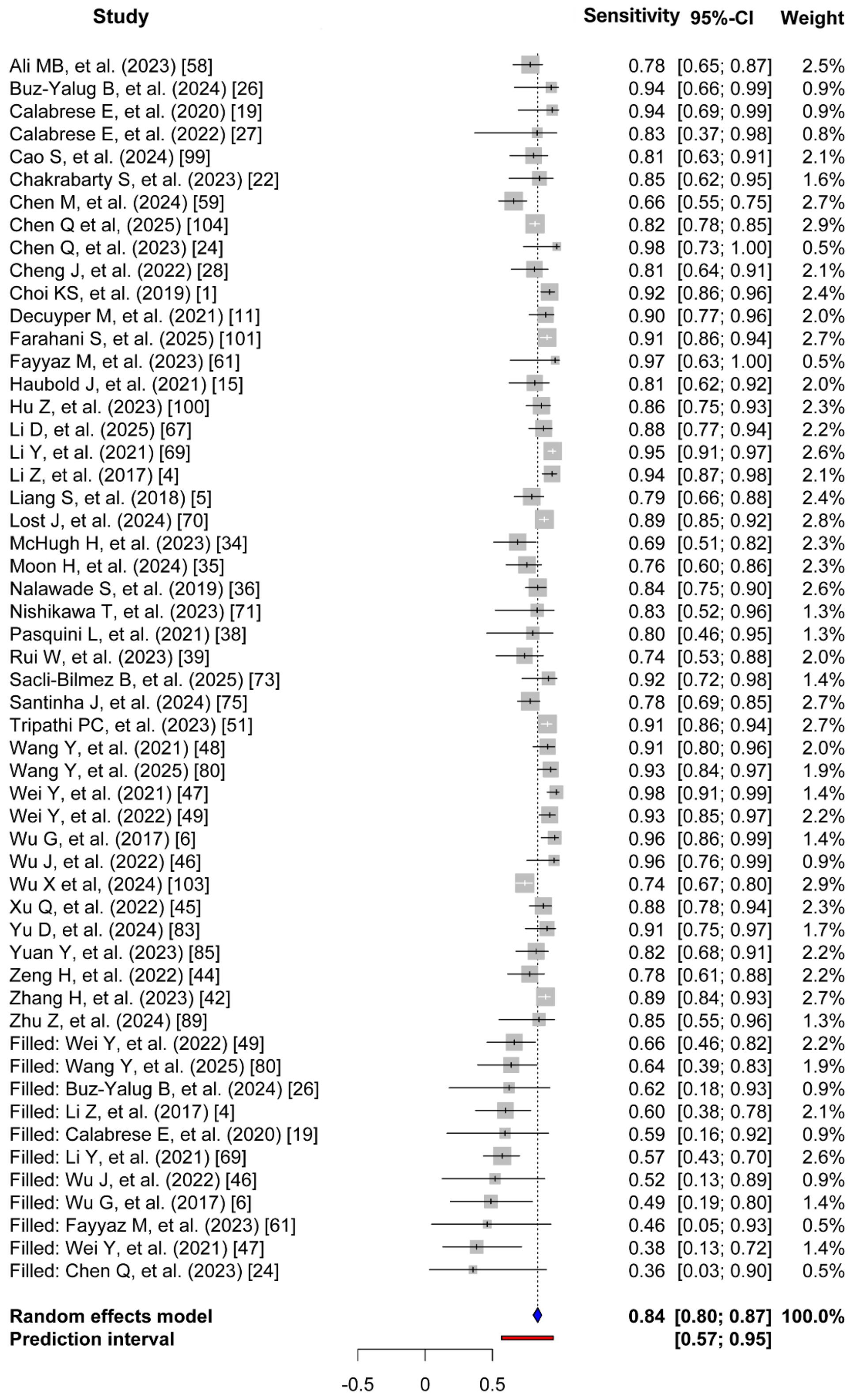

Figure 14. Forest plot of internal validation sets showing sensitivity of IDH status prediction after applying the Duval & Tweedie Trim-and-Fill method, including original and imputed studies. The prediction interval under a random-effects model is shown. **Abbreviations**: IDH, isocitrate dehydrogenase; CI, confidence interval.

9.1.3. Pooled specificity estimate (before applying the Duval & Tweedie Trim-and-Fill method)

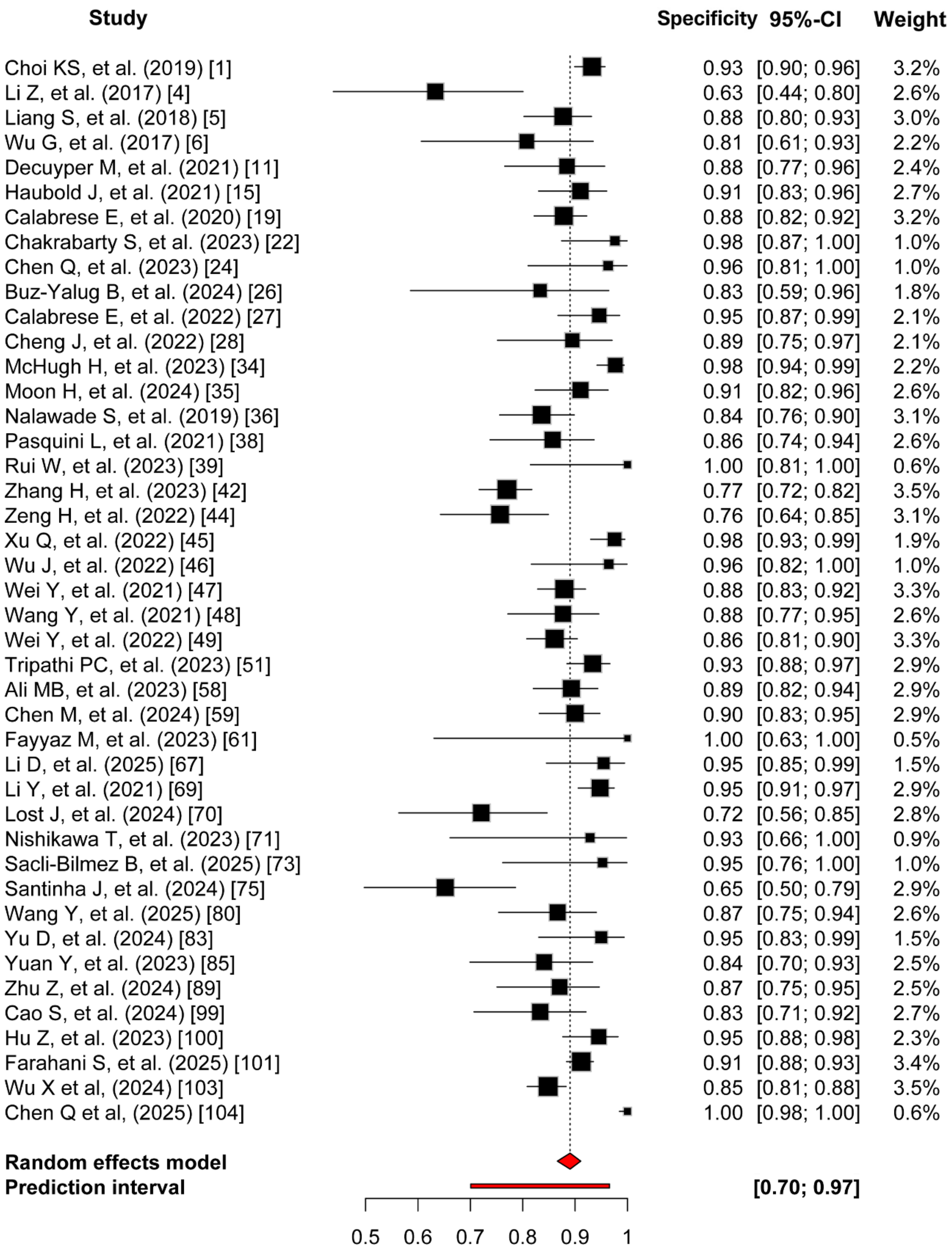

Figure 15. Forest plot visualization of internal validations sets for specificity of IDH status prediction. The prediction interval under a random effects model is depicted at the bottom of the plot. **Abbreviations**: IDH for isocitrate dehydrogenase, CI for confidence interval.

9.1.4. Pooled specificity estimate (after applying the Duval & Tweedie Trim-and-Fill method)

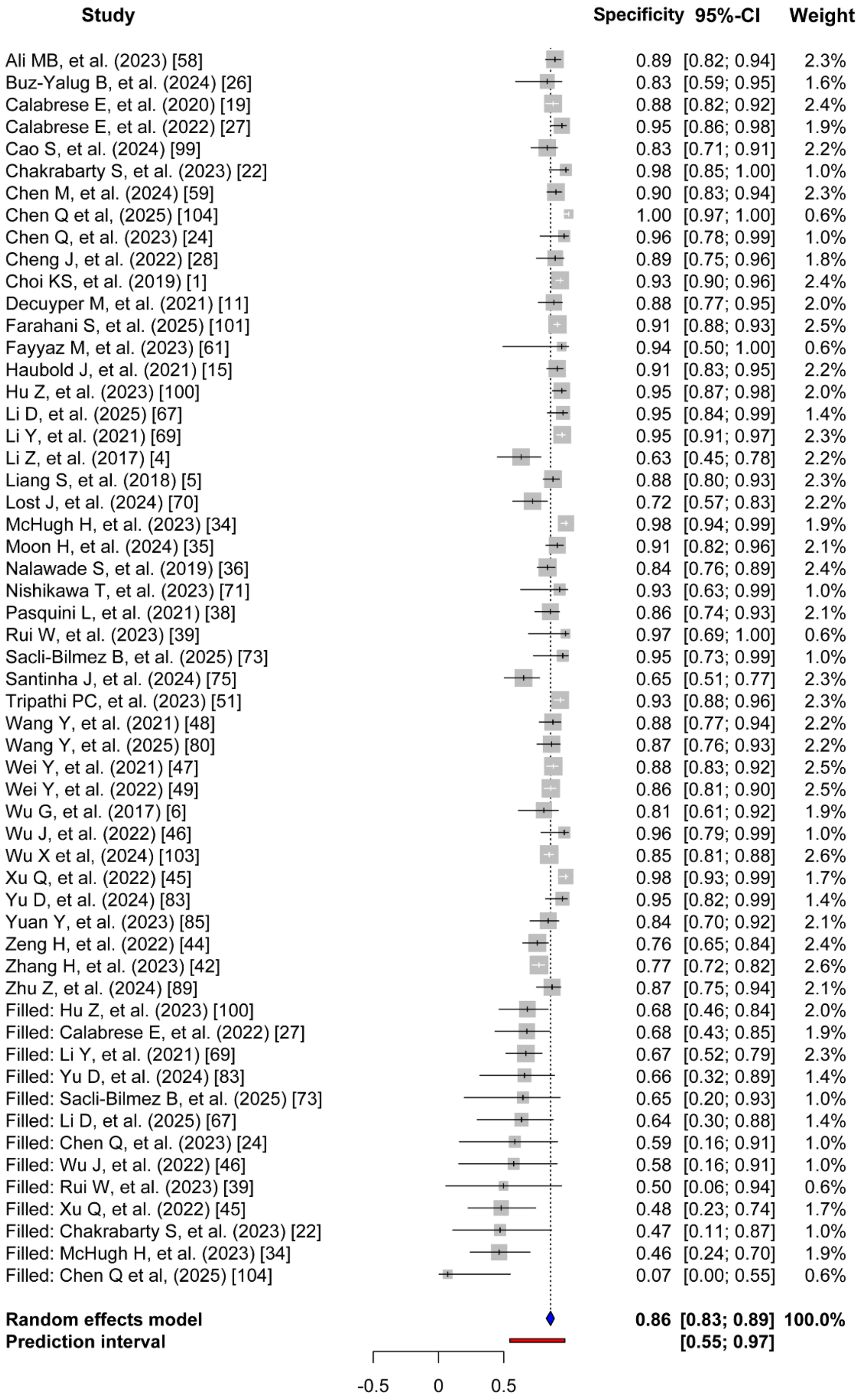

Figure 16. Forest plot of internal validation sets showing specificity of IDH status prediction after applying the Duval & Tweedie Trim-and-Fill method, including original and imputed studies. The prediction interval under a random-effects model is shown. **Abbreviations**: IDH, isocitrate dehydrogenase; CI, confidence interval.

9.2. IDH Prediction in Test Cohorts

9.2.1. Pooled sensitivity estimate (after applying the Duval & Tweedie Trim-and-Fill method)

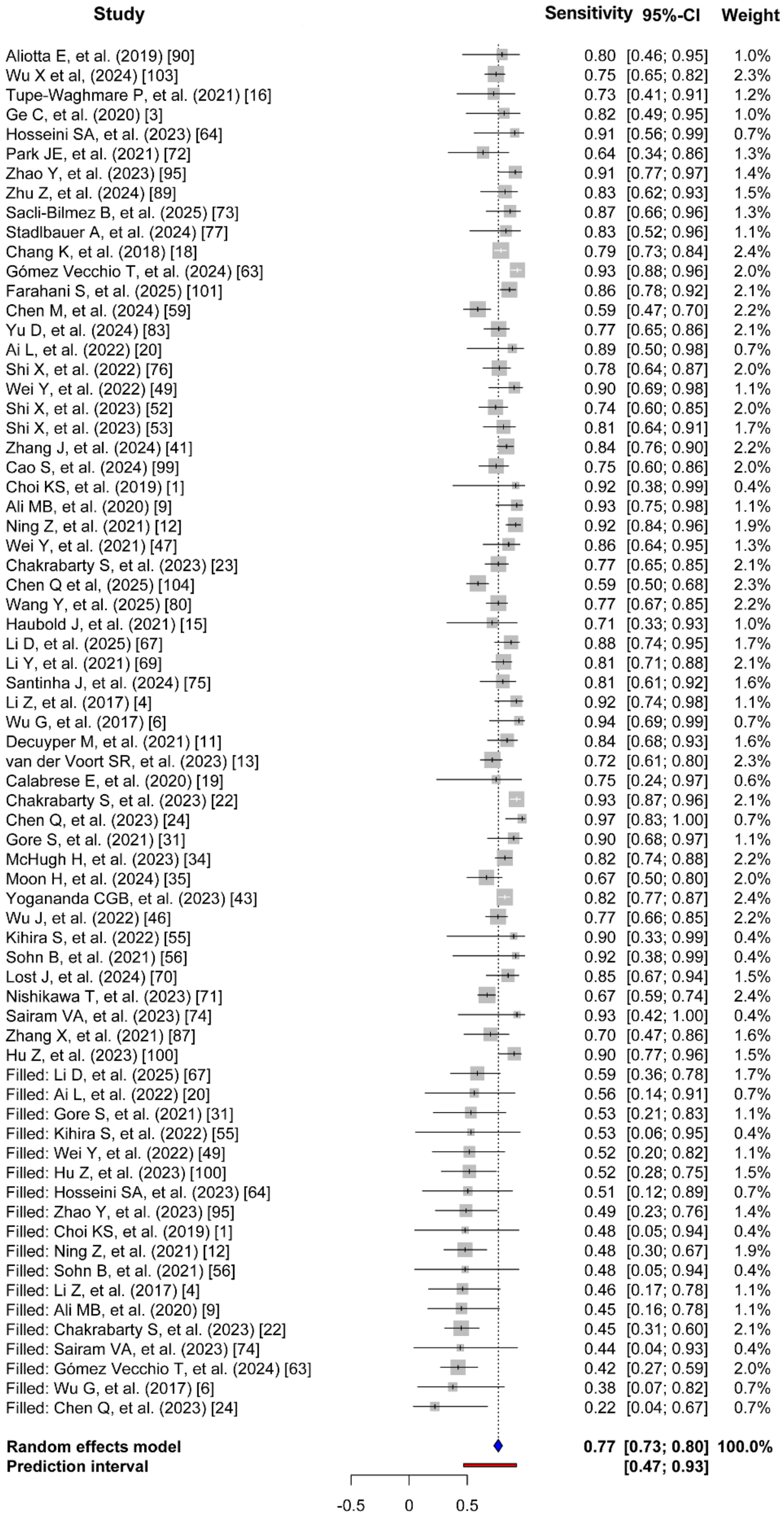

Figure 17. Forest plot of test sets showing sensitivity of IDH status prediction after applying the Duval & Tweedie Trim-and-Fill method, including original and imputed studies. The prediction interval under a random-effects model is shown. **Abbreviations**: IDH, isocitrate dehydrogenase; CI, confidence interval.

9.3. 1p/19q Codeletion Prediction in Internal Validation Datasets

9.3.1. Pooled sensitivity estimate

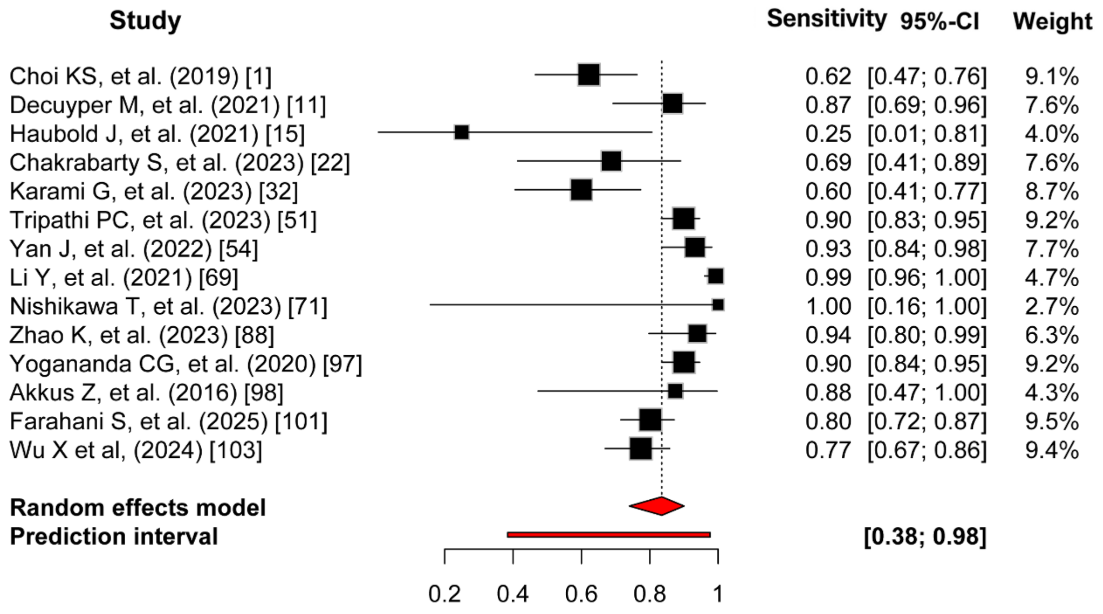

Figure 18. Forest plot visualization of internal validation sets for sensitivity of 1p/19q codeletion prediction. The prediction interval under a random effects model is depicted at the bottom of the plot. **Abbreviation**: CI for confidence interval.

9.3.2. Pooled specificity estimate

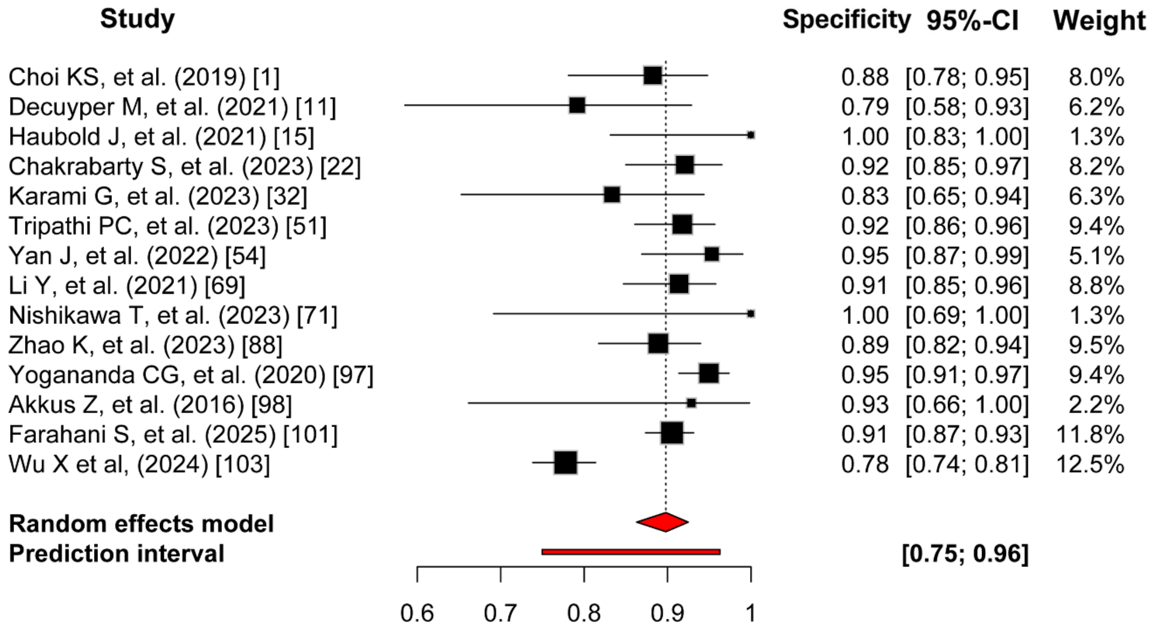

Figure 19. Forest plot visualization of internal validation datasets for specificity of 1p/19q codeletion prediction. The prediction interval under a random effects model is depicted at the bottom of the plot. **Abbreviation**: CI for confidence interval.

9.3.3. Pooled specificity estimate (after applying the Duval & Tweedie Trim-and-Fill method)

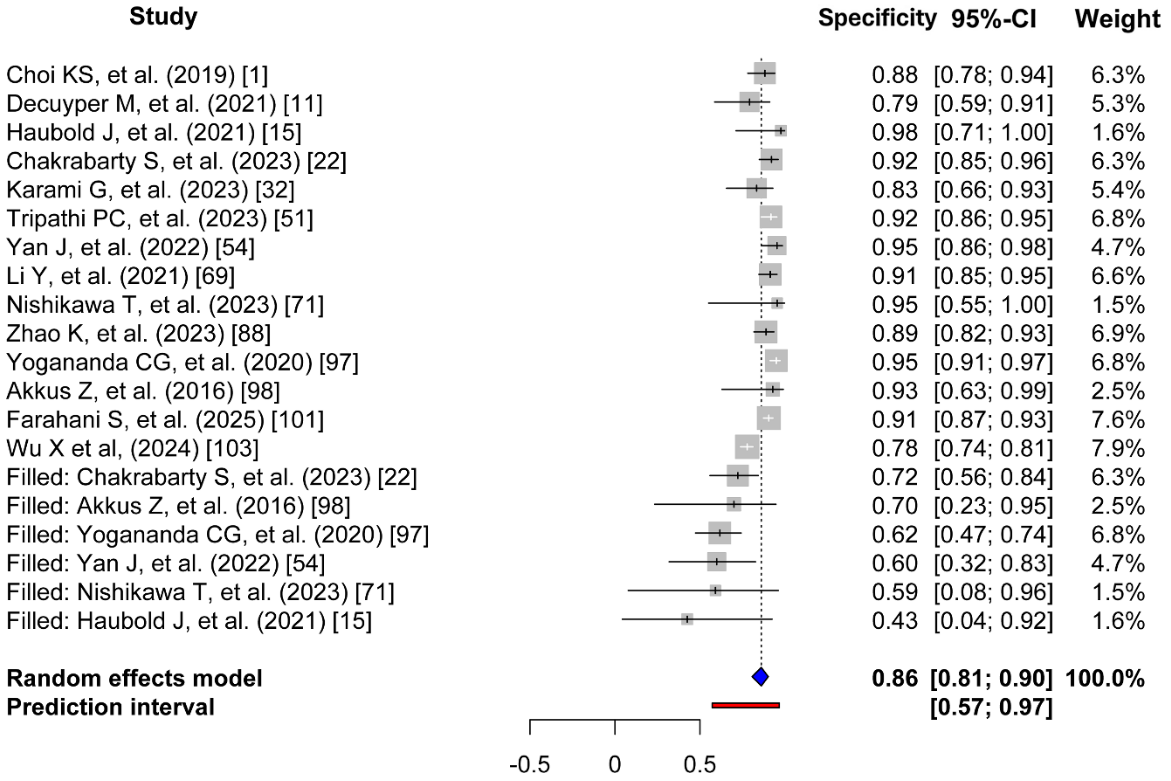

Figure 20. Forest plot of test sets showing specificity of 1p/19q codeletion status prediction after applying the Duval & Tweedie Trim-and-Fill method, including original and imputed studies. The pooled estimate and prediction intervals under a random-effects model are shown. **Abbreviation**: CI, confidence interval.

10. Cross-Hair Plot

10.1. IDH Prediction in Internal Validation Sets

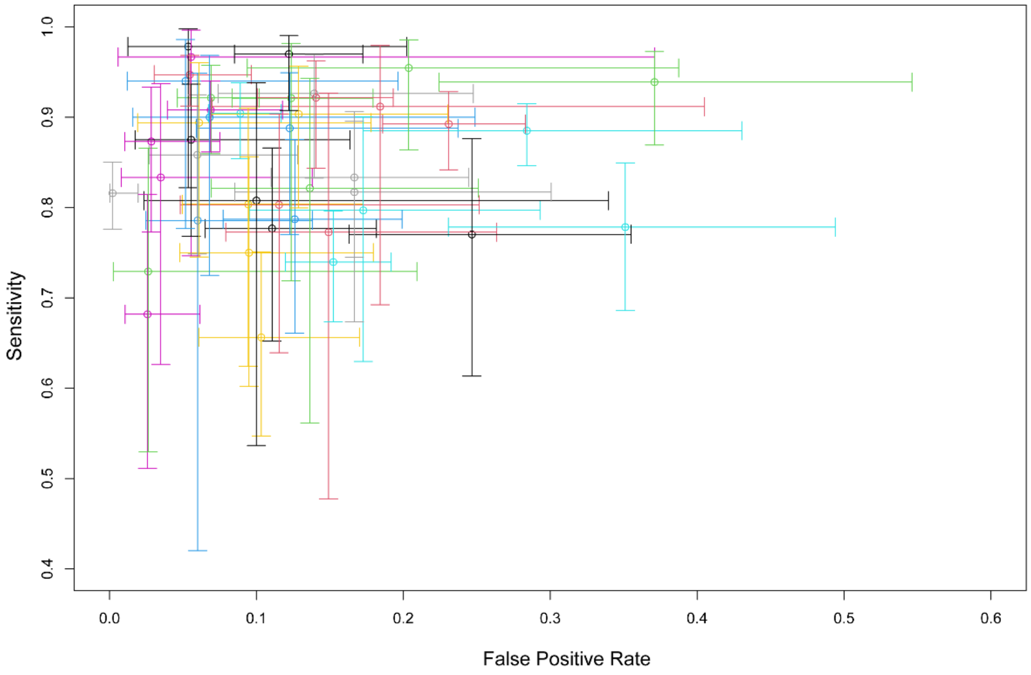

Figure 21. Cross-Hair plot of IDH prediction in internal validation sets. The plot displays individual studies in ROC space, with cross-hairs representing the confidence intervals for sensitivity and specificity.

10.2. IDH Prediction in Test Cohorts

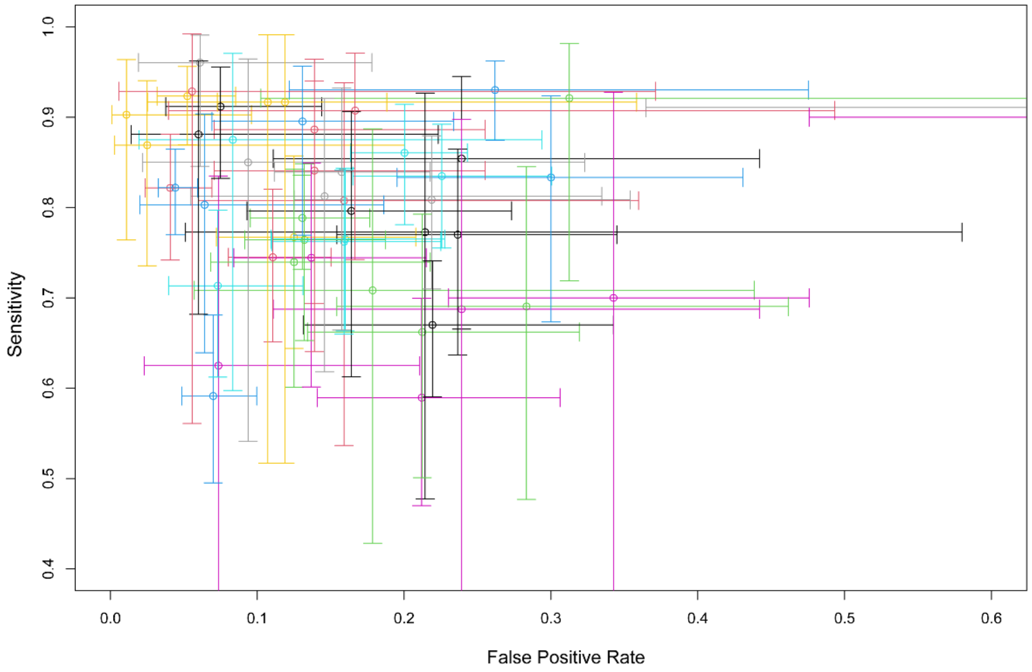

Figure 22. Cross-Hair Plot of IDH prediction in test cohorts. The plot displays individual studies in ROC space, with cross-hairs representing the confidence intervals for sensitivity and specificity.

10.3. 1p/19q Codeletion Prediction in Internal Validation Cohorts

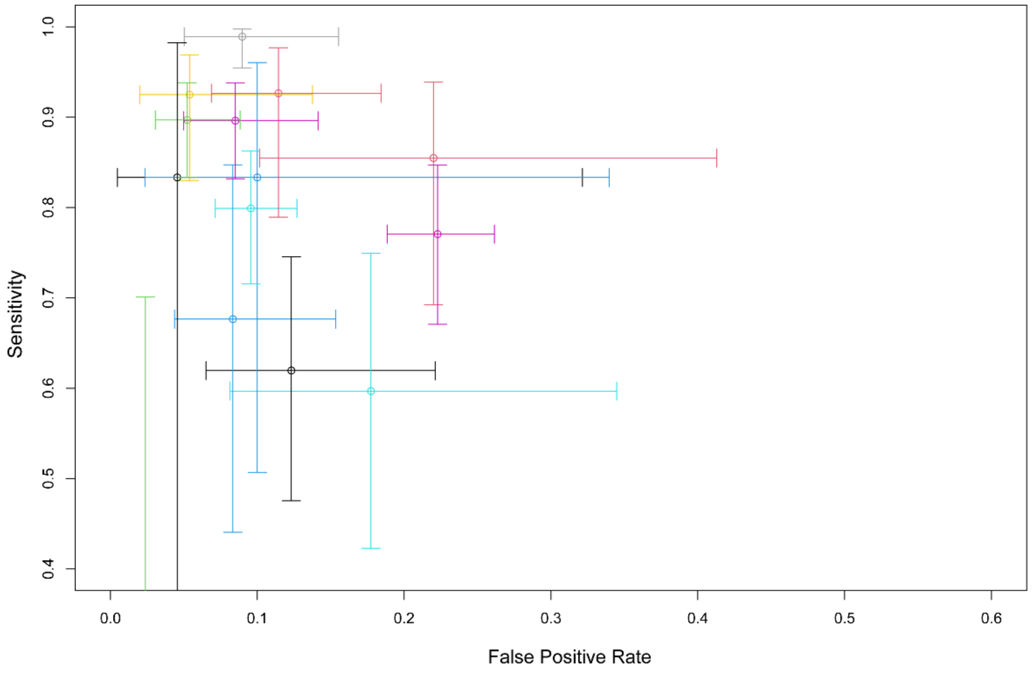

Figure 23. Cross-Hair Plot of 1p/9q codeletion prediction in internal validation cohorts. The plot displays individual studies in ROC space, with cross-hairs representing the confidence intervals for sensitivity and specificity.

10.4. 1p/19q Codeletion Prediction in Test Cohorts

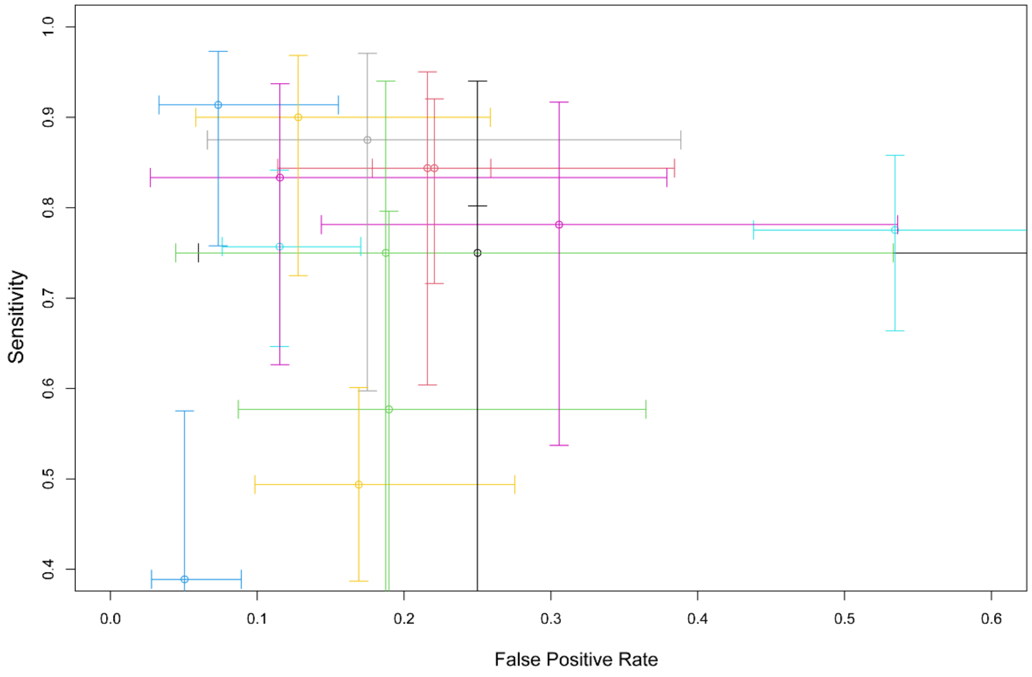

Figure 24. Cross-Hair Plot of 1p/9q codeletion prediction in test cohorts. The plot displays individual studies in ROC space, with cross-hairs representing the confidence intervals for sensitivity and specificity.

11. Statistical Power

11.1. IDH Prediction in Internal Validation Datasets

11.1.1. Pooled sensitivity estimate

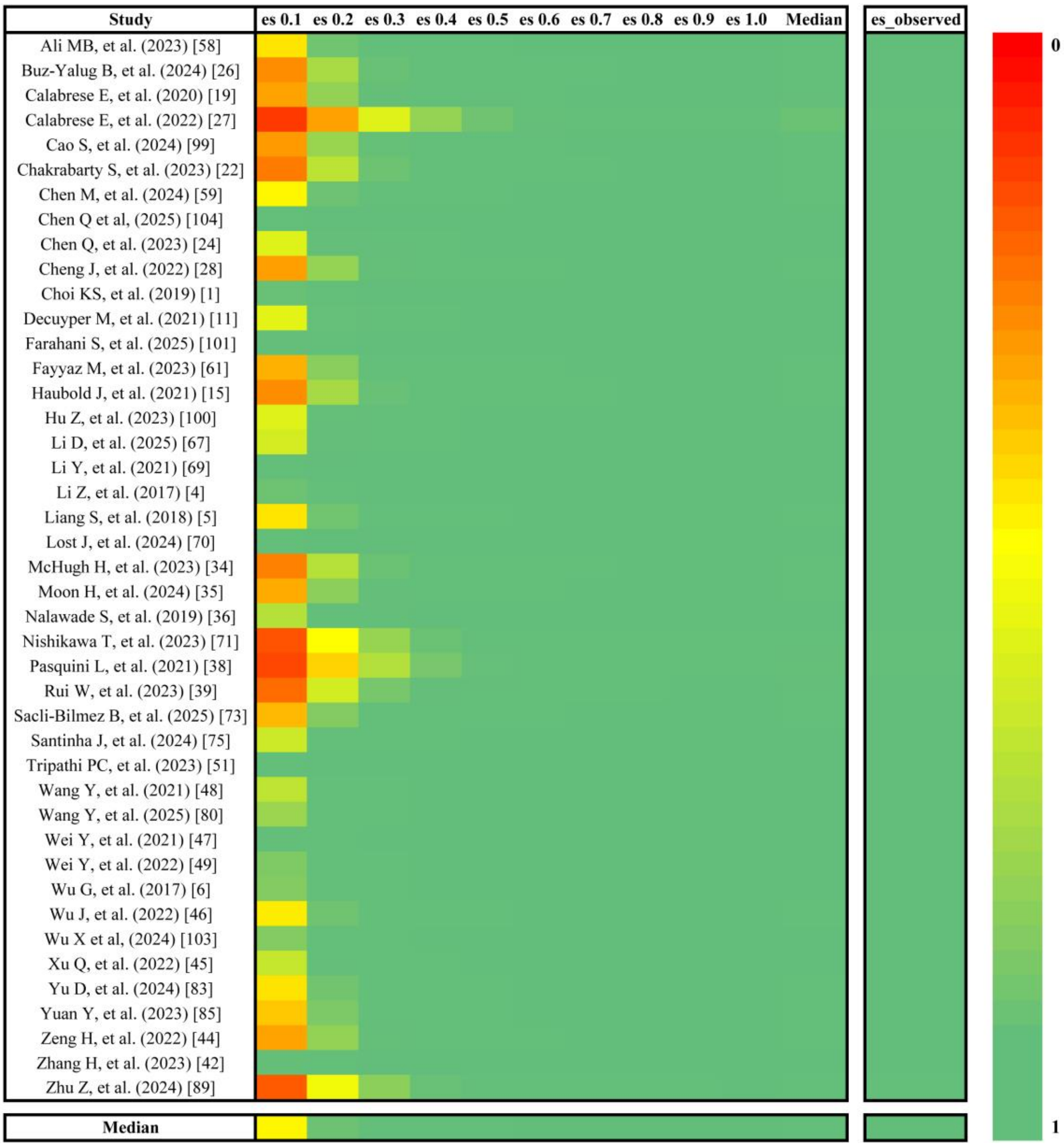

Figure 25. Heatmap of statistical power for IDH prediction in internal validation groups, indicating a high detection capability for larger sensitivities in included studies but relatively lower power for detecting smaller sensitivity measures in some studies. Greener areas represent higher statistical power for corresponding effect sizes (see color bar). **Abbreviations:** IDH, isocitrate dehydrogenase; es, effect size.

11.1.2. Pooled specificity estimate

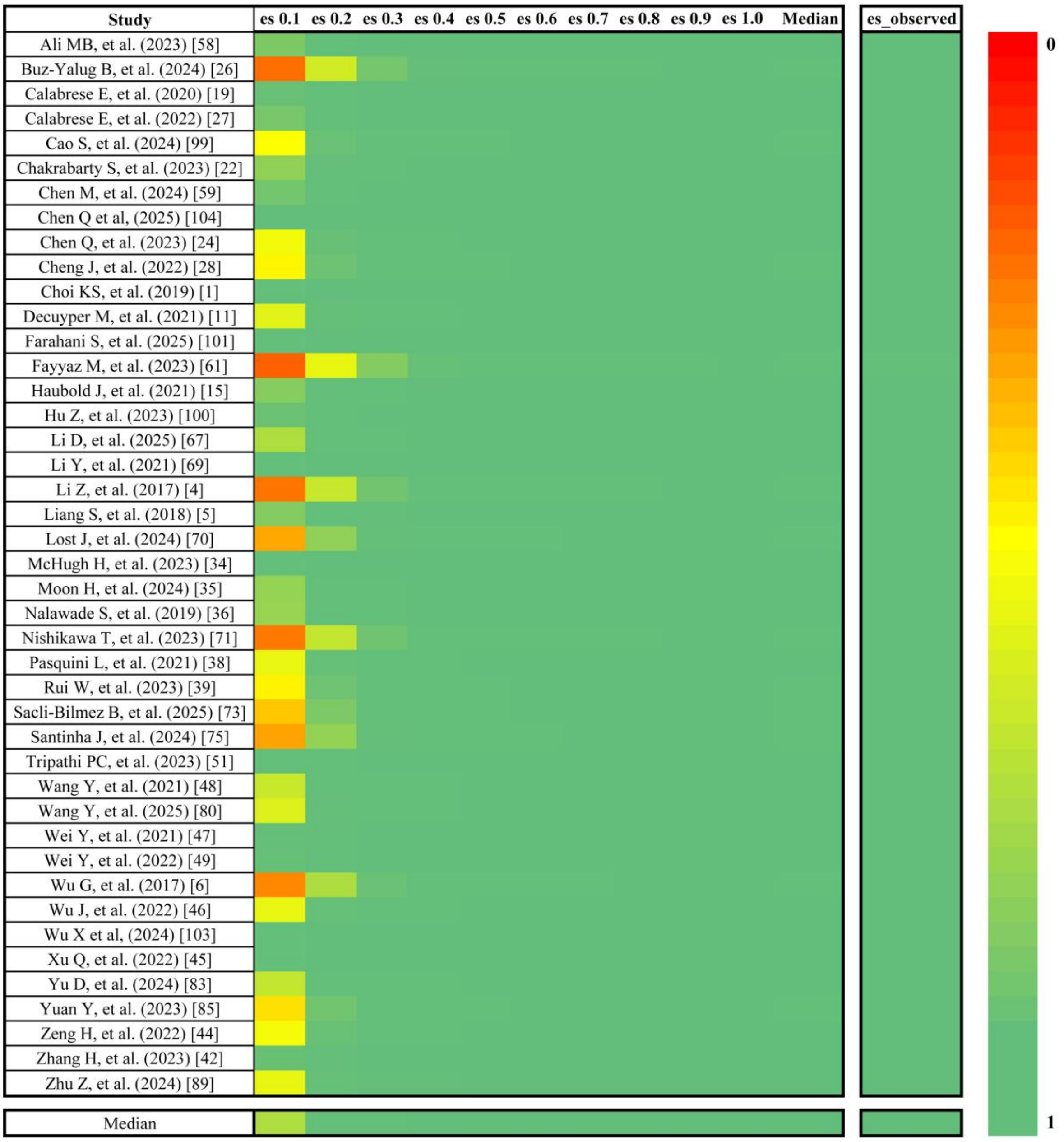

Figure 26. Heatmap of statistical power for IDH prediction in validation groups, indicating a high detection capability for larger specificities in included studies but relatively lower power for detecting smaller specificity measures in some studies. Greener areas represent higher statistical power for corresponding effect sizes (see color bar). **Abbreviations:** IDH, isocitrate dehydrogenase; es, effect size.

11.2. IDH Prediction in Test Cohorts

11.2.1. Pooled sensitivity estimate

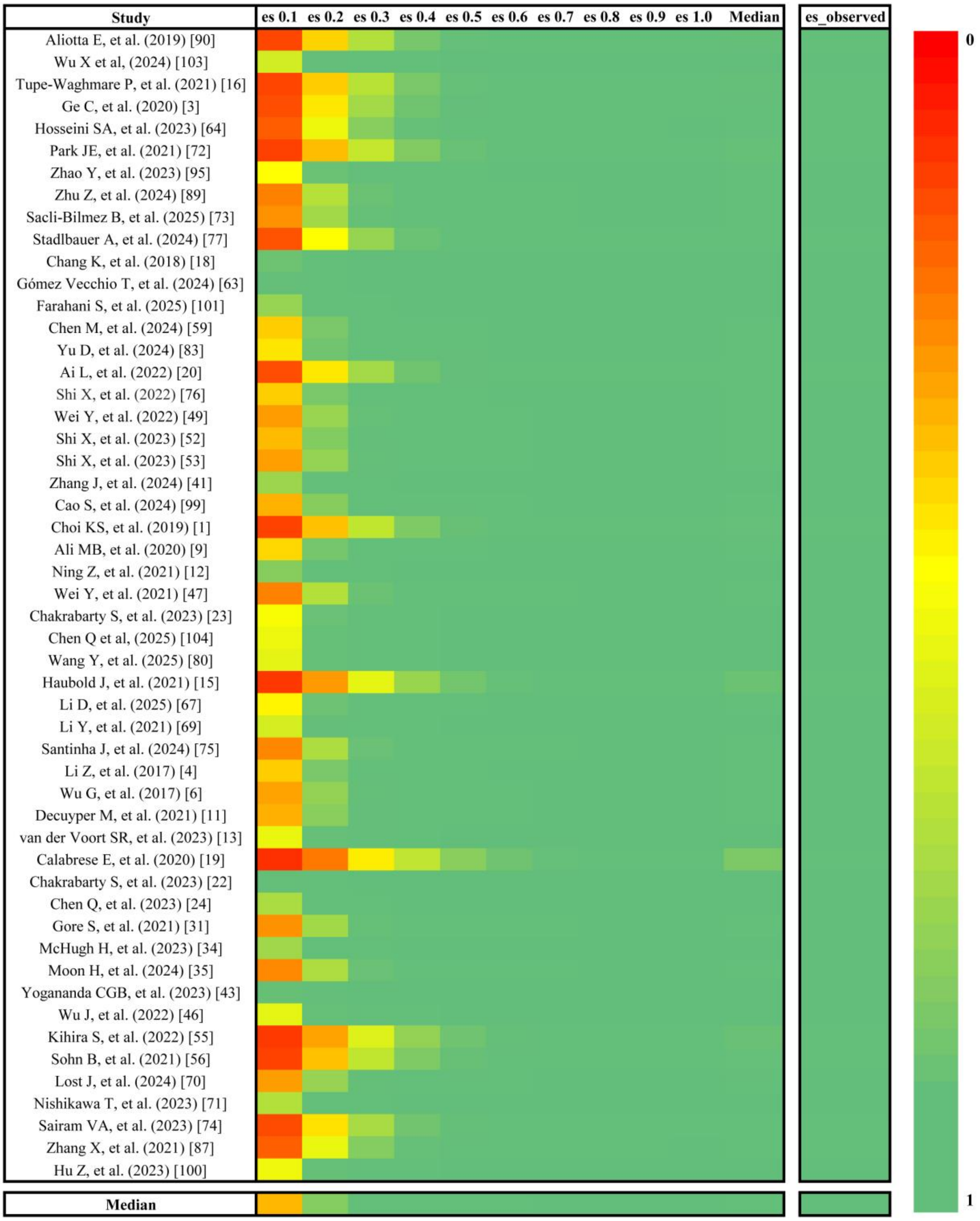

Figure 27. Heatmap of statistical power for IDH prediction in test groups, indicating a high detection capability for larger sensitivities in included studies but relatively lower power for detecting smaller sensitivity measures in some studies. Greener areas represent higher statistical power for corresponding effect sizes (see color bar). **Abbreviations:** IDH, isocitrate dehydrogenase; es, effect size.

11.2.2. Pooled specificity estimate

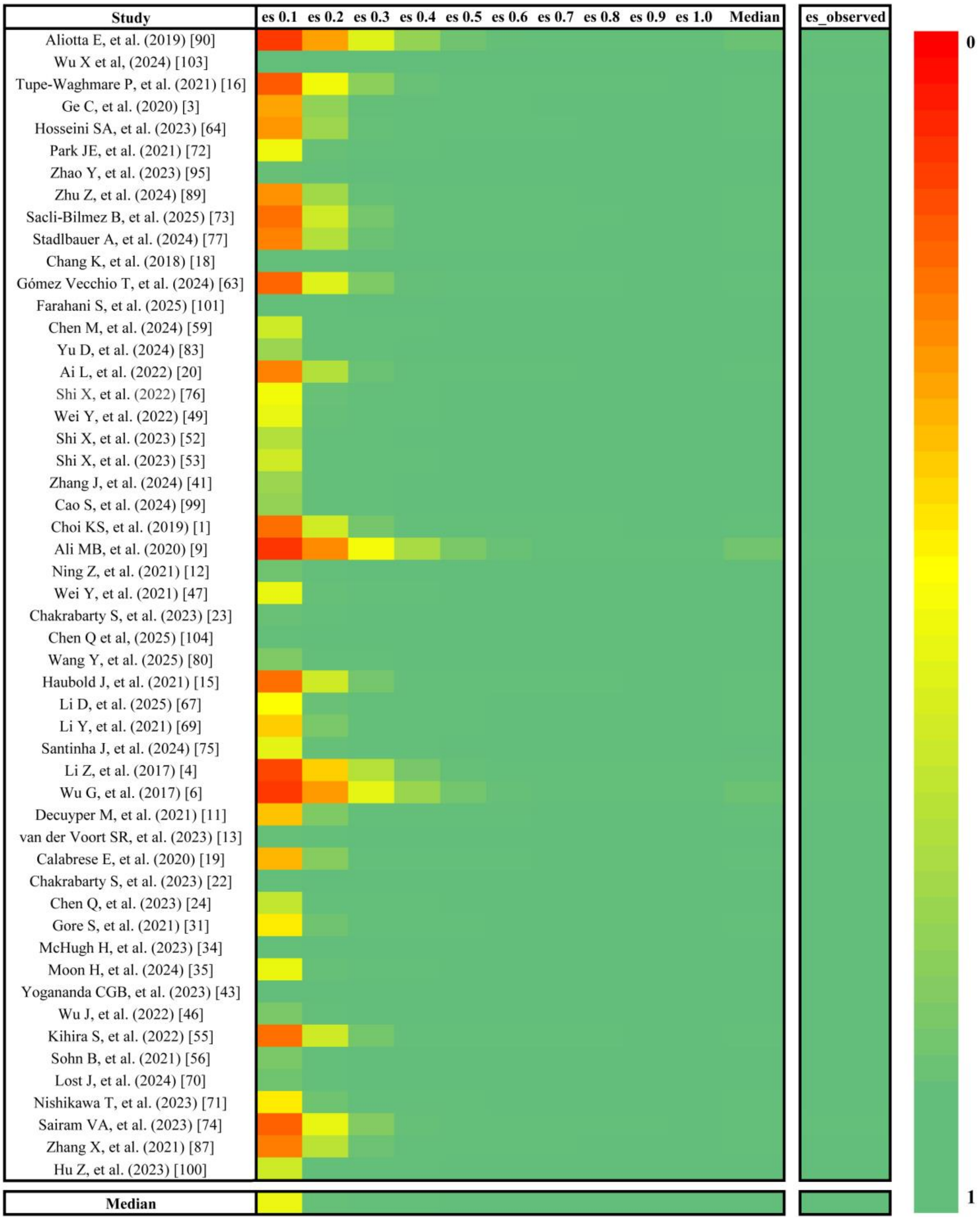

Figure 28. Heatmap of statistical power for IDH prediction in internal validation groups, indicating a high detection capability for larger specificities in included studies but relatively lower power for detecting smaller specificity measures in some studies. Greener areas represent higher statistical power for corresponding effect sizes (see color bar). **Abbreviations:** IDH, isocitrate dehydrogenase; es, effect size.

11.3. 1p/19q Codeletion Prediction in Internal Validation Sets

11.3.1. Pooled sensitivity estimate

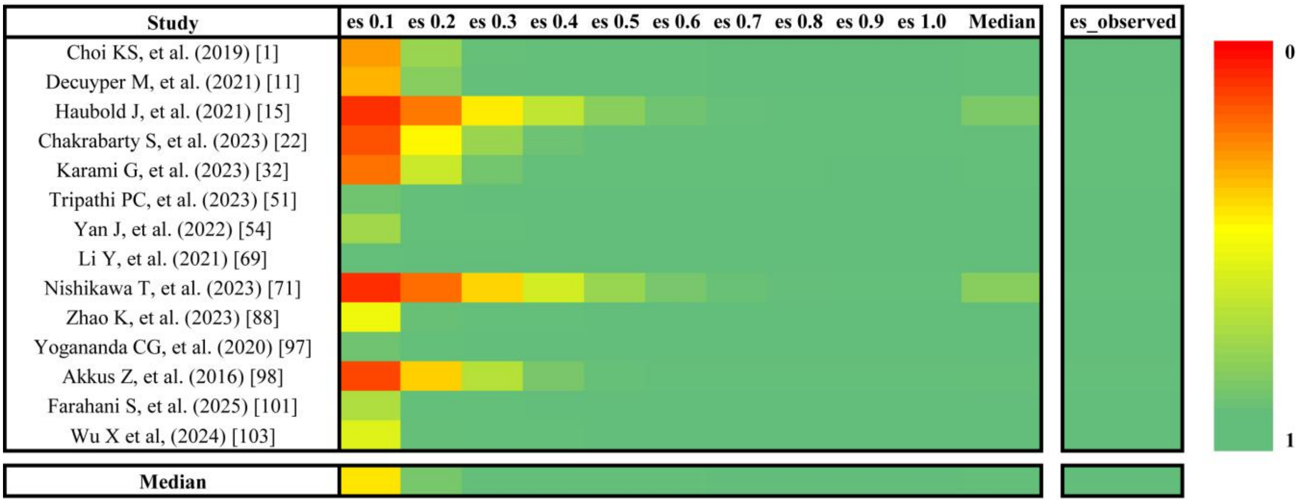

Figure 29. Heatmap of statistical power for 1p/19q prediction in the internal validation groups, indicating a high detection capability for larger sensitivities in included studies but relatively lower power for detecting smaller sensitivity measures in some studies. Greener areas represent higher statistical power for corresponding effect sizes (see color bar). **Abbreviation:** es, effect size.

11.3.2. Pooled specificity estimate

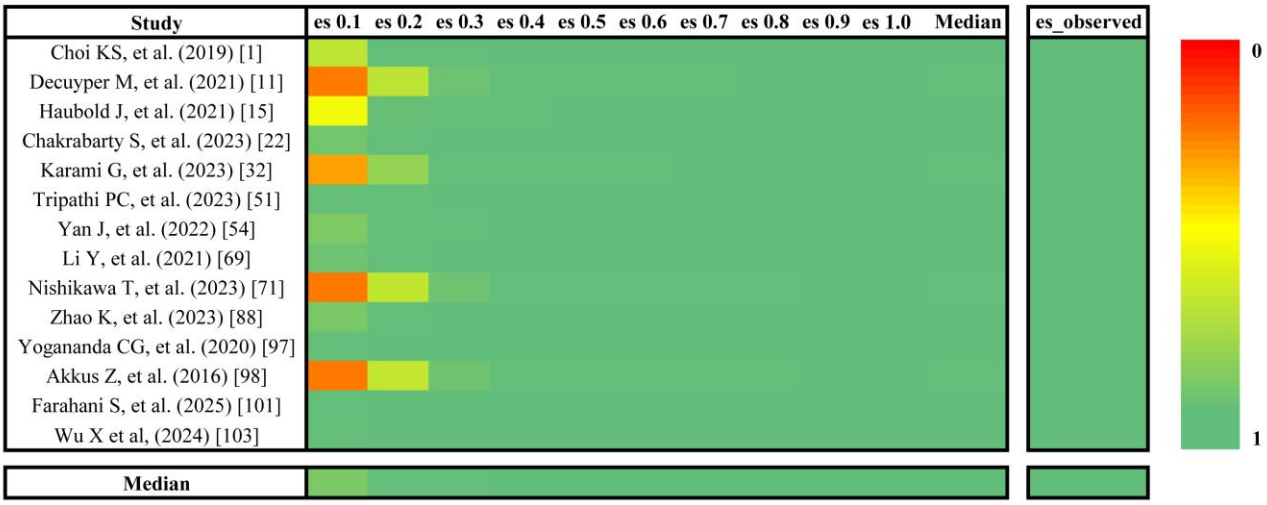

Figure 30. Heatmap of statistical power for 1p/19q prediction in the internal validation groups, indicating a high detection capability for larger specificities in included studies but relatively lower power for detecting smaller specificity measures in some studies. Greener areas represent higher statistical power for corresponding effect sizes (see color bar). **Abbreviation:** es, effect size.

11.4. 1p/19q Codeletion Prediction in Test Cohorts

11.4.1. Pooled sensitivity estimate

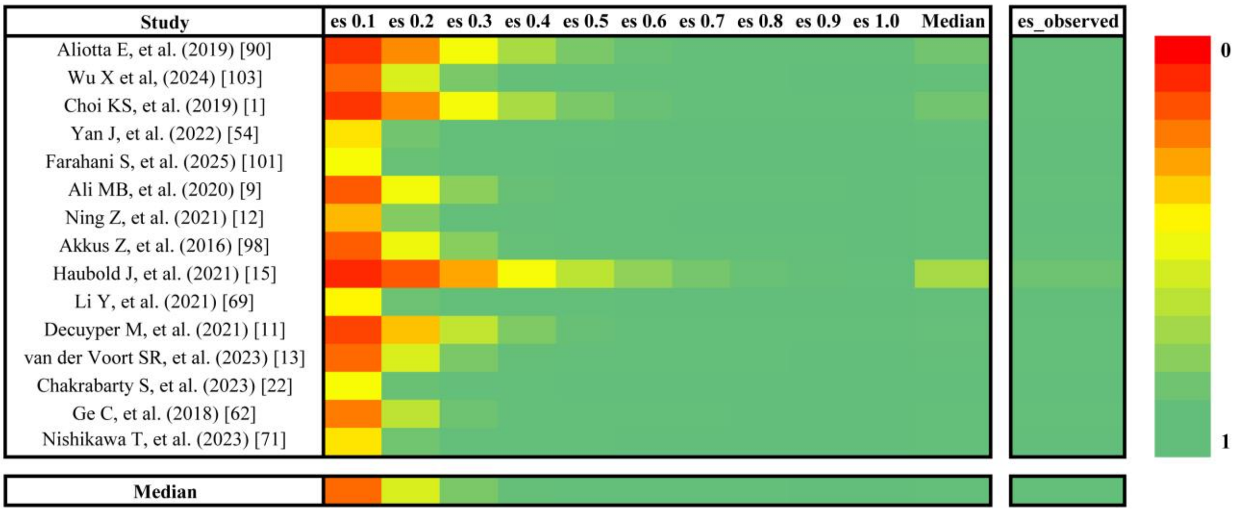

Figure 31. Heatmap of statistical power for 1p/19q prediction in training groups, indicating a high detection capability for larger sensitivities in included studies but relatively lower power for detecting smaller sensitivity measures in some studies. Greener areas represent higher statistical power for corresponding effect sizes (see color bar). **Abbreviation:** es, effect size.

11.4.2. Pooled specificity estimate

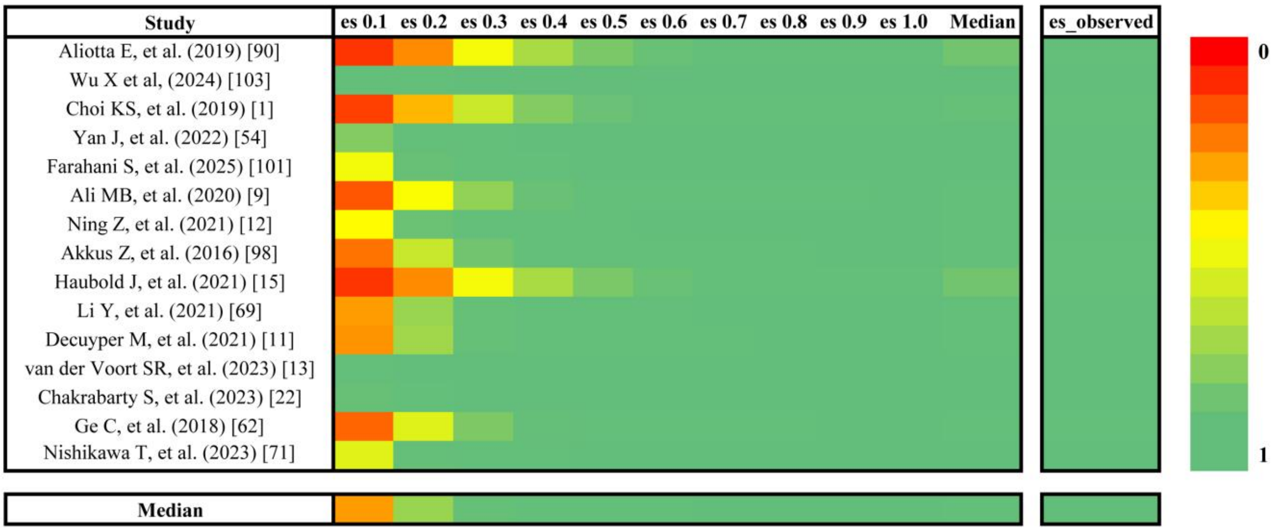

Figure 32. Heatmap of statistical power for 1p/19q prediction in the internal validation groups, indicating a high detection capability for larger specificities in included studies but relatively lower power for detecting smaller specificity measures in some studies. Greener areas represent higher statistical power for corresponding effect sizes (see color bar). **Abbreviation:** es, effect size.

12. Amendment to Registered Protocol

This study is registered in PROSPERO (CRD42024542505) under the protocol titled "Radiogenomic Predictors in Glioma Patients Using Deep Learning Methods Applied to MRI: A Systematic Review and Meta-Analysis." The protocol aimed to investigate deep learning models for identifying all molecular markers on MRI.

Given the extensive data extracted across multiple genetic markers and the inclusion of meta-analyses and meta-regressions on various moderators (e.g., MRI sequences, deep learning architectures, datasets), we divided the review into two separate manuscripts. The first, titled "Diagnostic Performance of Deep Learning for Predicting Glioma Isocitrate Dehydrogenase and 1p/19q Co-deletion in MRI," was completed on June 7, 2024, and updated on March 28, 2025. The second article has been published and is available at *https://doi.org/10.3390/diagnostics15070797*.

13. PRISMA Checklist

Section and Topic	Item #	Checklist item	Reported on
**TITLE**			
Title	1	Identify the report as a systematic review.	Not applicable due to the journal's word count limitations.
**ABSTRACT**			
Abstract	2	See the PRISMA 2020 for Abstracts checklist.	Manuscript, page 1
**INTRODUCTION**			
Rationale	3	Describe the rationale for the review in the context of existing knowledge.	Manuscript, page 4
Objectives	4	Provide an explicit statement of the objective(s) or question(s) the review addresses.	Manuscript, page 4
**METHODS**			
Eligibility criteria	5	Specify the inclusion and exclusion criteria for the review and how studies were grouped for the syntheses.	Manuscript, page 5
Information sources	6	Specify all databases, registers, websites, organisations, reference lists and other sources searched or consulted to identify studies. Specify the date when each source was last searched or consulted.	Manuscript, page 5
Search strategy	7	Present the full search strategies for all databases, registers and websites, including any filters and limits used.	Supplementary file, pages 4-8
Selection process	8	Specify the methods used to decide whether a study met the inclusion criteria of the review, including how many reviewers screened each record and each report retrieved, whether they worked independently, and if applicable, details of automation tools used in the process.	Manuscript, page 5
Data collection process	9	Specify the methods used to collect data from reports, including how many reviewers collected data from each report, whether they worked independently, any processes for obtaining or confirming data from study investigators, and if applicable, details of automation tools used in the process.	Manuscript, page 5
Data items	10a	List and define all outcomes for which data were sought. Specify whether all results that were compatible with each outcome domain in each study were sought (e.g. for all measures, time points, analyses), and if not, the methods used to decide which results to collect.	Manuscript, page 5
	10b	List and define all other variables for which data were sought (e.g. participant and intervention characteristics, funding sources). Describe any assumptions made about any missing or unclear information.	Manuscript, page 5; Supplementary file, page 9
Study risk of bias assessment	11	Specify the methods used to assess risk of bias in the included studies, including details of the tool(s) used, how many reviewers assessed each study and whether they worked independently, and if applicable, details of automation tools used in the process.	Manuscript, page 6; Supplementary file, page 10
Effect measures	12	Specify for each outcome the effect measure(s) (e.g. risk ratio, mean difference) used in the synthesis or presentation of results.	Manuscript, page 6
Synthesis methods	13a	Describe the processes used to decide which studies were eligible for each synthesis (e.g. tabulating the study intervention characteristics and comparing against the planned groups for each synthesis (item #5)).	Manuscript, page 6
	13b	Describe any methods required to prepare the data for presentation or synthesis, such as handling of missing summary statistics, or data conversions.	Manuscript, page 5
	13c	Describe any methods used to tabulate or visually display results of individual studies and syntheses.	Manuscript, pages 5, 6

Section and Topic	Item #	Checklist item	Reported on
	13d	Describe any methods used to synthesize results and provide a rationale for the choice(s). If meta-analysis was performed, describe the model(s), method(s) to identify the presence and extent of statistical heterogeneity, and software package(s) used.	Manuscript, page 6
	13e	Describe any methods used to explore possible causes of heterogeneity among study results (e.g. subgroup analysis, meta-regression).	Manuscript, page 6
	13f	Describe any sensitivity analyses conducted to assess robustness of the synthesized results.	Manuscript, page 6
Reporting bias assessment	14	Describe any methods used to assess risk of bias due to missing results in a synthesis (arising from reporting biases).	Manuscript, page 6 (QUADAS-2 tool); Supplementary file, page 10
Certainty assessment	15	Describe any methods used to assess certainty (or confidence) in the body of evidence for an outcome.	Manuscript, page 6 (RQS), Supplementary file, page 15
**RESULTS**			
Study selection	16a	Describe the results of the search and selection process, from the number of records identified in the search to the number of studies included in the review, ideally using a flow diagram.	Manuscript Figure 1
	16b	Cite studies that might appear to meet the inclusion criteria, but which were excluded, and explain why they were excluded.	Manuscript, Lines 134-137; Supplementary file, pages 19-21
Study characteristics	17	Cite each included study and present its characteristics.	Manuscript Table 1
Risk of bias in studies	18	Present assessments of risk of bias for each included study.	Manuscript page 9, Supplementary file – Pages 11-14
Results of individual studies	19	For all outcomes, present, for each study: (a) summary statistics for each group (where appropriate) and (b) an effect estimate and its precision (e.g. confidence/credible interval), ideally using structured tables or plots.	Manuscript Table 1; Supplementary file Table 5, pages 60-67
Results of syntheses	20a	For each synthesis, briefly summarise the characteristics and risk of bias among contributing studies.	Manuscript, pages 7-9; Supplementary file, pages 16-18
	20b	Present results of all statistical syntheses conducted. If meta-analysis was done, present for each the summary estimate and its precision (e.g. confidence/credible interval) and measures of statistical heterogeneity. If comparing groups, describe the direction of the effect.	Manuscript, pages 9-11, Figures 3 and 4, Table 2; Supplementary file - pages 48-59
	20c	Present results of all investigations of possible causes of heterogeneity among study results.	Manuscript, pages 10, 11, Tables 3 and 4
	20d	Present results of all sensitivity analyses conducted to assess the robustness of the synthesized results.	Supplementary file, pages 40-47
Reporting biases	21	Present assessments of risk of bias due to missing results (arising from reporting biases) for each synthesis assessed.	Manuscript, page 9; Supplementary file, pages 28-39
Certainty of evidence	22	Present assessments of certainty (or confidence) in the body of evidence for each outcome assessed.	Manuscript, page 9; Supplementary file, pages 60-67
**DISCUSSION**			
Discussion	23a	Provide a general interpretation of the results in the context of other evidence.	Manuscript, pages 11-14
	23b	Discuss any limitations of the evidence included in the review.	Manuscript, page 14

Section and Topic	Item #	Checklist item	Reported on
	23c	Discuss any limitations of the review processes used.	Manuscript, page 14
	23d	Discuss implications of the results for practice, policy, and future research.	Manuscript, pages 12-14
**OTHER INFORMATION**			
Registration and protocol	24a	Provide registration information for the review, including register name and registration number, or state that the review was not registered.	CRD42024542505
	24b	Indicate where the review protocol can be accessed, or state that a protocol was not prepared.	https://www.crd.york.ac.uk/PROSPERO/
	24c	Describe and explain any amendments to information provided at registration or in the protocol.	Supplementary file, page 68
Support	25	Describe sources of financial or non-financial support for the review, and the role of the funders or sponsors in the review.	No financial support received.
Competing interests	26	Declare any competing interests of review authors.	No conflict of interest.
Availability of data, code and other materials	27	Report which of the following are publicly available and where they can be found: template data collection forms; data extracted from included studies; data used for all analyses; analytic code; any other materials used in the review.	- Template data collection forms: Supplementary file, page 9<br>- Data extracted from included studies: Available in Manuscript Table 1.<br>- Data used for all analyses: Not Applicable<br>- Analytic code: Not Applicable<br>- Other materials used in the review: Included in the supplementary file.

From: Page MJ, McKenzie JE, Bossuyt PM, Boutron I, Hoffmann TC, Mulrow CD, et al. The PRISMA 2020 statement: an updated guideline for reporting systematic reviews. BMJ 2021;372:n71. doi: 10.1136/bmj.n71

For more information, visit: http://www.prisma-statement.org/

14. References

1. Choi KS, Choi SH, Jeong B (2019) Prediction of IDH genotype in gliomas with dynamic susceptibility contrast perfusion MR imaging using an explainable recurrent neural network. Neuro-Oncol 21:1197–1209. https://doi.org/10.1093/neuonc/noz095

2. Fukuma R, Yanagisawa T, Kinoshita M, et al (2019) Prediction of IDH and TERT promoter mutations in low-grade glioma from magnetic resonance images using a convolutional neural network. Sci Rep 9:20311. https://doi.org/10.1038/s41598-019-56767-3

3. Ge C, Gu IY-H, Jakola AS, Yang J (2020) Enlarged Training Dataset by Pairwise GANs for Molecular-Based Brain Tumor Classification. IEEE ACCESS 8:22560–22570. https://doi.org/10.1109/ACCESS.2020.2969805

4. Li Z, Wang Y, Yu J, et al (2017) Deep Learning based Radiomics (DLR) and its usage in noninvasive IDH1 prediction for low grade glioma. Sci Rep 7:5467. https://doi.org/10.1038/s41598-017-05848-2

5. Liang S, Zhang R, Liang D, et al (2018) Multimodal 3D DenseNet for IDH genotype prediction in gliomas. Genes 9:382. https://doi.org/10.3390/genes9080382

6. Wu G, Chen Y, Wang Y, et al (2017). Sparse Sparse representation-based radiomics for the diagnosis of brain tumors. IEEE transactions on medical imaging, 37(4), 893-905. https://doi.org/10.1109/TMI.2017.2776967

7. Kim D, Wang N, Ravikumar V, et al (2019) Prediction of 1p/19q Codeletion in Diffuse Glioma Patients Using Pre-operative Multiparametric Magnetic Resonance Imaging. Front Comput Neurosci 13:52. https://doi.org/10.3389/fncom.2019.00052

8. Chang P, Grinband J, Weinberg BD, et al (2018) Deep-Learning Convolutional Neural Networks Accurately Classify Genetic Mutations in Gliomas. Am J Neuroradiol 39:1201–1207. https://doi.org/10.3174/ajnr.A5667

9. Ali MB, Gu IY-H, Berger MS, et al (2020) Domain mapping and deep learning from multiple mri clinical datasets for prediction of molecular subtypes in low grade gliomas. Brain Sci 10:1–20. https://doi.org/10.3390/brainsci10070463

10. Tang Z, Xu Y, Jin L, et al (2020) Deep learning of imaging phenotype and genotype for predicting overall survival time of glioblastoma patients. IEEE Trans Med Imaging 39:2100–2109. https://doi.org/10.1109/TMI.2020.2964310

11. Decuyper M, Bonte S, Deblaere K, Van Holen R (2021) Automated MRI based pipeline for segmentation and prediction of grade, IDH mutation and 1p19q co-deletion in glioma. Comput Med Imaging Graph 88:101831. https://doi.org/10.1016/j.compmedimag.2020.101831

12. Ning Z, Tu C, Di X, et al (2021) Deep cross-view co-regularized representation learning for glioma subtype identification. Med Image Anal 73, 102160. https://doi.org/10.1016/j.media.2021.102160

13. van der Voort SR, Incekara F, Wijnenga MM, et al (2023) Combined molecular subtyping, grading, and segmentation of glioma using multi-task deep learning. Neuro-Oncol 25:279–289. https://doi.org/10.1093/neuonc/noac166

14. Cluceru J, Interian Y, Phillips JJ, et al (2022) Improving the noninvasive classification of glioma genetic subtype with deep learning and diffusion-weighted imaging. Neuro-Oncol 24:639–652. https://doi.org/10.1093/neuonc/noab238

15. Haubold J, Hosch R, Parmar V, et al (2021) Fully Automated MR Based Virtual Biopsy of Cerebral Gliomas. Cancers 13:6186. https://doi.org/10.3390/cancers13246186

16. Tupe-Waghmare P, Malpure P, Kotecha K, et al (2021) Comprehensive genomic subtyping of glioma using semi-supervised multi-task deep learning on multimodal MRI. IEEE Access 9:167900–167910. https://doi.org/10.1109/ACCESS.2021.3136293

17. Matsui Y, Maruyama T, Nitta M, et al (2020) Prediction of lower-grade glioma molecular subtypes using deep learning. J Neurooncol 146:321–327. https://doi.org/10.1007/s11060-019-03376-9

18. Chang K, Bai HX, Zhou H, et al (2018) Residual Convolutional Neural Network for the Determination of IDH Status in Low- and High-Grade Gliomas from MR Imaging. Clin Cancer Res Off J Am Assoc Cancer Res 24:1073–1081. https://doi.org/10.1158/1078-0432.CCR-17-2236

19. Calabrese E, Villanueva-Meyer JE, Cha S (2020) A fully automated artificial intelligence method for non-invasive, imaging-based identification of genetic alterations in glioblastomas. Sci Rep 10:11852. https://doi.org/10.1038/s41598-020-68857-8

20. Ai L, Bai W, Li M (2022) TDABNet: Three-directional attention block network for the determination of IDH status in low-and high-grade gliomas from MRI. Biomed Signal Process Control 75:103574. https://doi.org/10.1016/j.bspc.2022.103574

21. Chaddad A, Hassan L, Katib Y (2023) A texture-based method for predicting molecular markers and survival outcome in lower grade glioma. Appl Intell 53:24724–24738. https://doi.org/10.1007/s10489-023-04844-6

22. Chakrabarty S, LaMontagne P, Shimony J, et al (2023) MRI-based classification of IDH mutation and 1p/19q codeletion status of gliomas using a 2.5 D hybrid multi-task convolutional neural network. Neuro-Oncol Adv 5:vdad023. https://doi.org/10.1093/noajnl/vdad023

23. Chakrabarty S, LaMontagne P, Shimony J, et al (2023) Non-invasive classification of IDH mutation status of gliomas from multi-modal MRI using a 3D convolutional neural network. SPIE, pp 175–183. https://doi.org/10.1117/12.2651391

24. Chen Q, Wang L, Xing Z, et al (2023) Deep wavelet scattering orthogonal fusion network for glioma IDH mutation status prediction. Comput Biol Med 166:107493. https://doi.org/10.1016/j.compbiomed.2023.107493

25. Chu W, Zhou Y, Cai S, et al (2024) A Comprehensive Multi-modal Domain Adaptative Aid Framework for Brain Tumor Diagnosis. In Chinese Conference on Pattern Recognition and Computer Vision (PRCV) (pp. 382-394). Singapore: Springer Nature Singapore. https://doi.org/10.1007/978-981-99-8558-6_32

26. Buz-Yalug B, Turhan G, Cetin AI, et al (2024) Identification of IDH and TERTp mutations using dynamic susceptibility contrast MRI with deep learning in 162 gliomas. Eur J Radiol 170:111257. https://doi.org/10.1016/j.ejrad.2023.11125

27. Calabrese E, Rudie JD, Rauschecker AM, et al (2022) Combining radiomics and deep convolutional neural network features from preoperative MRI for predicting clinically relevant genetic biomarkers in glioblastoma. Neuro-Oncol Adv 4:. https://doi.org/10.1093/noajnl/vdac060

28. Cheng J, Liu J, Kuang H, Wang J (2022) A fully automated multimodal MRI-based multi-task learning for glioma segmentation and IDH genotyping. IEEE Trans Med Imaging 41:1520–1532. https://doi.org/10.1109/TMI.2022.3142321

29. Choi Y, Nam Y, Lee YS, et al (2020) IDH1 mutation prediction using MR-based radiomics in glioblastoma: comparison between manual and fully automated deep learning-based approach of tumor segmentation. Eur J Radiol 128:109031. https://doi.org/10.1016/j.ejrad.2020.109031

30. Choi YS, Bae S, Chang JH, et al (2021) Fully automated hybrid approach to predict the IDH mutation status of gliomas via deep learning and radiomics. Neuro-Oncol 23:304–313. https://doi.org/10.1093/neuonc/noaa177

31. GORE S, JAGTAP J (2021) MRI based genomic analysis of glioma using three pathway deep convolutionalneural network for IDH classification. Turk J Electr Eng Comput Sci 29:2728–2741. https://doi.org/10.3906/elk-2104-180

32. Karami G., Pascuzzo R., Figini M., et al (2023) Combining Multi-Shell Diffusion with Conventional MRI Improves Molecular Diagnosis of Diffuse Gliomas with Deep Learning. Cancers 15:482. https://doi.org/10.3390/cancers15020482

33. Liu J, Cong C, Zhang J, et al (2024) Multimodel habitats constructed by perfusion and/or diffusion MRI predict isocitrate dehydrogenase mutation status and prognosis in high-grade gliomas. Clin Radiol 79:e127–e136. https://doi.org/10.1016/j.crad.2023.09.025

34. McHugh H, Safaei S, Maso Talou GD, et al (2023) IDH and 1p19q Diagnosis in Diffuse Glioma from Preoperative MRI Using Artificial Intelligence. medRxiv 2023–04. https://doi.org/10.1101/2023.04.26.21267661

35. Moon HH, Jeong J, Park JE, et al (2024) Generative AI in glioma: ensuring diversity in training image phenotypes to improve diagnostic performance for IDH mutation prediction. Neuro-Oncol 26:1124–1135. https://doi.org/10.1093/neuonc/noae012

36. Nalawade S, Murugesan GK, Vejdani-Jahromi M, et al (2019) Classification of brain tumor isocitrate dehydrogenase status using MRI and deep learning. J Med Imaging 6:046003–046003. https://doi.org/10.1117/1.JMI.6.4.046003

37. Nalawade SS, Yu FF, Bangalore Yogananda CG, et al (2022) Brain tumor IDH, 1p/19q, and MGMT molecular classification using MRI-based deep learning: an initial study on the effect of motion and motion correction. J Med Imaging 9:016001–016001. https://doi.org/10.1117/1.JMI.9.1.016001

38. Pasquini L, Napolitano A, Tagliente E, et al (2021) Deep learning can differentiate IDH-mutant from IDH-wild GBM. J Pers Med 11:290. https://doi.org/10.3390/jpm11040290

39. Rui W, Zhang S, Shi H, et al (2023) Deep learning-assisted quantitative susceptibility mapping as a tool for grading and molecular subtyping of gliomas. Phenomics 3:243–254. https://doi.org/10.1007/s43657-022-00087-6

40. Safari M, Beiki M, Ameri A, et al (2022) Shuffle-ResNet: Deep learning for predicting LGG IDH1 mutation from multicenter anatomical MRI sequences. Biomed Phys Eng Express 8:065036. https://doi.org/10.1088/2057-1976/ac9fc8

41. Zhang J, Cao J, Tang F, et al (2023) Multi-level feature exploration and fusion network for prediction of IDH status in gliomas from MRI. IEEE J Biomed Health Inform 28:42–53. https://doi.org/10.1109/JBHI.2023.3279433

42. Zhang H, Fan X, Zhang J, et al (2023) Deep-learning and conventional radiomics to predict IDH genotyping status based on magnetic resonance imaging data in adult diffuse glioma. Front Oncol 13:1143688. https://doi.org/10.3389/fonc.2023.1143688

43. Bangalore Yogananda CG, Wagner BC, Truong NC, et al (2023) MRI-based deep learning method for classification of IDH mutation status. Bioengineering 10:1045. https://doi.org/10.3390/bioengineering10091045

44. Zeng H, Xing Z, Gao F, et al (2022) A multimodal domain adaptive segmentation framework for IDH genotype prediction. Int J Comput Assist Radiol Surg 17:1923–1931. https://doi.org/10.1007/s11548-022-02700-5

45. Xu Q, Xu QQ, Shi N, et al (2022) A multitask classification framework based on vision transformer for predicting molecular expressions of glioma. Eur J Radiol 157:110560. https://doi.org/10.1016/j.ejrad.2022.110560

46. Wu J, Xu Q, Shen Y, et al (2022) Swin transformer improves the IDH mutation status prediction of gliomas free of MRI-based tumor segmentation. J Clin Med 11:4625. https://doi.org/10.3390/jcm11154625

47. Wei Y, Li Y, Chen X, et al (2021) Predicting isocitrate dehydrogenase mutation status in glioma using structural brain networks and graph neural networks. Springer, pp 140–150. https://doi.org/10.1007/978-3-031-08999-2_11

48. Wang Y, Wang Y, Guo C, et al (2021) SGPNet: A Three-Dimensional Multitask Residual Framework for Segmentation and IDH Genotype Prediction of Gliomas. Comput Intell Neurosci 2021:5520281. https://doi.org/10.1155/2021/5520281

49. Wei Y, Li C, Chen X, et al (2022) Collaborative learning of images and geometrics for predicting isocitrate dehydrogenase status of glioma. IEEE, pp 1–4. https://doi.org/10.1109/ISBI52829.2022.9761407

50. Wei Y, Chen X, Zhu L, et al (2023) Multi-Modal Learning for Predicting the Genotype of Glioma. IEEE Trans Med Imaging 42:3167–3178. https://doi.org/10.1109/TMI.2023.3244038

51. Tripathi PC, Bag S (2023) An Attention-Guided CNN Framework for Segmentation and Grading of Glioma Using 3D MRI Scans. IEEE-ACM Trans Comput Biol Bioinforma 20:1890–1904. https://doi.org/10.1109/TCBB.2022.3220902

52. Shi X, Li Y, Chen Y-W, et al (2023) An Intra-and Inter-Modality Fusion Model with Invariant-and Specific-Constraints Using MR Images for Prediction of Glioma Isocitrate Dehydrogenase Mutation Status. J Image Graph Kingd 11:321–329. https://doi.org/10.18178/joig.11.4.321-329

53. Shi X, Li Y, Cheng J, et al (2023) Multi-task Model for Glioma Segmentation and Isocitrate Dehydrogenase Status Prediction Using Global and Local Features. IEEE, pp 1–5. https://doi.org/10.1109/EMBC40787.2023.10340355

54. Yan J, Zhang S, Sun Q, et al (2022) Predicting 1p/19q co-deletion status from magnetic resonance imaging using deep learning in adult-type diffuse lower-grade gliomas: a discovery and validation study. Lab Invest 102:154–159. https://doi.org/10.1038/s41374-021-00692-5

55. Kihira S, Mei X, Mahmoudi K, et al (2022) U-Net based segmentation and characterization of gliomas. Cancers 14:4457. https://doi.org/10.3390/cancers14184457

56. Sohn B, An C, Kim D, et al (2021) Radiomics-based prediction of multiple gene alteration incorporating mutual genetic information in glioblastoma and grade 4 astrocytoma, IDH-mutant. J Neurooncol 155:267–276. https://doi.org/10.1007/s11060-021-03870-z

57. Buda M, Saha A, Mazurowski MA (2019) Association of genomic subtypes of lower-grade gliomas with shape features automatically extracted by a deep learning algorithm. Comput Biol Med 109:218–225. https://doi.org/10.1016/j.compbiomed.2019.05.002

58. Ali MB, Gu IY-H, Berger MS, Jakola AS (2023) A novel federated deep learning scheme for glioma and its subtype classification. Front Neurosci 17:1181703. https://doi.org/10.3389/fnins.2023.1181703

59. Chen M, Zhang M, Yin L, et al (2024) Medical image foundation models in assisting diagnosis of brain tumors: a pilot study. Eur Radiol 34:6667–6679. https://doi.org/10.1007/s00330-024-10728-1

60. Elyassirad D, Gheiji B, Vatanparast M, et al (2024) Comparative Analysis of 2D and 3D ResNet Architectures for IDH and MGMT Mutation Detection in Glioma Patients. https://doi.org/10.48550/arXiv.2412.21091

61. Fayyaz M, Chaudhry N, Choudhary R (2023) Classification of Isocitrate Dehydrogenase (IDH) Mutation Status in Gliomas Using Transfer Learning. Pak J Sci Res PJOSR 3(2).

62. Ge C, Gu IY-H, Jakola AS, Yang J (2018) Deep learning and multi-sensor fusion for glioma classification using multistream 2D convolutional networks. IEEE, pp 5894–5897. https://doi.org/10.1109/EMBC.2018.8513556

63. Gómez Vecchio T, Neimantaite A, Thurin E, et al (2024) Clinical application of machine-based deep learning in patients with radiologically presumed adult-type diffuse glioma grades 2 or 3. Neuro-Oncol Adv 6:vdae192. https://doi.org/10.1093/noajnl/vdae192

64. Hosseini SA, Hosseini E, Hajianfar G, et al (2023) MRI-based radiomics combined with deep learning for distinguishing IDH-mutant WHO grade 4 astrocytomas from IDH-wild-type glioblastomas. Cancers 15:951. https://doi.org/10.3390/cancers15030951

65. Jeon YH, Choi KS, Lee KH, et al (2025) Deep learning-based quantification of T2-FLAIR mismatch sign: extending IDH mutation prediction in adult-type diffuse lower-grade glioma. Eur Radiol 1–10. https://doi.org/10.1007/s00330-025-11475-7

66. Jian J, Xu L, Gong C, et al (2025) One class classification-empowered radiomics for noninvasively accurate prediction of glioma isocitrate dehydrogenase mutation using multiparametric magnetic resonance imaging. Clin Radiol 106866. https://doi.org/10.1016/j.crad.2025.106866

67. Li D, Hu W, Ma L, et al (2025) Deep learning radiomics nomograms predict Isocitrate dehydrogenase (IDH) genotypes in brain glioma: A multicenter study. Magn Reson Imaging 117:110314. https://doi.org/10.1016/j.mri.2024.110314

68. Li X, Xu Y, Xiang F, et al (2021) Prediction of IDH mutation status of glioma based on multimodal MRI images. pp 39–44. https://doi.org/10.1145/3468945.34689

69. Li Y, Wei D, Liu X, et al (2022) Molecular subtyping of diffuse gliomas using magnetic resonance imaging: comparison and correlation between radiomics and deep learning. Eur Radiol 32:747–758. https://doi.org/10.1007/s00330-021-08237-6

70. Lost J, Ashraf N, Jekel L, et al (2024) Enhancing clinical decision-making: An externally validated machine learning model for predicting isocitrate dehydrogenase mutation in gliomas using radiomics from presurgical magnetic resonance imaging. Neuro-Oncol Adv 6:vdae157. https://doi.org/10.1093/noajnl/vdae157

71. Nishikawa T, Ohka F, Aoki K, et al (2023) Easy-to-use machine learning system for the prediction of IDH mutation and 1p/19q codeletion using MRI images of adult-type diffuse gliomas. Brain Tumor Pathol 40:85–92. https://doi.org/10.1007/s10014-023-00459-4

72. Park JE, Eun D, Kim HS, et al (2021) Generative adversarial network for glioblastoma ensures morphologic variations and improves diagnostic model for isocitrate dehydrogenase mutant type. Sci Rep 11:9912. https://doi.org/10.1038/s41598-021-89477-w

73. Sacli-Bilmez B, Bas A, Danyeli AE, et al (2025) Detecting IDH and TERTp mutations in diffuse gliomas using 1H-MRS with attention deep-shallow networks. Comput Biol Med 186:109736. https://doi.org/10.1016/j.compbiomed.2025.109736

74. Sairam V, Bhaskar N, Tupe-Waghmare P (2023) Automated Glioma Grading and IDH Mutation Status Prediction Using CNN-Based Deep Learning Models. Springer, pp 391–400. https://doi.org/10.1007/978-981-97-4650-7_29

75. Santinha J, Katsaros V, Stranjalis G, et al (2024) Development of end-to-end AI–based MRI image analysis system for predicting IDH mutation status of patients with gliomas: multicentric validation. J Imaging Inform Med 37:31–44. https://doi.org/10.1007/s10278-023-00918-6

76. Shi X, Zhang X, Iwamoto Y, et al (2022) An Intra-and Inter-Modality Fusion Model Using MR Images for

Prediction of Glioma Isocitrate Dehydrogenase (IDH) Mutation. IEEE, pp 198–202. https://doi.org/10.1109/EMBC48229.2022.9871800

77. Stadlbauer A, Nikolic K, Oberndorfer S, et al (2024) Machine learning-based prediction of glioma IDH gene mutation status using physio-metabolic MRI of oxygen metabolism and neovascularization (A bicenter study). Cancers 16:1102. https://doi.org/10.3390/cancers16061102

78. Taha B, Li T, Boley D, et al (2020) Detection of Isocitrate Dehydrogenase Mutated Glioblastomas Through Anomaly Detection Analytics: A Pilot Study. Neurosurgery 67. https://doi.org/10.1093/neuros/nyaa447_840

79. Usuzaki T, Inamori R, Shizukuishi T, et al (2024) Predicting isocitrate dehydrogenase status among adult patients with diffuse glioma using patient characteristics, radiomic features, and magnetic resonance imaging: Multi-modal analysis by variable vision transformer. Magn Reson Imaging 111:266–276. https://doi.org/10.1016/j.mri.2024.05.012

80. Wang Y, Gao A, Yang H, et al (2025) Using partially shared radiomics features to simultaneously identify isocitrate dehydrogenase mutation status and epilepsy in glioma patients from MRI images. Sci Rep 15:3591. https://doi.org/10.1038/s41598-025-87778-y

81. Wankhede DS, Shelke CJ (2022) An Investigative approach on the prediction of Isocitrate dehydrogenase (idh1) mutations and co-deletion of 1p19q in glioma brain tumors. Springer, pp 188–198. https://doi.org/10.1007/978-3-031-35507-3_19

82. Yang Z, Zhang P, Ding Y, et al (2025) Magnetic resonance imaging-based deep learning for predicting subtypes of glioma. Front Neurol 16:1518815. https://doi.org/10.1109/JBHI.2023.3279433

83. Yu D, Zhong Q, Xiao Y, et al (2024) Combination of MRI-based prediction and CRISPR/Cas12a-based detection for IDH genotyping in glioma. NPJ Precis Oncol 8:140. https://doi.org/10.1038/s41698-024-00632-8

84. Yuan J, Siakallis L, Li HB, et al (2024) Structural-and DTI-MRI enable automated prediction of IDH Mutation Status in CNS WHO Grade 2–4 glioma patients: a deep Radiomics Approach. BMC Med Imaging 24:104. https://doi.org/10.1186/s12880-024-01274-9

85. Yuan Y, Yu Y, Chang J, et al (2023) Convolutional neural network to predict IDH mutation status in glioma from chemical exchange saturation transfer imaging at 7 Tesla. Front Oncol 13:1134626. https://doi.org/10.3389/fonc.2023.1134626

86. Zhang S, Sun H, Su X, et al (2021) Automated machine learning to predict the co-occurrence of isocitrate dehydrogenase mutations and O6-methylguanine-DNA methyltransferase promoter methylation in patients with gliomas. J Magn Reson Imaging 54:197–205. https://doi.org/10.1002/jmri.27498

87. Zhang X, Iwamoto Y, Cheng J, et al (2021) IDH mutation status prediction by modality-self attention network. Springer, pp 51–57. https://doi.org/10.1007/978-981-16-3013-2_5

88. Zhao K, Li B, Zhang K, et al (2023) Automatic 1p/19q co-deletion identification of gliomas by MRI using deep learning U-net network. Comput Electr Eng 105:108482. https://doi.org/10.1016/j.compeleceng.2022.108482

89. Zhu Z, Shen J, Liang X, et al (2024) Radiomics for predicting grades, isocitrate dehydrogenase mutation, and oxygen 6-methylguanine-DNA methyltransferase promoter methylation of adult diffuse gliomas: combination of structural MRI, apparent diffusion coefficient, and susceptibility-weighted imaging. Quant Imaging Med Surg 14:9276. https://doi.org/10.21037/qims-24-1110

90. Aliotta E, Nourzadeh H, Batchala PP, et al (2019) Molecular Subtype Classification in Lower-Grade Glioma with Accelerated DTI. AJNR Am J Neuroradiol 40:1458–1463. https://doi.org/10.3174/ajnr.A6162

91. Alom Z, Tran QT, Bag AK, et al (2023) Predicting methylation class from diffusely infiltrating adult gliomas using multimodality MRI data. NEURO-Oncol Adv 5:vdad045. https://doi.org/10.1093/noajnl/vdad045

92. Rosas González S, Zemmoura I, Tauber C (2019) Deep convolutional neural network to predict 1p19q co-deletion and IDH1 mutation status from MRI in low grade gliomas. In: IET Conf Publ. Institution of Engineering and Technology, pp 7–11. https://doi.org/10.1049/cp.2019.0240

93. Samani ZR, Parker D, Akbari H, et al (2023) Artificial intelligence-based locoregional markers of brain peritumoral microenvironment. Sci Rep 13:963. https://doi.org/10.1038/s41598-022-26448-9. https://doi.org/10.1038/s41598-022-26448-9

94. Sun X, Li S, Ma C, et al (2024) Glioma Subtype Prediction Based on Radiomics of Tumor and Peritumoral Edema under Automatic Segmentation, 14(1), 27471. https://doi.org/10.1038/s41598-024-79344-9

95. Zhao Y, Liang F, Xu J, et al (2023) Semisupervised adaptive learning models for IDH1 mutation status prediction. https://doi.org/10.21203/rs.3.rs-3595821/v1

96. Zhang L, Liu Z, Lu H, et al Integrated and Layered Diagnosis of Adult-Type Diffuse Gliomas Using a Deep Features-Based Artificial Intelligence Decision Platform from MRI and Whole-Slide Images: A Multi-Center Study. http://dx.doi.org/10.2139/ssrn.4847217

97. Yogananda CGB, Shah BR, Yu FF, et al (2020) A novel fully automated MRI-based deep-learning method for classification of 1p/19q co-deletion status in brain gliomas. Neuro-Oncol Adv 2:vdaa066. https://doi.org/10.1093/noajnl/vdaa066

98. Akkus Z, Ali I, Sedlar J, et al (2016) Predicting 1p19q Chromosomal Deletion of Low-Grade Gliomas from MR Images using Deep Learning. Arxiv. https://doi.org/arXiv:1611.06939

99. Cao S, Hu Z, Xie X, et al (2024) Integrated diagnosis of glioma based on magnetic resonance images with incomplete ground truth labels. Comput Biol Med 180:108968. https://doi.org/10.1016/j.compbiomed.2024.108968

100. 胡振远, 魏炜, 胡文鐘, et al (2023) Prediction of IDH mutations in glioma based on MRI multiparametric image fusion and DenseNet network. Chin J Magn Reson Imaging 14:10–17. https://doi.org/10.12015/issn.1674-8034.2023.07.003

101. Farahani S, Hejazi M, Ieva AD, et al (2025) Towards a Multimodal MRI-Based Foundation Model for Multi-Level Feature Exploration in Segmentation, Molecular Subtyping, and Grading of Glioma. arXiv:2503.06828. https://doi.org/10.48550/arXiv.2503.06828

102. Niu W, Yan J, Hao M, et al (2025) MRI transformer deep learning and radiomics for predicting IDH wild type TERT promoter mutant gliomas. Npj Precis Oncol 9:1–12. https://doi.org/10.1038/s41698-025-00884-y

103. Wu X, Zhang S, Zhang Z, et al (2024) Biologically interpretable multi-task deep learning pipeline predicts molecular alterations, grade, and prognosis in glioma patients. Npj Precis Oncol 8:1–14. https://doi.org/10.1038/s41698-024-00670-2

104. Chen Q, Wang L, Deng Z, et al (2025) Cooperative multi-task learning and interpretable image biomarkers for glioma grading and molecular subtyping. Med Image Anal 101:103435. https://doi.org/10.1016/j.media.2024.103435
